# Characteristics of gut microbiota of term small gestational age infants within 1 week and their relationship with neurodevelopment at 6 months

**DOI:** 10.3389/fmicb.2022.912968

**Published:** 2022-08-24

**Authors:** Xiaona Chen, Zheng Yan, Lili Liu, Rui Zhang, Xiaojiao Zhang, Cheng Peng, Yuehang Geng, Faliang Zhou, Ying Han, Xinlin Hou

**Affiliations:** Department of Pediatrics, Peking University First Hospital, Beijing, China

**Keywords:** gut microbiota, *Bacteroides*, SGA, neonates, neurodevelopment

## Abstract

**Introduction:**

Small for gestational age (SGA) infants are at a higher risk of neurodevelopmental delay than infants appropriate for gestational age (AGA). Previous studies have confirmed that gut microbiota in early life influences subsequent neurodevelopment. However, few studies have reported corresponding data in SGA populations.

**Objective:**

We aimed to evaluate the characteristics of the gut microbiota of term SGA infants and the associations between the gut microbiota in SGA infants and neurodevelopmental outcomes at 6 months of age.

**Methods:**

Fecal samples were collected on days 1, 3, 5, and 7 from term SGA and AGA infants born between June 2020 and June 2021 at the Peking University First Hospital. 16S ribosomal deoxyribonucleic acid amplicon sequencing was used to analyze the fecal microbiota. We followed up for 6 months and used the Ages and Stages Questionnaires-3 (ASQ-3) to evaluate the neurodevelopmental outcomes among SGA infants.

**Results:**

A total of 162 neonates were enrolled, with 41 SGA infants (25.3%) in the study group and 121 AGA infants (74.7%) in the control group. The gut microbial diversity in the SGA group was lower than that in the AGA group on days 1, 3, 5, and 7. Non-metric multidimensional scaling and analysis of similarities showed significant differences between the two groups. The SGA group had increased relative abundances of *Ralstonia* (3, 5, and 7 days) and *Clostridium* (3 and 7 days). The dominant microorganisms of the SGA group were *Ralstonia* on day 1, *Escherichia_Shigella* on days 3 and 7, and *Clostridia* on day 5. We found that the gut microbial diversity of SGA infants with poor communication scores was higher than that of SGA infants with good communication scores on day 3. Fine motor scores were negatively correlated with the relative abundance of *Bacteroides_fragilis* on day 1. A negative correlation was observed between gross motor scores and relative abundance of *Clostridium_saccharobutylicum* on day 7. *Bacteroidota*, *Bacteroidia*, *Bacteroides*, and *Bacteroides_fragilis* were the dominant microorganisms in the good communication score group on day 7. Communication scores were positively correlated with the relative abundance of *Bacteroidota*, *Bacteroides*, and *Bacteroides_fragilis* on day 7.

**Conclusion:**

The gut microbial diversity of term SGA infants was significantly lower in the first week of life than that of term AGA infants. Certain pathogenic and conditional pathogenic bacteria, such as *Escherichia_Shigella*, *Ralstonia* and *Clostridium* increased or formed the dominant microbiota in SGA infants. Alpha diversity, *Bacteroidota*, *Bacteroides*, *Bacteroides_fragilis*, and *Clostridium_saccharobutylicum* found in SGA infants may be associated with neurodevelopmental outcomes at 6 months of age, indicating possible therapeutic targets for clinical intervention.

## Introduction

Small for gestational age (SGA) infants, defined as having a birth weight less than the 10th percentile of the birth weight of the same sex at the same gestational age, comprise a heterogeneous group (Chen et al., 2017; McCowan et al., 2018; Chawla, 2019). The incidence of SGA in China is ∼ 6.5%, ranking fifth worldwide (Lee et al., 2013). The development of each SGA system is imperfect, and the incidence of neurodevelopmental delay is significantly higher than that in appropriate for gestational age (AGA) infants (Sharma et al., 2016; McCowan et al., 2018; Kesavan and Devaskar, 2019). At present, the mechanisms leading to neurodevelopmental delays are unclear.

In recent years, gut microbiota has become a research hotspot in the fields of biology and medicine. Researchers have realized that the gut microbiota plays an important role in digestion, immune response, nutrient absorption, growth, and metabolism. The gut microbiota is involved in the regulation of many diseases such as inflammatory bowel disease, metabolic syndrome, and diabetes (Adak and Khan, 2019; Dabke et al., 2019; Ma et al., 2019; Mentella et al., 2020). Studies have found that microbiota plays a significant role in early neurological development (Carlson et al., 2018; Cohen Kadosh et al., 2021; Seki et al., 2021).

The so-called “gut–brain axis” represents a two-way communication network between the gut microbiota and the brain. The gut–brain axis theory proposes that the gut microbiota participates in the regulation of brain development and maturation, thus impacting brain functions, including anxiety-like behavior, locomotor behavior, social cognition, learning, and working memory (Al-Asmakh et al., 2012; Cryan et al., 2019; Long-Smith et al., 2020; Saurman et al., 2020). Compared with mice with normal gut microbiota, germ-free mice showed more obvious short-term cognitive and working memory impairments, whereas probiotic treatment prevented memory impairment after an inflammatory response in mice (Gareau et al., 2011). Gut microbiota affects various normal psychological processes and phenomena, participating in the pathophysiology of several psychological and neurological diseases (Liang et al., 2018). It has been reported that the gut microbial composition is altered in children with autism (Finegold et al., 2010; Plaza-Díaz et al., 2019; Dan et al., 2020; Saurman et al., 2020; Wong et al., 2022) and adults with Parkinson’s disease (Vascellari et al., 2020; Hirayama and Ohno, 2021) and Alzheimer’s disease (Zhuang et al., 2018; Bostanciklioğlu, 2019). Many studies have shown that probiotics are effective against anxiety, depression, autism spectrum disorder (ASD), and obsessive-compulsive disorder, and can also improve cognitive function, learning, and memory ability (Wang et al., 2016; Eastwood et al., 2021; Kim et al., 2021; Alemohammad et al., 2022). The intake of probiotics may ameliorate neurodegenerative disorders, including Alzheimer’s disease, multiple sclerosis, Parkinson’s disease, and amyotrophic lateral sclerosis (Cheng et al., 2019; Roy Sarkar and Banerjee, 2019).

Research focusing on children has indicated associations between gut microbiota in the first year of life and subsequent early neurodevelopment (Carlson et al., 2018; Sordillo et al., 2019; Tamana et al., 2021). Researchers have found that the alpha diversity of the gut microbiota in 1-year-old children could predict cognitive function at 2 years of age (Carlson et al., 2018). A cohort study found strong evidence of positive associations between *Bacteroidetes* in late infancy and subsequent cognitive and language performance (Tamana et al., 2021). Another cohort study observed an association between the gut microbiome composition of infants aged 3–6 months and communication—personal and social—and fine motor skills at 3 years of age (Sordillo et al., 2019).

At present, there are many studies on the development and establishment of gut microbiota in healthy neonates. However, studies on the characteristics and evolution of the gut microbiota in SGA infants and their relationship with long-term neurodevelopmental outcomes remain scarce. Therefore, the objective of this study was to explore the characteristics of the gut microbiota of SGA infants during the first week of life using high-throughput sequencing technology. Additionally, this study aimed to further explore the potential relationship between gut microbiota and neurodevelopmental prognosis of SGA infants at 6 months of age. The discovery of the effects of specific microbiota on neural development would provide important insights into potential therapeutic targets for the clinical improvement of neurological development in SGA infants.

## Subjects and methods

### Subjects

Term SGA and AGA neonates hospitalized in the pediatric neonatal ward of Peking University First Hospital between June 2020 and June 2021 were recruited for this study.

#### Inclusion criteria

The following inclusion criteria were used: (a) neonates in the study group needed to meet the diagnostic criteria of SGA infants: newborns whose birthweight was less than the 10th percentile of the birth weight of the same sex at the same gestational age (1); (b) gestational age was defined as ≥ 37 and < 42 weeks; (c) neonates without asphyxia, neonatal hypoxic-ischemic encephalopathy, severe intracranial hemorrhage, cerebral infarction, cytomegalovirus infection, recurrent hypoglycemia, bilirubin encephalopathy, and genetic metabolic diseases were enrolled; (d) informed consent was provided by the legal guardian(s); and (e) neonates were only enrolled with the agreement of cooperation with the follow-up by the legal guardian(s).

#### Exclusion criteria

The following exclusion criteria were used: (1) critical clinical conditions, such as sepsis and multiple organ failure; (2) gastrointestinal malformation, abdominal distension, vomiting, diarrhea, bloody stool, necrotizing enterocolitis, and other gastrointestinal diseases within 1 week; and (3) the presence of diseases that might affect neurological development during the follow-up period, such as severe brain trauma, epilepsy, meningitis, and genetic metabolic diseases.

### Methods

#### Data collection

Clinical data, including sex, gestational age, birth weight, mode of delivery, and antibiotic application within 1 week after birth, were collected. Feces produced on postnatal days 1, 3, 5, and 7 were collected. Fecal samples were stored in sterile freezing tubes (Haimen Morder Experimental Equipment Factory) at −20°C and subjected to microbiota analysis within 1 week.

#### Gut microbiota test and analysis

##### Microbiota sequencing

A biological information database was built using an Illumina TruSeq^§^ DNA PCR-Free Sample Preparation Kit. Quality was evaluated with the assistance of the Qubit@ 2.0 and Agilent Bioanalyzer 2100 system. High-throughput sequencing was performed using an Illumina NovaSeq 6000 platform.

##### Bioinformatics analysis

The effective data were obtained by filtering the original data. The sequences were then clustered into operational taxonomic units (OTUs) with 97% identity, and the OTUs sequences were compared with the silva138 database for species annotation to obtain the basic analysis results of the OTUs and taxonomic pedigree for each sample. Finally, the analysis of OTUs, including alpha and beta diversity, was completed according to species annotation.

• Alpha diversity analysis: The richness and diversity of microbiota can be indicated by alpha diversity, wherein Observed species, Chao1, abundance-based coverage estimator (ACE), Shannon, Simpson, and goods coverage are major evaluation indices of alpha diversity. Observed species represents the actual number of OTUs in the sample. The Chao1 and ACE indices use different calculation methods to estimate the number of OTUs in a sample; the higher the number of OTUs, the higher the diversity of the sample. The abundance and uniformity of the gut microbiota can be expressed using the Shannon and Simpson indices. If all the OTUs contained in the sample were the same, the diversity was the lowest; if they were different, the diversity was the highest. The larger the values of the Shannon and Simpson indices, the higher the diversity of the samples. Good coverage index indicates the sequencing depth; the higher the value, the better the sequencing. The closer the value is to 1, the closer the sequencing depth is to cover all bacteria in the test sample. Rarefaction and rank variance curves are common curves that describe the diversity of the samples in the group. The rarefaction curve directly reflects the rationality of the sequencing data and indirectly reflects the richness of species in the sample, whereas the rank variance curve intuitively reflects the richness and uniformity of species in the sample.
• Beta diversity analysis: Beta diversity analysis focuses on the differences in the microbial community composition of different samples, which is used for the analysis of differences between groups. Principal coordinate analysis (PCoA) and non-metric multidimensional scaling (NMDS) directly reflects the differences in community composition between groups based on the distance between samples. Each point in the figure represents a sample; points of the same color belong to the same group, and the distance between points represents the degree of difference. The distance is directly proportional to the difference between points. A stress score < 0.2 indicates that NMDS can accurately reflect the degree of difference between groups. Analysis of similarities (Anoism) is a non-parametric test used to test the significance of differences. An R-value > 0 indicates significant differences between groups, an R-value < 0 indicates that the differences within groups were greater than those between groups, and a *P*-value < 0.05 indicates statistical significance.
• Differential analysis of gut microbiota: (1) Differential relative abundance: We compared the differences in microbial distribution between groups according to the relative abundance of communities at different levels of phylum, class, order, family, genus, and species. In this study, we used the Metastat method to analyze microbial differences between the two groups at the phylum, family, and genus levels. (2) Differentially dominant microorganisms: We found microbial differences between groups using linear discriminant analysis effect size (LEfSe) analysis. The LEfSe calculation method not only has statistical significance, but also focuses on biological correlation by using linear discriminant analysis (LDA) to reduce the dimension and evaluate the impact of species with significant differences (i.e., LDA score). The default LDA score was 2, which can be increased according to the characteristics of the community distribution to obtain more accurate data.

#### Follow-up of study group

We followed up with the SGA infants until 6 months after birth and assessed their neurodevelopmental outcomes using the Age and Staging Questionnaire-3 (ASQ-3). The same neurodevelopmental professional evaluation doctor, proficient in ASQ-3 scoring criteria, conducted the evaluation. ASQ-3 mainly includes five parts, namely communication, gross motor, fine motor, problem-solving, and personal–social, with each part containing six specific assessment questions.

#### Statistical analysis

Statistical software (SPSS 25.0) was used to analyze the data. Measurement data consistent with a normal distribution are expressed as the mean ± standard deviation (*x* ± *s*). Student’s *t*-test was used to compare two groups, while analysis of variance (ANOVA) was used to compare three or more groups. The measurement data that were not in line with the normal distribution were expressed as median (IQR) or median (P25, P75). The Wilcoxon Mann–Whitney *U* test was used for the comparison between two groups, while the Kruskal–Wallis *H* test was used for the comparison of three groups and above. The enumeration data were expressed as the number of cases and percentages, and comparisons between groups were performed using the χ*^2^* test. If the total number n was < 40 or at least one actual frequency, *t* < 1, Fisher’s exact test method was applied. For the correlation analysis of two quantitative datasets, Pearson correlation analysis was adopted if it conformed to the bivariate normal distribution; otherwise, Spearman correlation analysis was adopted. Statistical significance was set at *P* < 0.05.

#### Ethical approval

The study was approved by the Ethics Committee of the Peking University First Hospital. The legal guardians of each participant provided written informed consent.

## Results

### Clinical characteristics of neonates in the small for gestational age and appropriate for gestational age groups

A total of 41 SGA neonates were enrolled in the SGA group, including 19 males (46.3%) and 24 neonates (58.5%) delivered *via* cesarean section. A total of 121 AGA neonates were enrolled in the AGA group, with 75 males (62.0%) and 33 neonates (27.5%) delivered *via* cesarean section. All neonates were born at a gestational age of 37–42 weeks and fed a mixed feed (breast milk + formula). The clinical characteristics of the enrolled neonates are presented in Table 1. A total of 31 neonates (75.6%) in the SGA group and 112 neonates (92.6%) in the AGA group had aspiration pneumonia or increased non-specific inflammatory indices. The proportion of ampicillin users in the AGA group was significantly higher than that in the SGA group. The gestational age and birth weight of the neonates in the SGA group were significantly lower than those in the AGA group, and the proportion of cesarean sections was significantly higher in the SGA group. The following clinical characteristics differed significantly between the SGA and AGA groups: infants from twin pregnancies, premature rupture of membranes, hospital stay of neonates, chorioamnionitis, and maternal antibiotics. There were no significant differences in sex, Apgar scores at 1 and 5 min, region, siblings, mother’s pregnancy weight gain, pregnancy complications (diabetes or gestational hypertension), or pet ownership between the two groups. None of the enrolled neonates were infected with the novel coronavirus, and neither their mothers nor their family members showed the emergence of pandemic-related mental or personality disturbances.

**TABLE 1:** Clinical characteristics of the SGA and AGA groups.

Descriptive variable	SGA group *n* = 41	AGA group *n* = 121	Statistic value	*P*
Male	19 (46.3%)	75 (62.0%)	3.076	0.079
Gestational age (weeks)	37.6 (1.3)	39.3 (1.6)	−4.946	< 0.001
Birthweight (grams)	2352.7 ± 300.6	3282.3 ± 331.7	15.66	< 0.001
Infants from twin pregnancy	10 (24.4%)	2 (1.7%)	19.887	< 0.001
Cesarean	24 (58.5%)	33 (27.5%)	12.872	< 0.001
Premature rupture of membranes	2 (4.9%)	36 (29.8%)	10.553	0.001
Apgar score at 1 min	10 (0)	10 (0)	0.428	0.669
Apgar score at 5 min	10 (0)	10 (0)	0.852	0.394
Mixed fed (formula + breast-feeding)	41 (100%)	121 (100%)	-	> 0.999
Ampicillin to neonates (first week)	31 (75.6%)	112 (92.6%)	6.942	0.008
Hospital stay of neonates (days)	7 (2)	6 (1)	−3.712	< 0.001
Sibling	14 (34.1%)	30 (24.8)	1.354	0.245
Mother’s age (years)	33.0 ± 3.6	32.2 ± 3.8	−0.343	0.732
Mother’s pregnancy weight gain (kg)	12.0 (4.8)	13.6 (5.0)	−1.273	0.203
Maternal smoking	0 (0%)	0 (0%)	-	> 0.999
Gestational hypertension	8 (19.5%)	10 (8.3%)	2.866	0.090
GDM or DM	10 (24.4%)	36 (29.8%)	0.433	0.511
Antenatal TG (mmol/L)	2.22 (1.82)	2.56 (1.67)	−0.286	0.775
Antenatal TCHO (mmol/L)	5.87 (2.38)	5.93 (2.40)	−0.485	0.628
Antenatal HDL (mmol/L)	1.63 (1.00)	1.69 (0.00)	−0.080	0.936
Antenatal LDL (mmol/L)	3.14 (1.40)	2.82 (1.54)	−1.115	0.265
Chorioamnionitis	11 (26.8%)	16 (13.2%)	4.082	0.043
Antibiotics to mother	8 (19.5%)	48 (39.7%)	5.501	0.019
Antenatal corticosteroids	0 (0%)	1 (0.8%)	-	> 0.999
Inclusion site-countryside	2 (4.9%)	6 (5.0%)	0.00	> 0.999

### Gut microbiota analysis of the small for gestational age and appropriate for gestational age groups

In this study, an average of 76,816 tags were measured per sample, and an average of 75,108 valid data points was obtained after quality control. The sequence was clustered into OTUs with 97% identity and 13,747 OTUs were obtained.

#### Sequencing depth and rationality

After obtaining all the OTUs, a rarefaction curve was drawn to evaluate whether the current sequencing depth of each sample could fully reflect the microbial diversity in the community samples. When the dilution curve tended to be flat (Supplementary Figure 1), the sequencing data gradually became reasonable. The coverage index of these samples fluctuated between 0.974 and 1, indicating that the sequencing depth was close to covering all bacterial communities in the tested samples.

#### Alpha and beta diversities

A comparison of the alpha diversity of fecal microbiota in the SGA group on different days in the first week of life revealed no statistical difference in the Chao1, ACE, Observed species, Simpson, and Shannon indices, indicating that there was no significant difference in the richness and diversity of gut microbiota within the SGA group at postnatal days 1, 3, 5, and 7 (Supplementary Table 1).

A comparison of alpha diversity of fecal microbiota between the SGA and AGA groups revealed that the SGA group’s gut microbial diversity was significantly lower than that of the AGA group in the Chao1, ACE, Observed species, Simpson, and Shannon indices on the first day (< 0.05). On days 3, 5, and 7, the Chao1, ACE, and Observed species indices of fecal microbiota of the SGA group remained significantly lower than the AGA group (*P* < 0.05), whereas there was no significant difference in the Simpson and Shannon indices (Supplementary Figure 2 and Supplementary Table 2).

**FIGURE 1:**
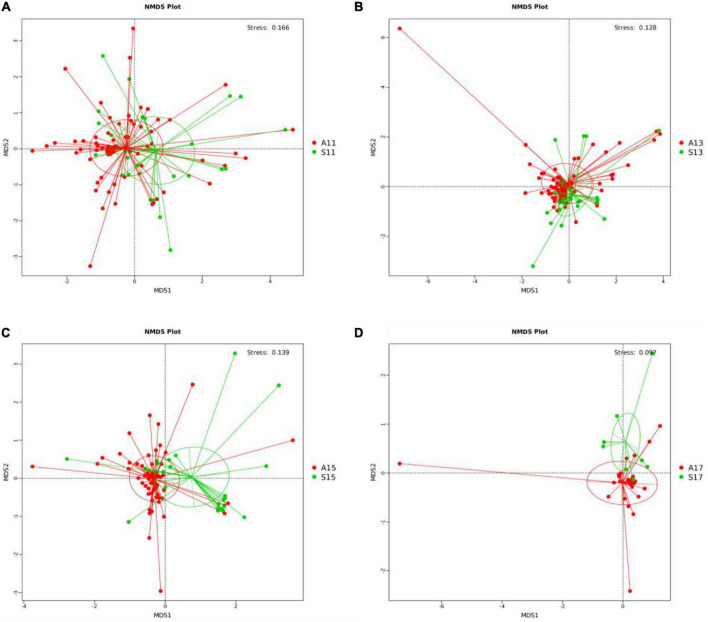
NMDS analysis between the SGA and AGA groups on days 1, 3, 5, and 7. **(A)** NMDS analysis on day 1. **(B)** NMDS analysis on day 3. **(C)** NMDS analysis on day 5. **(D)** NMDS analysis on day 7. NMDS, non-metric multi-dimensional scaling. The red points represent belonging to the AGA group, and the green points represent belonging to the SGA group. S11, gut microbiota of the SGA group on day 1; A11, gut microbiota of the AGA group on day 1; S13, gut microbiota of the SGA group on day 3; A13, gut microbiota of the AGA group on day 3; S15, gut microbiota of the SGA group on day 5; A15, gut microbiota of the AGA group on day 5; S17, gut microbiota of the SGA group on day 7; A17, gut microbiota of the AGA group on day 7.

PCoA showed significant differences in the gut microbiota on days 1, 3, 5, and 7 between the two groups. The R-value > 0, and statistical analysis between groups showed significant differences (*P* < 0.05) (Supplementary Figures 3A–H and Supplementary Table 3). The NMDS analysis (Supplementary Table 4) indicated that the gut microbiota of the two groups differed significantly on days 1, 3, 5, and 7 (stress score < 0.2) (Figure 1).

#### Analysis of differential gut microbiota

##### Differential relative abundance

The main microbiota in the AGA group at the phylum level were *Firmicutes*, *Proteobacteria*, *Actinobacteri*a, and *Bacteroidetes* (Supplementary Figures 4, 5); at the family level, *Enterococcaceae*, *Streptococcaceae*, *Enterococcaceae*, *Staphylococcaceae*, *Vibrionaceae* (Supplementary Figures 6, 7); and at the genus level, *Enterococcus*, *Streptococcus*, *Escherichia-Shigella*, *Staphylococcus*, and *Vibrio* (Supplementary Figures 8A, 9).

**FIGURE 2:**
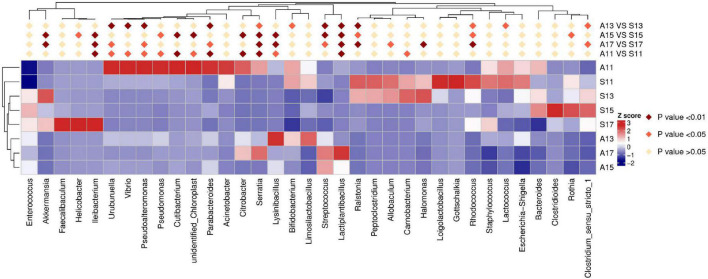
Results of heatmap analysis of species with significant differences between the SGA and AGA groups at the genus level. S11, gut microbiota of the SGA group on day 1; A11, gut microbiota of the AGA group on day 1; S13, gut microbiota of the SGA group on day 3; A13, gut microbiota of the AGA group on day 3; S15, gut microbiota of the SGA group on day 5; A15, gut microbiota of the AGA group on day 5; S17, gut microbiota of the SGA group on day 7; A17, gut microbiota of the AGA group on day 7.

On day 1, the SGA group showed a decreased relative abundance of *Cyanobacteria*, *Vibrionaceae*, *Parabacteroides*, *Lactiplantibacillus*, *Serratia*, *Citrobacter*, and *Cutibacterium*. However, the relative abundance of *Ileibacterium* was higher in the SGA group than in the AGA group (Table 2, Figure 2, and Supplementary Figures 10, 11).

**TABLE 2:** Gut microbiota analysis of the SGA and AGA groups on days 1, 3, 5, and 7 at levels of phylum, family, and genus.

Taxonomy	Days	Microbiota	SGA group	AGA group	*P*
Phylum (*P* < 0.05)	D1	*Cyanobacteria*	3.22 × 10^–4^	1.18 × 10^–2^	0.005
	D3	*Actinobacteria*	3.32 × 10^–2^	6.69 × 10^–2^	0.030
	D5	*Campylobacteria*	3.31 × 10^–4^	7.20 × 10^–6^	0.010
		*Verrucomicrobiota*	8.33 × 10^–4^	1.62 × 10^–5^	0.017
	D7	*Actinobacteria*	1.45 × 10^–2^	5.12 × 10^–2^	0.011
		*Cyanobacteria*	4.09 × 10^–5^	4.72 × 10^–3^	0.005
Family (*P* < 0.05)	D1	*Vibrionaceae*	4.95 × 10^–3^	4.37 × 10^–2^	0.021
	D3	*Streptococcaceae*	7.23 × 10^–2^	1.74 × 10^–1^	0.003
		*Burkholderiaceae*	8.85 × 10^–2^	7.22 × 10^–4^	0.002
		*Vibrionaceae*	7.40 × 10^–4^	6.88 × 10^–3^	0.003
	D5	*Streptococcaceae*	1.18 × 10^–1^	2.58 × 10^–1^	0.002
		*Burkholderiaceae*	9.37 × 10^–3^	4.69 × 10^–4^	0.022
		*Erysipelotrichaceae*	1.40 × 10^–3^	9.85 × 10^–5^	0.048
		*Micrococcaceae*	2.85 × 10^–2^	1.94 × 10^–3^	0.016
		*Helicobacteraceae*	3.22 × 10^–4^	5.85 × 10^–6^	0.016
	D7	*Streptococcaceae*	9.06 × 10^–2^	2.85 × 10^–1^	0.027
		*Burkholderiaceae*	1.29 × 10^–2^	7.39 × 10^–5^	0.003
		*Erysipelotrichaceae*	6.98 × 10^–2^	1.69 × 10^–4^	0.017
Genus (*P* < 0.01)	D1	*Parabacteroides*	5.46 × 10^–5^	1.02 × 10^–2^	0.001
		*Lactiplantibacillus*	3.15 × 10^–6^	4.84 × 10^–4^	0.001
		*Serratia*	2.31 × 10^–5^	1.17 × 10^–3^	0.005
		*Citrobacter*	2.10 × 10^–6^	1.29 × 10^–3^	0.008
		*Cutibacterium*	2.04 × 10^–4^	2.93 × 10^–3^	0.009
		*Ileibacterium*	1.04 × 10^–3^	5.75 × 10^–6^	0.008
	D3	*Streptococcus*	7.19 × 10^–2^	1.73 × 10^–1^	0.003
		*Vibrio*	7.40 × 10^–4^	6.88 × 10^–3^	0.003
		*Pseudoalteromonas*	1.74 × 10^–4^	1.50 × 10^–3^	0.004
		*Uruburuella*	3.28 × 10^–5^	2.60 × 10^–4^	0.004
		*Parabacteroides*	1.75 × 10^–4^	2.41 × 10^–3^	0.006
		*Lactiplantibacillus*	9.74 × 10^–6^	1.86 × 10^–4^	0.006
		*Ralstonia*	8.85 × 10^–2^	6.61 × 10^–4^	0.001
	D5	*Lysinibacillus*	0.00	2.52 × 10^–3^	0.001
		*Streptococcus*	1.18 × 10^–1^	2.58 × 10^–1^	0.002
		*Lactiplantibacillus*	4.36 × 10^–6^	1.68 × 10^–3^	0.002
		*Cutibacterium*	2.29 × 10^–5^	1.88 × 10^–4^	0.008
		*Serratia*	2.18 × 10^–6^	2.11 × 10^–5^	< 0.001
		*Citrobacter*	0.00	1.75 × 10^–5^	< 0.001
		*Ileibacterium*	9.00 × 10^–4^	1.35 × 10^–6^	0.001
	D7	*Lysinibacillus*	0.00	1.01 × 10^–3^	0.009
		*Lactiplantibacillus*	6.30 × 10^–6^	4.74 × 10^–3^	0.005
		*Serratia*	0.00	1.44 × 10^–3^	0.001
		*Ralstonia*	1.28 × 10^–2^	5.57 × 10^–5^	0.003
		*Ileibacterium*	6.45 × 10^–2^	1.01 × 10^–6^	0.002
		*Akkermansia*	2.09 × 10^–3^	8.10 × 10^–6^	0.001
		*Halomonas*	2.71 × 10^–4^	0.00	0.001
		*Rhodococcus*	5.29 × 10^–4^	1.01 × 10^–6^	0.002

On day 3, the SGA group showed decreased relative abundances of *Actinobacteria*, *Streptococcaceae*, *Vibrionaceae*, *Streptococcus*, *Vibrio*, *Pseudoalteromonas*, *Uruburuella*, *Parabacteroides*, and *Lactiplantibacillus*, whereas *Burkholderiaceae*, and *Ralstonia were* higher in the SGA group than in the AGA group (Table 2, Figure 2, and Supplementary Figures 10, 11).

On day 5, the SGA group showed decreased relative abundances of *Streptococcaceae*, *Lysinibacillus*, *Streptococcus*, *Lactiplantibacillus*, *Cutibacterium*, *Serratia* and *Citrobacter*, while those of *Campylobacteria*, *Verrucomicrobiota*, *Burkholderiaceae*, *Erysipelotrichaceae*, *Micrococcaceae*, *Helicobacteraceae*, *Ileibacterium*, and *Akkermansia* were higher in the SGA group than in the AGA group (Table 2, Figure 2, and Supplementary Figures 10, 11).

On day 7, the SGA group showed decreased relative abundances of *Actinobacteria*, *Cyanobacteria*, *Streptococcaceae*, *Lysinibacillus*, *Lactiplantibacillus*, and *Serratia*, whereas those of *Burkholderiaceae*, *Erysipelotrichaceae*, *Ralstonia*, *Ileibacterium*, *Akkermansia*, *Halomonas*, and *Rhodococcus* were higher in the SGA group than in the AGA group (Table 2, Figure 2, and Supplementary Figures 10, 11).

##### Differential dominant microorganisms

**FIGURE 3:**
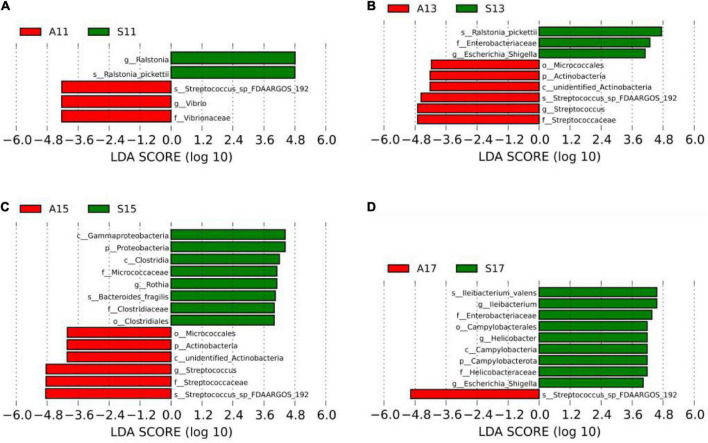
LEfSe comparison between the SGA and AGA groups. **(A)** LDA score histogram of differential microbiota of the two groups on day 1. **(B)** LDA score histogram of differential microbiota of the two groups on day 3. **(C)** LDA score histogram of differential microbiota of the two groups on day 5. **(D)** LDA score histogram of differential microbiota of the two groups on day 7. p_ represents phylum level, c _ represents class level, o_ represents order level, f_ represents family level, g_ represents genus level, and s_ represents species level. The length of the column represents the LDA score, and the greater the score, the greater the influence of the dominant microbiota. LDA, linear discriminant analysis; LEfSe, linear discriminant analysis effect size. S11, gut microbiota of the SGA group on day 1; A11, gut microbiota of the AGA group on day 1; S13, gut microbiota of the SGA group on day 3; A13, gut microbiota of the AGA group on day 3; S15, gut microbiota of the SGA group on day 5; A15, gut microbiota of the AGA group on day 5; S17, gut microbiota of the SGA group on day 7; A17, gut microbiota of the AGA group on day 7.

LEfSe was used to analyze differentially dominant microorganisms, and the LDA value was set to 4. On day 1, the dominant microorganisms in the SGA group were *g-Ralstonia* and *s-Ralstonia_pickettii*, while *s-Streptococcus_sp_FDAARGOS_192*, *f-Vibrionaceae*, and *g-Vibrio* were dominant in the AGA group. On day 3, the dominant microorganisms in the SGA group were *s-Ralstonia_pickettii* and *g-Escherichia_Shigella*, while *f-Streptococcaceae*, *g-Streptococcus*, and *s-Streptococcus_sp_FDAARGOS_192* were dominant in the AGA group. On day 5, the dominant microorganisms in the SGA group were *c-Clostridia*, *g-Rothia*, *s-Bacteroides_fragilis*, and *o-Clostridiales*, while *f-Streptococcaceae*, *g-Streptococcus*, and *s-Streptococcus_sp_FDAARGOS_192* were dominant in the AGA group. On day 7, the dominant microorganisms in the SGA group were *s-Ileibacterium_valens*, *g-Ileibacterium*, *f-Enterobacteriaceae*, *g-Helicobacter*, and *g-Escherichia_Shigella*, while *s-Streptococcus_sp_FDAARGOS_192* were dominant in the AGA group (Figure 3 and Supplementary Figures 12A–D).

### Correlation analysis between the top six microbiota at the genus and species levels in the small for gestational age group and ASQ-3 scores at 6 months of age

Neonates in the SGA group were followed up to 6 months of age, of which, 38 (92.7%) infants completed the follow-up and three infants were lost to follow-up (7.3%). The fine motor scores of ASQ-3 were negatively correlated with the relative abundance of *s-Bacteroides_fragilis* on day 1 (*r* = −0.412, *P* = 0.041). On day 7, the communication scores were positively correlated with the relative abundances of *g-Bacteroide*s (*r* = 0.875, *P* = 0.004) and *s-Bacteroides_fragilis* (*r* = 0.886, *P* = 0.003), whereas a negative correlation was observed between gross motor scores and the relative abundance of *s-Clostridium_saccharobutylicum* (*r* = −0.736, *P* = 0.037; Table 3).

**TABLE 3:** Correlation analysis between the top six microbiota at genus and species level in the SGA group and ASQ-3 scores at 6 months postnatal age.

Days and microbiota	ASQ-3 scores	Correlation index (*r*)	*P*
D7 *Bacteroides*	Communication	0.875	0.004
	Gross motor	0.012	0.977
	Fine motor	0.111	0.793
	Problem solving	0.101	0.811
	Personal–social	0.506	0.201
D7 *Bacteroides_fragilis*	Communication	0.886	0.003
	Gross motor	0.050	0.907
	Fine motor	0.050	0.906
	Problem solving	0.103	0.809
	Personal–social	0.050	0.207
D1 *Bacteroides_fragilis*	Communication	−0.055	0.794
	Gross motor	0.030	0.886
	Fine motor	−0.412	0.041
	Problem solving	−0.012	0.954
	Personal–social	−0.049	0.818
D7 *Clostridium_saccharobutylicum*	Communication	−0.230	0.583
	Gross motor	−0.736	0.037
	Fine motor	−0.221	0.599
	Problem solving	−0.053	0.900
	Personal–social	−0.299	0.472

### Analysis of gut microbiota of neonates in small for gestational age group with different neurological prognosis (communication)

We followed SGA infants until 6 months of age, and 38 of them completed the follow-up. A communication score ≥ 40 was considered normal. There were 31 (81.6%) infants with normal communication scores and 7 (18.4%) infants with poor communication scores in the SGA group. The two subgroups showed no significant differences in sex, gestational age, birth weight, mode of delivery, feeding pattern, or antibiotic application within 1 week after birth (Table 4).

**TABLE 4:** Clinical characteristics of the good and poor communication score groups in the SGA population.

Descriptive variable	Good communication score group	Poor communication score group	Statistic value	*P*
Male	13 (41.9%)	4 (57.1%)	-	0.678
Gestational age (weeks)	37.9 ± 1.1	39.2 ± 1.7	1.850	0.106
Birthweight (grams)	2325.7 ± 288.5	2519.3 ± 335.3	1.559	0.128
Birthweight percentile < P3	9 (29.0%)	3 (42.9%)	-	0.656
Cesarean	18 (58.1%)	3 (42.9%)	-	0.678
Ampicillin to neonates (first week)	23 (74.2%)	5 (71.4%)	-	> 0.999

#### Alpha and beta diversities

**FIGURE 4:**
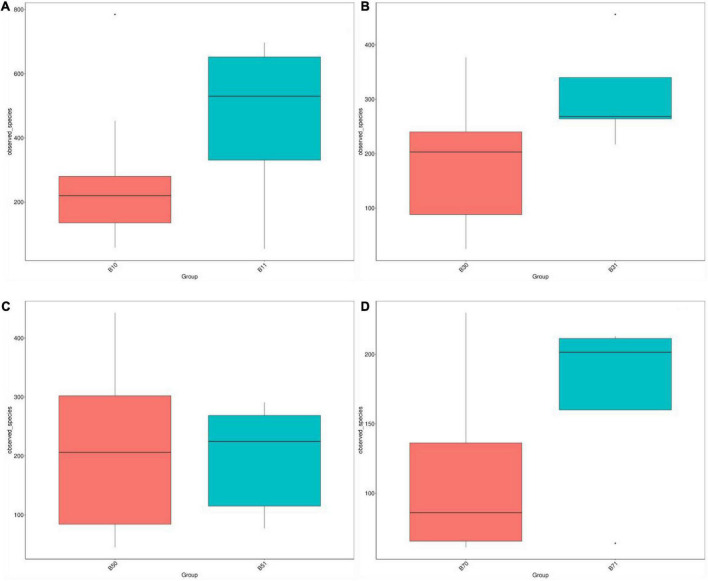
Distribution box plots of alpha diversity index between the good and poor communication score groups on days 1, 3, 5, and 7. **(A)** Distribution box plots of the alpha diversity index on day 1. **(B)** Distribution box plots of alpha diversity index on day 3. **(C)** Distribution box plots of alpha diversity index on day 5. **(D)** Distribution box plots of alpha diversity index on day 7. B11, gut microbiota of the poor communication score group on day 1; B10, gut microbiota of the good communication score group on day 1; B31, gut microbiota of the poor communication score group on day 3; B30, gut microbiota of the good communication score group on day 3; B51, gut microbiota of the poor communication score group on day 5; B50, gut microbiota of the good communication score group on day 5; B71, gut microbiota of the poor communication score group on day 7; B70, gut microbiota of the good communication score group on day 7.

A comparison of the alpha diversity of fecal microbiota between the good and poor communication score groups revealed no statistical difference in the Chao1, ACE, Observed species, Simpson, and Shannon indices on days 1, 5, and 7. On day 3, the gut microbial diversity of the poor communication score group was significantly higher than that of the good communication score group in the Chao1, ACE, and Observed species indices (*P* < 0.05), whereas there was no significant difference in the Simpson and Shannon indices (Figure 4 and Supplementary Table 5).

PCoA and Anosim showed no significant differences in the gut microbiota on days 1 and 7 between the good and poor communication score groups; although the R-value was > 0, but the statistical analysis between the groups showed no significant difference (*P* > 0.05). There were no significant differences in gut microbiota on days 3 and 5 in the good and poor communication score groups; the R-value was < 0, but the statistical analysis within groups showed no significant difference (*P* > 0.05) (Supplementary Figures 13A–D and Supplementary Table 6). However, NMDS analysis indicated that the gut microbiota of the two groups differed significantly on days 1, 3, 5, and 7 (stress score < 0.2) (Supplementary Figures 14A–D).

#### Analysis of differential gut microbiota

##### Differential relative abundance

The main microbiota in the good communication score group included *Firmicutes*, *Proteobacteria*, *Actinobacteri*a, and *Bacteroidetes* at the phylum level (Supplementary Figure 15); *Enterococcaceae*, *Burkholderiaceae*, *Enterobacteriaceae*, *Streptococcaceae*, and *Staphylococcaceae* at the family level (Supplementary Figure 16); *Enterococcus*, *Streptococcus*, *Escherichia-Shigella*, *Staphylococcus*, *Bacteroides*, and *Ileibacterium* at the genus level (Supplementary Figure 17); and *Ralstonia_pickettii*, *Streptococcus_sp_FDAARGOS_192*, *Bacteroides_fragilis*, *Ileibacterium_valen*, *Rothia_mucilaginosa*, and *Clostridium_saccharobutylicum* at the species level (Supplementary Figure 18). On day 1, the poor communication score group showed decreased relative abundances of *Enterobacteriaceae*, *Streptococcaceae*, and *Streptococcus* (Supplementary Table 7 and Supplementary Figures 19A–D). On day 3, the main microbiota between the good and poor communication score groups showed no significant differences in the phylum, family, genus, and species levels (Supplementary Table 7 and Supplementary Figures 20A–D). On day 5, the poor communication score group showed decreased relative abundances of *Staphylococcaceae* and *Staphylococcus*, whereas the relative abundance of *Enterococcus* was higher in the poor communication score group (Supplementary Table 7 and Supplementary Figures 21A–D). On day 7, the poor communication score group showed decreased relative abundances of *Bacteroidota*, *Bacteroides*, and *Bacteroides_fragilis*, whereas the relative abundance of *Corynebacterium* was higher in the poor communication score group (Supplementary Table 7 and Supplementary Figures 22A–D).

##### Differential dominant microorganisms

**FIGURE 5:**
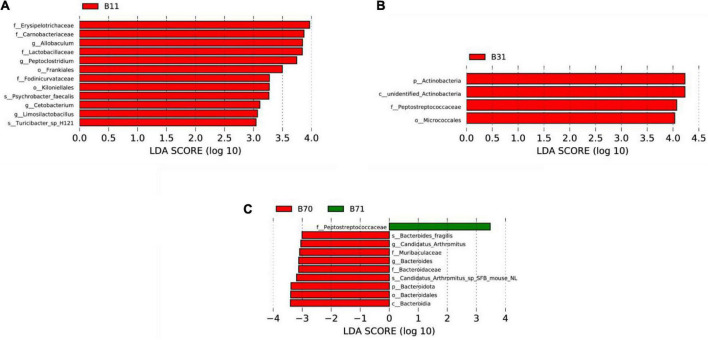
LEfSe comparison between the good and poor communication score groups. **(A)** LDA score histogram of differential microbiota of the two groups on day 1. **(B)** LDA score histogram of differential microbiota of the two groups on day 3. **(C)** LDA score histogram of differential microbiota of the two groups on day 7. p_ represents phylum level, c_ represents class level, o_ represents order level, f_ represents family level, g_ represents genus level, and s_ represents species level. B11, gut microbiota of the poor communication score group on day 1; B31, gut microbiota of the poor communication score group on day 3; B71, gut microbiota of the poor communication score group on day 7; B70, gut microbiota of the good communication score group on day 7.

On day 1, the dominant microorganisms in the poor communication score group were f*-Erysipelotrichaceae*, *f-Carnobacteriaceae*, and *g-Allobaculum*. On day 3, they were *p-Actinobacteria*, *c-unidentified Actinobacteria*, and *f-Peptostreptococcaceae*. There were no dominant microorganisms in the poor communication score group on day 5. On day 7, the dominant microorganism in the poor communication score group was *f-Peptostreptococcaceae*. *p-Bacteroidota*, *c-Bacteroidia*, *o-Bacteroidales*, *f-Bacteroidaceae*, *g-Bacteroides*, and *s_Bacteroides_fragilis* formed the dominant microorganisms in the good communication score group on day 7 (LDA score > 3). There were no dominant microorganisms in the good communication score group on days 1, 3, and 5 (Figure 5 and Supplementary Figure 23).

#### Analysis of differential microbiota and communication scores at 6 months of age

We analyzed the correlation between differentially abundant microbiota and communication scores at 6 months postnatal, and found that *p-Bacteroidota*, *g-Bacteroides*, and *s-Bacteroides_fragilis* were positively correlated with communication scores, on day 7. Moreover, there was no correlation between the rest of the differentially abundant microbiota and communication scores (Supplementary Table 8).

### Effect of delivery mode on gut microbiota of small for gestational age neonates and ASQ-3 scores

A total of 41 SGA neonates were enrolled, including 24 neonates (58.5%) delivered by cesarean section and 17 neonates (41.5%) through vaginal delivery. The two subgroups showed no significant differences in sex, gestational age, birth weight, feeding patterns, or antibiotic application within 1 week after birth (Supplementary Table 9).

#### Alpha and beta diversities

A comparison of alpha diversity of fecal microbiota between the cesarean birth and vaginal delivery groups revealed no statistical difference in the Chao1, ACE, Observed species, Simpson, and Shannon indices on days 1, 3, and 5 (Supplementary Table 10 and Supplementary Figure 24).

PCoA and Anosim showed no significant differences in gut microbiota between the cesarean birth and vaginal delivery groups; the R-value was > 0, but the statistical analysis between the groups showed no significant difference (*P* > 0.05; Supplementary Figures 25A–C, 26A–C and Supplementary Table 11). However, NMDS analysis indicated that the gut microbiota of the two groups differed significantly on days 1, 3, and 5 (stress score < 0.2; Supplementary Figures 27A–C).

#### Differential relative abundance and dominant microorganisms

Between the two groups, there were differences in the relative abundances of *Campylobacterota*, *Bacteroidota*, and *Actinobacteria* at the phylum level (Supplementary Figures 28A–C and Supplementary Table 12); *Bacteroidaceae* and *Tannerellaceae* at the family level (Supplementary Figures 29A–C and Supplementary Table 12); and *Bacteroides*, *Eubacterium_hallii_group*, and *Ileibacterium* at the genus level (Supplementary Figures 30A–C and Supplementary Table 12). On day 1, the dominant microorganisms in the vaginal delivery group were *g-Ileibacterium* and *s-Ileibacterium_valens* (Supplementary Figure 31A); *f-Bacteroidaceae*, *g-Bacteroides*, and *s-Bacteroides_fragilis* on day 3 (Supplementary Figure 31B); and *p-Bacteroidota*, *c-Bacteroidia*, *o-Bacteroidales*, *f-Bacteroidaceae*, *g-Bacteroides*, and *s-Bacteroides_fragilis* on day 5. The dominant microorganisms in the cesarean birth group were *o-Staphylococcales*, *f-Staphylococcaceae*, and *g-Staphylococcus* (Supplementary Figure 31C).

In summary, the gut microbiota with differences between the two delivery modes mainly included *g-Bacteroides*, *g-Staphylococcus*, and *g-Ileibacterium*, whereas the gut microbiota with differences between the SGA and AGA groups mainly included *g-Escherichia_Shigella*, *g-Ralstonia*, *g-Clostridium*, and *g-Streptococcus.*

#### Effect of delivery mode on ASQ-3 scores of small for gestational age infants at 6 months of age

The ASQ-3 scores of SGA neonates at 6 months of age were not associated with the delivery mode (Supplementary Table 13).

### Effect of antibiotic application on gut microbiota of small for gestational age neonates and ASQ-3 scores

A total of 41 SGA neonates were enrolled, including 31 neonates (75.6%) treated with antibiotics and 10 neonates (24.4%) treated without antibiotics, according to their condition. The two subgroups showed no significant differences in sex, gestational age, birth weight, feeding patterns, or delivery mode (Supplementary Table 14).

#### Alpha and beta diversities

A comparison of the alpha diversity of fecal microbiota between the group with antibiotics and the group without antibiotics revealed no statistical difference in the Chao1, ACE, Observed species, Simpson, and Shannon indices on days 1, 3, and 5 (Supplementary Table 15 and Supplementary Figure 32).

The R- and *P*-values were > 0.05 on days 1 and 5, indicating that there was no statistical difference between the two groups on days 1 and 5. On day 3, R-value < 0 and *P* > 0.05 3, indicating that there was no statistical difference between the two groups (Supplementary Figures 33A–C, 34A–C and Supplementary Table 16). However, NMDS analysis indicated that the gut microbiota of the two groups differed significantly on days 1, 3, and 5 (stress score < 0.2; Supplementary Figures 35A–C).

#### Differential relative abundance and dominant microorganisms

Between the two groups, there was a difference in the relative abundance of *Gemmatimonadota* at the phylum level (Supplementary Figures 36A–C and Supplementary Table 17); *Carnobacteriaceae*, *Enterococcaceae*, *Enterobacteriaceae*, and *Burkholderiaceae* at the family level (Supplementary Figures 37A–C and Supplementary Table 17); *Aminobacter*, *Georgenia*, and *Loigolactobacillus* at the genus level (Supplementary Figures 38A–C and Supplementary Table 17). On day 1, the dominant microorganisms in the antibiotic-treated group were *f-Micrococcaceae*, *g-Rothia*, *o-Micrococcales*, and *s-Rothia_mucilaginosa* (Supplementary Figure 39A); *s-Clostridium_saccharobutylicum*, *g-Staphylococcus*, *o-Staphylococcales*, *f-Staphylococcaceae*, *s-Clostridium_perfringens*, *s-Enterococcus_faecalis*, *s-Clostridioides_difficile*, and *g-Clostridioides* on day 3 (Supplementary Figure 39B); and *o-Lactobacillales*, *f-Enterococcaceae*, *g-Enterococcus*, and *c-Bacilli* on day 5. The dominant microorganisms in the group without antibiotics were *o-Enterobacterales*, *f-Enterobacteriaceae*, *g-Escherichia_Shigella*, *g-Clostridium_sensu_stricto_1*, *f-Peptostreptococcaceae*, *g-Clostridioides*, and *s-Clostridioides_difficile* (Supplementary Figure 39C).

In summary, the gut microbiota with differences between the two groups with different applications of antibiotics mainly included *g-Rothia*, *g-Clostridioides*, *g-Staphylococcus, g-Enterococcus*, *g-Escherichia_Shigella*, and *g-Clostridium*; while the gut microbiota with differences between the SGA and AGA groups mainly included *g-Escherichia_Shigella*, *g-Ralstonia*, *g-Clostridium*, and *g-Streptococcus.*

#### Effect of application of antibiotics on ASQ-3 scores of small for gestational age infants at 6 months of age

The ASQ-3 scores of SGA neonates at 6 months of age were not associated with the use of antibiotics (Supplementary Table 18).

## Discussion

SGA infants exhibit more significant long-term health issues, including a variety of major and subtle neurodevelopmental delays, than their appropriate gestational age counterparts (Sharma et al., 2016; McCowan et al., 2018; Kesavan and Devaskar, 2019). At present, the mechanisms underlying neurodevelopmental delay in SGA infants are unclear. A growing number of studies have indicated that the gut microbiota plays an important role in early neural development (Carlson et al., 2018; Cohen Kadosh et al., 2021; Seki et al., 2021). Colonization and maturation of the gut microbiota overlap with the critical period of early brain development, and an imbalance in gut microbiota during the early postnatal period may disrupt the developmental programming of the brain through the gut−brain axis, leading to brain injury and long-term neurodysplasia later in life (Al-Asmakh et al., 2012; Cryan et al., 2019; Seki et al., 2021). Therefore, it is of great significance to study the association between gut microbiota and neural development in SGA infants and to explore the impact of specific microbiota on neural development.

In our study, the alpha diversity of gut microbiota in the SGA group was significantly lower than that in the AGA group on days 1, 3, 5, and 7, consistent with the findings of a previous study (Zhang et al., 2019). At the phylum level, the main microbiota of the SGA group were *Firmicutes*, *Proteobacteria*, *Actinobacteri*a, and *Bacteroidota*, similar to the results of previous studies (Martí et al., 2021; Zhang et al., 2021). With respect to the differential abundance of gut microbiota between the SGA and AGA groups, *Actinobacteria* (3, 7 days) and *Cyanobacteria* (1, 7 days) were significantly lower in the SGA group. Furthermore, another study found that pigs with intrauterine growth restriction had a lower relative abundance of *Actinobacteria* (Che et al., 2019), consistent with observations of our study. *Cyanobacteria* have been reported to exhibit good anti-inflammatory, antioxidant, cholesterol-lowering, and antimicrobial activities (Ferrazzano et al., 2020). *Campylobacterota*, considered to be associated with intestinal and extraintestinal infections (Fitzgerald, 2015; Same and Tamma, 2018), were more abundant on day 5 and were the dominant microbiota on day 7 in the SGA group.

At the genus level, the main microbiota of the SGA group included *Enterococcus*, *Ralstonia*, *Staphylococcus*, *Streptococcus*, *Escherichia-Shigella*, and *Bacteroides*, consistent with findings of previous research (Bäckhed et al., 2015; Gabriel et al., 2018; Zhang et al., 2021). Among the differential microbiota between the SGA and AGA groups, *Ralstonia* and *Ralstonia_pickettii* were the dominant microorganisms in the SGA group on day 1, and *Ralstonia* was higher in the SGA group on days 3, 5, and 7. Reports have shown that *Ralstonia* is related to the pathogenesis of nervous system diseases such as autism spectrum disorder (ASD) and Parkinson’s disease (PD), and its mechanism may involve neuroinflammation and immune activation (Keshavarzian et al., 2015; Ragusa et al., 2020). *F-Enterobacteriaceae* (7 days), *g-Escherichia_Shigella* (3, 7 days), and *g-Helicobacter* (7 days) were the dominant microorganisms in the SGA group. *Escherichia_Shigella* is well-known as a pathogenic enterobacterium; some strains of *Helicobacter* are recognized as important pathogens in gastrointestinal diseases, such as peptic ulcers and gastric cancer, and some strains are associated with bacteremia in immunocompromised and immunocompetent human hosts (On et al., 2002; Boltin et al., 2019). *c-Clostridia*, *o-Clostridiales*, and *g-Rothia* were the dominant microorganisms in the SGA group on day 5, and *Clostridium_sensu_stricto_1* was higher in the SGA group on days 3 and 7, consistent with a previous study on the gut microbiota of neonates with asphyxia (Zhang et al., 2021). Researchers have found that the predominance of *o-Clostridiales* in infants is associated with poorer communication performance at 3 years of age (Sordillo et al., 2019). *Clostridium_sensu_stricto_1* is associated with a variety of inflammatory genes and is considered an opportunistic pathogen associated with intestinal inflammation (Wang et al., 2018; Li et al., 2019; Wen et al., 2021). Studies have found that *Rothia* is an opportunistic pathogen associated with various infections in humans, and most reported *Rothia* infections have occurred in patients with pneumonia, endocarditis, peritonitis, and septicemia (Fatahi-Bafghi, 2021). *Burkholderiaceae* was higher in the SGA group on days 3, 5, and 7. An increased relative abundance of *Burkholderiaceae* was observed in the gut of rats with cognitive impairment (Rong et al., 2021), and *Burkholderiaceae* was elevated in the brain tissue of patients with Alzheimer’s disease (AD) (Alonso et al., 2018). The SGA group showed decreased relative abundances of *Lactiplantibacillus* (1, 3, 5, and 7 days), *Streptococcus* (3, 5, 7 days), *Bifidobacterium*, and *Lactococcus* (3 days). Some strains of these four genera are considered probiotics (Guo et al., 2019; Prete et al., 2020; Wright et al., 2020; Griffin et al., 2022) because they improve memory impairment and cognitive function by reducing inflammation and oxidative stress (Den et al., 2020; Sivamaruthi et al., 2020; Griffin et al., 2022). *Ileibacterium* was higher in the SGA group on days 1, 5, and 7, and it was the dominant microbiota on day 7. *Ileibacterium* is a gram-positive anaerobic bacterium related to metabolic health that can decompose polysaccharides (Wang et al., 2021). An increased relative abundance of *Ileibacterium* has been observed in animal models of intestinal microecological disorders (Xiao et al., 2021; Wu et al., 2022). *Akkermansia* was higher in the SGA group on days 5 and 7. *Akkermansia* is the predominant member of *Verrucomicrobiota* (Wagner and Horn, 2006), with elevated levels in patients with multiple sclerosis (MS) linked to lower disability, and *Akkermansia* isolated from these patients ameliorates experimental autoimmune encephalomyelitis (EAE), suggesting that increased *Akkermansia* in MS may be a compensatory beneficial response (Cox et al., 2021).

In summary, large differences were observed in the gut microbiota between the SGA and AGA groups in the first week of life, which can be summarized as follows: (1) compared with AGA infants, the alpha diversity of term SGA infants was significantly lower; (2) certain pathogenic and conditional pathogenic bacteria increased or formed the dominant microorganisms in SGA infants, such as *Escherichia_Shigella*, *Ralstonia*, and *Clostridium*.

After a 6 month follow-up, we found an association between higher alpha diversity on day 3 and communication problems in SGA infants. This finding was surprising because a more mature gut microbial community in infancy usually has a high level of biological diversity (Carlson et al., 2018; Coker et al., 2021), and decreased microbial diversity is related to adverse health outcomes in adults (Rinninella et al., 2019; Nikolova et al., 2021). However, the relationship between microbial diversity and health conditions in children is mixed (Abrahamsson et al., 2014; Kostic et al., 2015; Aatsinki et al., 2019). Our findings are consistent with recent evidence that increased microbial diversity in infancy is not necessarily beneficial for subsequent neurocognitive outcomes (Carlson et al., 2018; Gao et al., 2019; Loughman et al., 2020). One study showed that a higher alpha diversity of the gut microbiota in 1-year-old children was associated with lower overall composite, visual reception, and expressive language scores at the age of 2 years (Carlson et al., 2018). Their team found a correlation between higher levels of microbiome diversity in infancy and weaker thalamus-amygdala connectivity a year later (Gao et al., 2019), while the amygdala regulates emotion and controls learning and memory (McDonald and Mott, 2017). The same study also found positive associations between alpha diversity and functional connectivity between the supplementary motor area (SMA) and the inferior parietal lobule (IPL), while SMA-IPL connectivity at 1 year of age was negatively correlated with cognitive outcomes at 2 years of age (Gao et al., 2019).

With respect to the differential gut microbiota of SGA infants with different neurodevelopmental outcomes at 6 months, *g-Allobaculum* was the dominant microbiota in the SGA group with a poor communication score on day 1. *Allobaculum* may provide good anti-inflammatory functions by producing free long-chain fatty acids and short-chain fatty acids (SCFAs) which are related to glucose and lipid metabolism (Wu et al., 2020; Pujo et al., 2021). Therefore, we assumed that *Allobaculum*-dominant gut microbiome may be a compensatory beneficial response for SGA infants with poor communication scores. We found that *p-Bacteroidota*, *g-Bacteroides*, and *s_Bacteroides_fragilis* increased in abundance and were the dominant microorganisms in the good communication score group on day 7. In addition, we observed correlations between increased abundances of *p-Bacteroidota*, *g-Bacteroides*, and *s-Bacteroides_fragilis* on day 7 and improved communication scores at 6 months, consistent with findings of previous studies on the association between gut microbiota in late infancy and subsequent neurodevelopment (Carlson et al., 2018; Tamana et al., 2021). We found that the relative abundance of *s-Bacteroides_fragilis* on day 1 was negatively correlated with the fine motor scores, similar to observation of a previous study on the association between *Bacteroides*-dominant gut microbiota at 3–6 months and subsequent delayed fine motor skills (Sordillo et al., 2019). *Bacteroides_fragilis* is a gram-negative obligate anaerobe with two subtypes. Enterotoxigenic *B. fragilis* (ETBF), identified as a common opportunistic pathogen in clinical infections, mainly causes colitis and systemic inflammation with the stimulation of toxins or lipopolysaccharides. The second subtype, non-toxigenic *B. fragilis* (NTBF), has been suggested as a potential probiotic in recent studies because of its ability to produce immunomodulatory substances such as polysaccharide A (PSA) and SCFAs (Sun et al., 2019; Qu et al., 2022). Non-toxigenic *B. fragilis* was found to be capable of protecting mice from central nervous system demyelination; this protective mechanism depended on the production of IL-10 (Ochoa-Repáraz et al., 2010). In addition, it was found to improve communication, stereotyped movement, anxiety-like behavior, and sensorimotor behavior in ASD model mice, as well as reduce the increased expression of IL-6 (Hsiao et al., 2013). Decreased levels of intestinal *Bacteroides* are also characteristic of children diagnosed with ASD (Dan et al., 2020; Iglesias-Vázquez et al., 2020). *Clostridium_saccharobutylicum* has a strong ability to produce butyrate (Huang et al., 2018; Miguel et al., 2019), and butyrate, as a microbial metabolite, can indirectly affect host metabolism through the gut–brain axis (Stilling et al., 2016). It has been reported that butyrate can reduce the inflammatory response of microglia and the hippocampus, inhibit inflammatory activities, promote the production of BDNF, and repair injured nerves (Kundu et al., 2019). What puzzled us was the negative correlation between the relative abundance of *s-Clostridium_saccharobutylicum* on day 7 and the gross motor scores at 6 months postnatal. This finding was unexpected and it is unclear why there was such a connection. Thus, we believe further investigation is required to confirm these results.

Briefly, gut microbial characteristics associated with neurological prognosis in SGA infants can be summarized as follows: (1) higher alpha diversity on day 3 was associated with poor communication performance in SGA infants at 6 months of age; (2) *Bacteroidota*, *Bacteroides*, *Bacteroides_fragilis*, and *Clostridium_saccharobutylicum* may be related to the neurodevelopmental outcomes of SGA infants at 6 months of age.

The development of neonatal gut microbiota is affected by several factors (Bäckhed et al., 2015; Rutayisire et al., 2016; Kapourchali and Cresci, 2020; Vandenplas et al., 2020). Most early colonists in the gut of neonates are maternal, and the mode of delivery strongly affects the formation of the early gut microbiota in term infants (Bäckhed et al., 2015; Rutayisire et al., 2016; Kapourchali and Cresci, 2020; Coelho et al., 2021). Studies have shown that vaginally-delivered neonates have more abundant *Bacteroides*, *Bifidobacterium*, and *Lactobacillus* (Bäckhed et al., 2015; Rutayisire et al., 2016; Coelho et al., 2021), whereas neonates delivered by cesarean section (CS) are enriched in *Staphylococcus*, *Streptococcus*, and *Clostridium* (Coelho et al., 2021). In our study, there was no difference in the alpha diversity of SGA neonates born by vaginal delivery and CS on days 1, 3, and 5, while the alpha diversity of SGA neonates delivered by CS showed a decreasing trend on day 7. Some researchers also found lower microbial diversity in the gut of infants delivered by CS than in vaginally-delivered infants in the first week of life (Bäckhed et al., 2015; MacIntyre et al., 2015; Rutayisire et al., 2016; Shi et al., 2018). LEfSe showed that the dominant microorganisms in vaginally-delivered SGA neonates were *g-Bacteroides* and *g-Ileibacterium*. *Bacteroides* seem to increase in abundance in vaginally-delivered infants compared with CS-delivered infants (Gronlund et al., 1999; Kabeerdoss et al., 2013; Hesla et al., 2014; Jakobsson et al., 2014; Rutayisire et al., 2016; Coelho et al., 2021). In addition, *Bifidobacterium* and *Lactobacillus* found in the vaginal delivery group did not increase significantly, in contrast to other findings (Bäckhed et al., 2015; Rutayisire et al., 2016; Coelho et al., 2021). The dominant microbiota of the cesarean birth group was *g-Staphylococcus* on day 5, similar to findings of previous studies (Li et al., 2018; Wampach et al., 2018; Coelho et al., 2021). However, *Streptococcus* and *Clostridium* found in the cesarean birth group did not increase significantly, in contrast to previous findings (Coelho et al., 2021). Therefore, SGA and delivery mode may have different effects on the development of gut microbiota in term neonates.

Antibiotic therapy can greatly alter the diversity and composition of neonatal gut microbiota (Nobel et al., 2015; Gasparrini et al., 2016). Associations between antibiotic therapy and decreased microbial diversity, increased abundance of *Firmicutes*, and decreased abundance of *Bacteroides* and *Bifidobacterium* have been reported (Gasparrini et al., 2016; Ficara et al., 2020). In our study, despite no statistical difference between the alpha diversity of the group with antibiotics and the group without antibiotics, the alpha diversity of SGA neonates treated with antibiotics was lower in the first week of life, similar to previous findings (Gasparrini et al., 2016; Ficara et al., 2020). Hence, we assumed that the therapeutic duration of antibiotics was insufficient to significantly disrupt gut microbial diversity. We also found that the composition of neonatal gut microbiota was altered by antibiotic therapy. However, there was no difference in the relative abundance of *Firmicutes*, *Bifidobacterium*, and *Bacteroides* between the two groups, which differed from previous findings (Ficara et al., 2020).

This study is the first to analyze the characteristics and evolution of the gut microbiota of term SGA infants in the first week of life, and also the first to explore the potential association between specific microbiota and neural development in SGA infants. Alongside the novelty of this study, it is worth mentioning the following limitations. (1) This was a single-center study; there may be differences in the gut microbiota of SGA infants from other hospitals. The results need to be further confirmed by multicenter and large-exponent investigations. (2) We only studied the gut microbiota of term SGA infants within 1 week after birth and the neurological prognosis at 6 months of age; we will follow up with SGA infants and discuss the relationship between neonatal gut microbiota and neurological development by comprehensively considering various factors, such as education mode and society. (3) The ASQ-3 is a screening scale, and because of the prevalence of the novel coronavirus, it was difficult to use diagnostic scales such as the Bayley scale to evaluate the prognosis of neurodevelopment.

## Conclusion

Compared to AGA infants, the gut microbial diversity of term SGA infants was significantly lower in the first week of life. Certain pathogenic and conditional pathogenic bacteria increased or formed the dominant microbiota in SGA infants, such as *Escherichia_Shigella*, *Ralstonia*, and *Clostridium*. This study suggests that there may be associations between alpha diversity, certain gut microbiota, and neurodevelopmental outcomes in SGA infants. The results showed that higher alpha diversity on day 3 was associated with poor communication performance in SGA infants at 6 months of age, and the gut microbiota factors affecting the prognosis of SGA infants included *Bacteroidota*, *Bacteroides*, *Bacteroides_fragilis*, and *Clostridium_saccharobutylicum.* SGA infants are at a higher risk for adverse neurodevelopmental outcomes; however, current methods for the clinical treatment of neurodevelopmental delay in SGA infants are limited. With further studies of the gut microbiota of SGA infants, we hope to provide further insights into the early treatment of SGA infants.

## Data availability statement

The datasets presented in this study can be found in online repositories. The names of the repository/repositories and accession number(s) can be found in the article/Supplementary material.

## Ethics statement

The studies involving human participants were reviewed and approved by the Ethics Committee of Peking University First Hospital. Written informed consent to participate in this study was provided by the participants’ legal guardian/next of kin.

## Author contributions

All authors listed have made great contributions to this work and have read and agreed to the published version of the manuscript.
