# Supplementary material for: Characteristics of gut microbiota of term small gestational age infants within 1 week and their relationship with neurodevelopment at 6 months
Source: Front Microbiol. 2022 Aug 24;13:912968. doi: 10.3389/fmicb.2022.912968 (PMC9449527; doi:10.3389/fmicb.2022.912968)
Supplement: Supplementary file 1 [file Data_Sheet_1.docx]

Supplementary Material

**Supplementary Figures**

(A) (B)


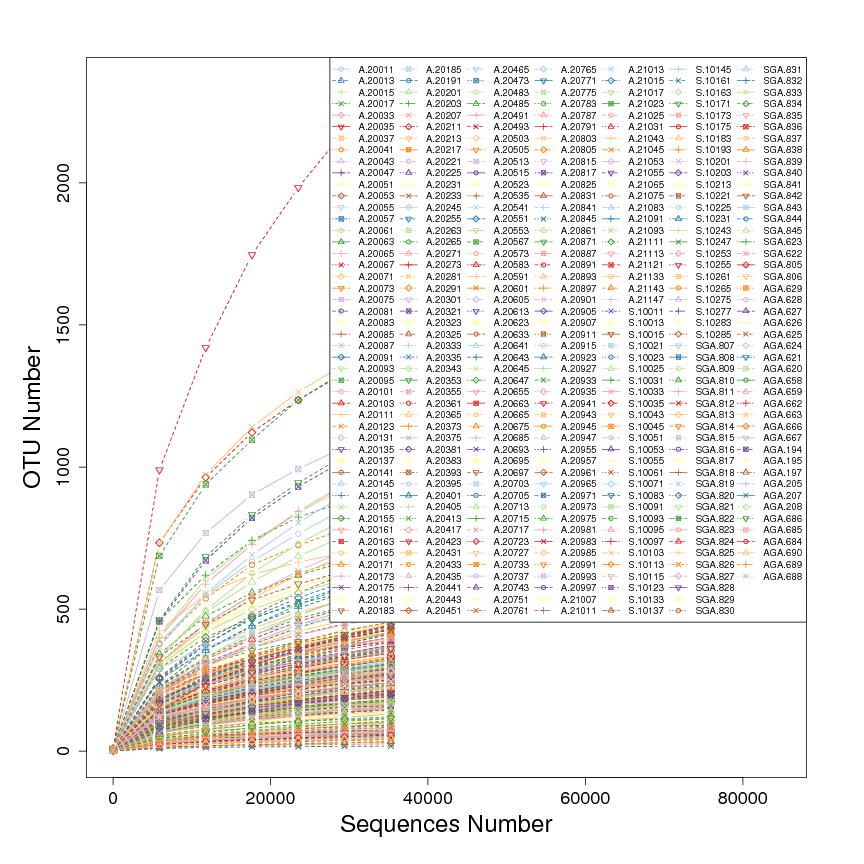

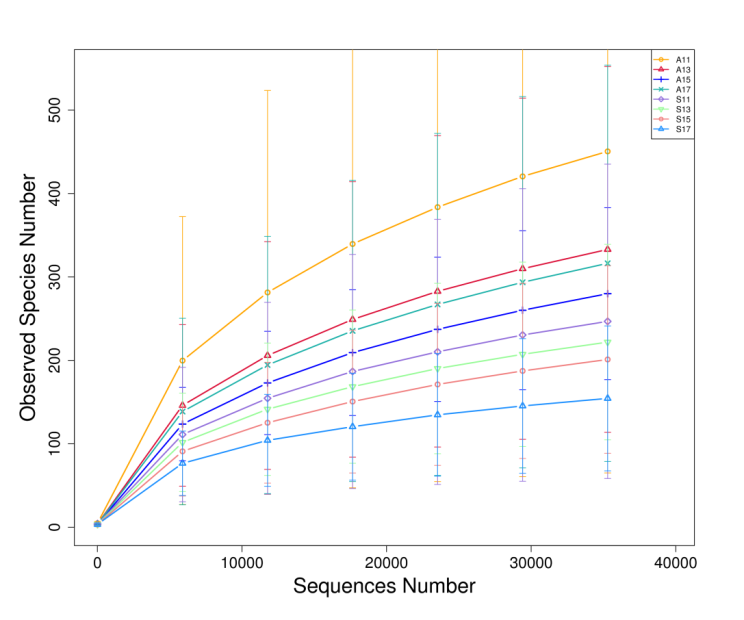


**Supplementary Figure 1.** (A) The rarefaction curve in samples. (B) The rarefaction curve in groups. S11, gut microbiota of the SGA group on day 1; A11, gut microbiota of the AGA group on day 1; S13, gut microbiota of the SGA group on day 3; A13, gut microbiota of the AGA group on day 3; S15, gut microbiota of the SGA group on day 5; A15, gut microbiota of the AGA group on day 5; S17, gut microbiota of the SGA group on day 7; A17, gut microbiota of the AGA group on day 7.


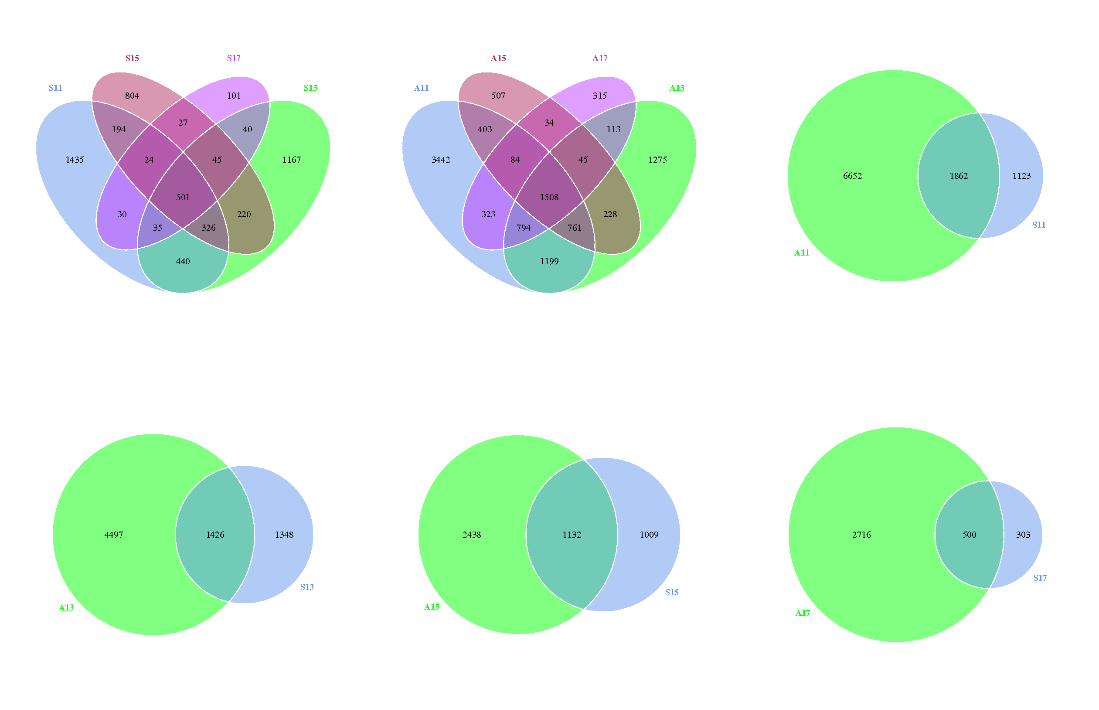


Supplementary Figure 2. Venn diagram of the two groups (SGA and AGA) on days 1, 3, 5, and 7. S11, gut microbiota of the SGA group on day 1; A11, gut microbiota of the AGA group on day 1; S13, gut microbiota of the SGA group on day 3; A13, gut microbiota of the AGA group on day 3; S15, gut microbiota of the SGA group on day 5; A15, gut microbiota of the AGA group on day 5; S17, gut microbiota of the SGA group on day 7; A17, gut microbiota of the AGA group on day 7.

(A) (B)

**
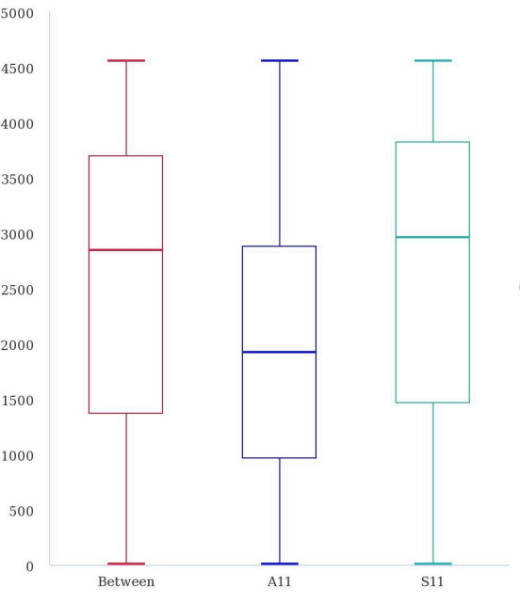

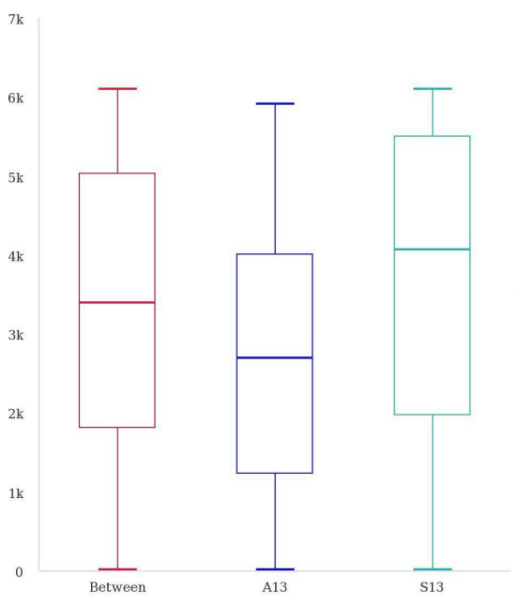
**

(C) (D)

**
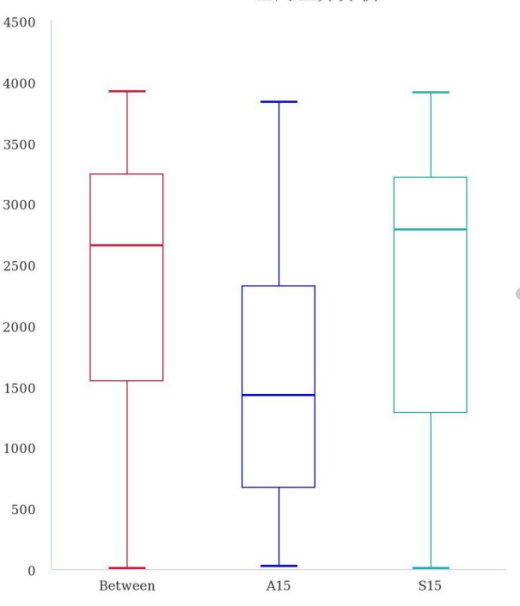

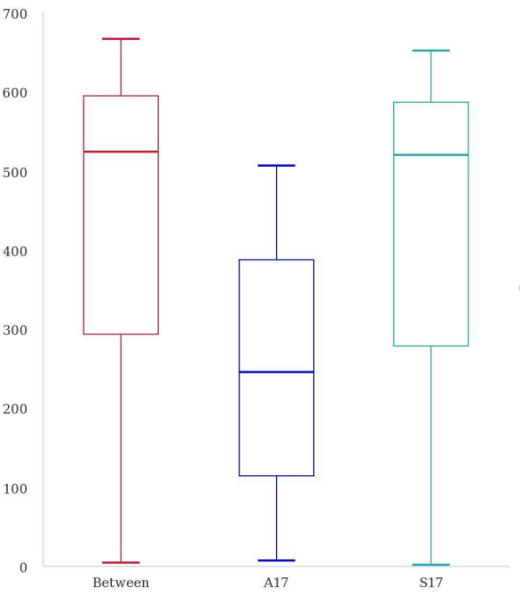
**

(E) (F)

**
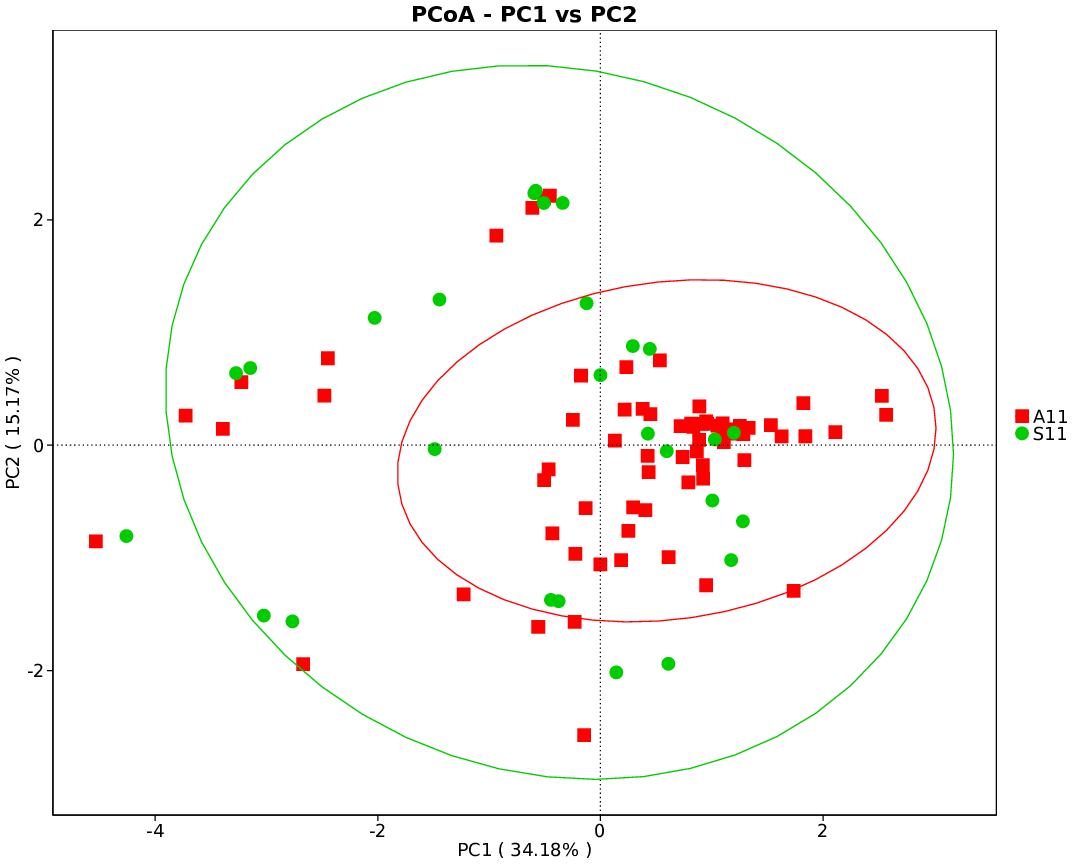

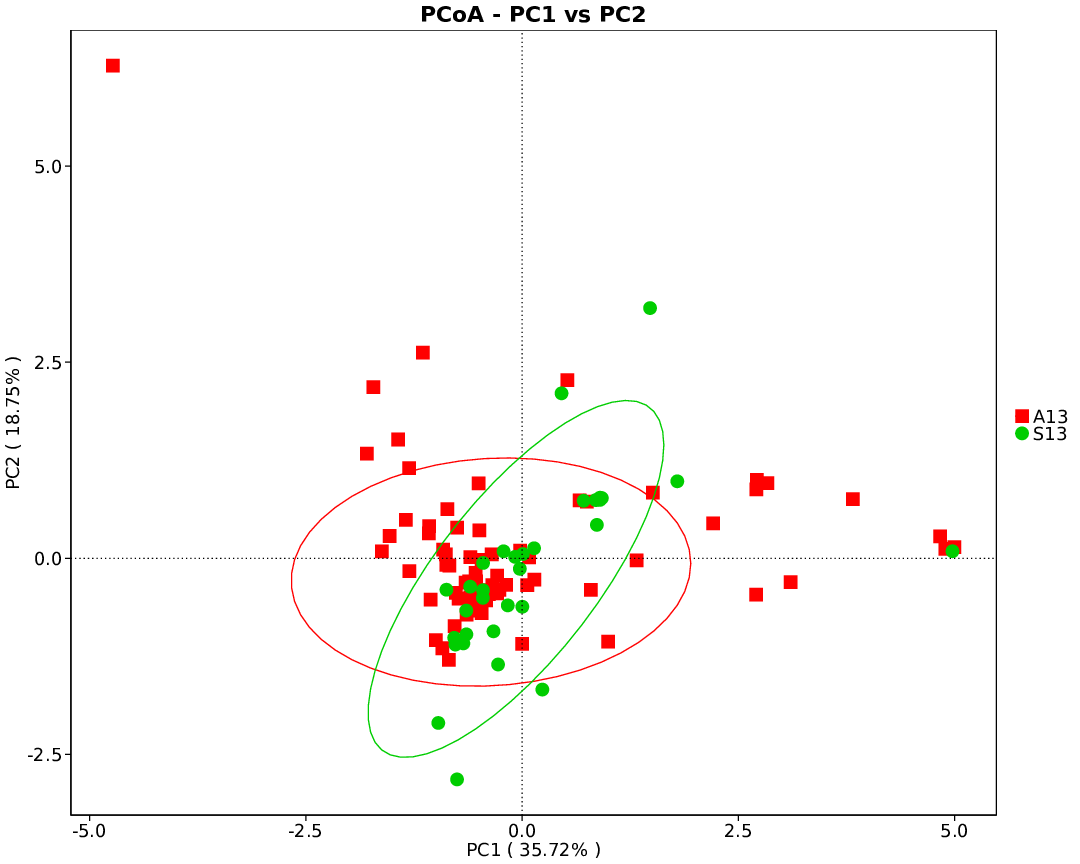
**

(G) (H)


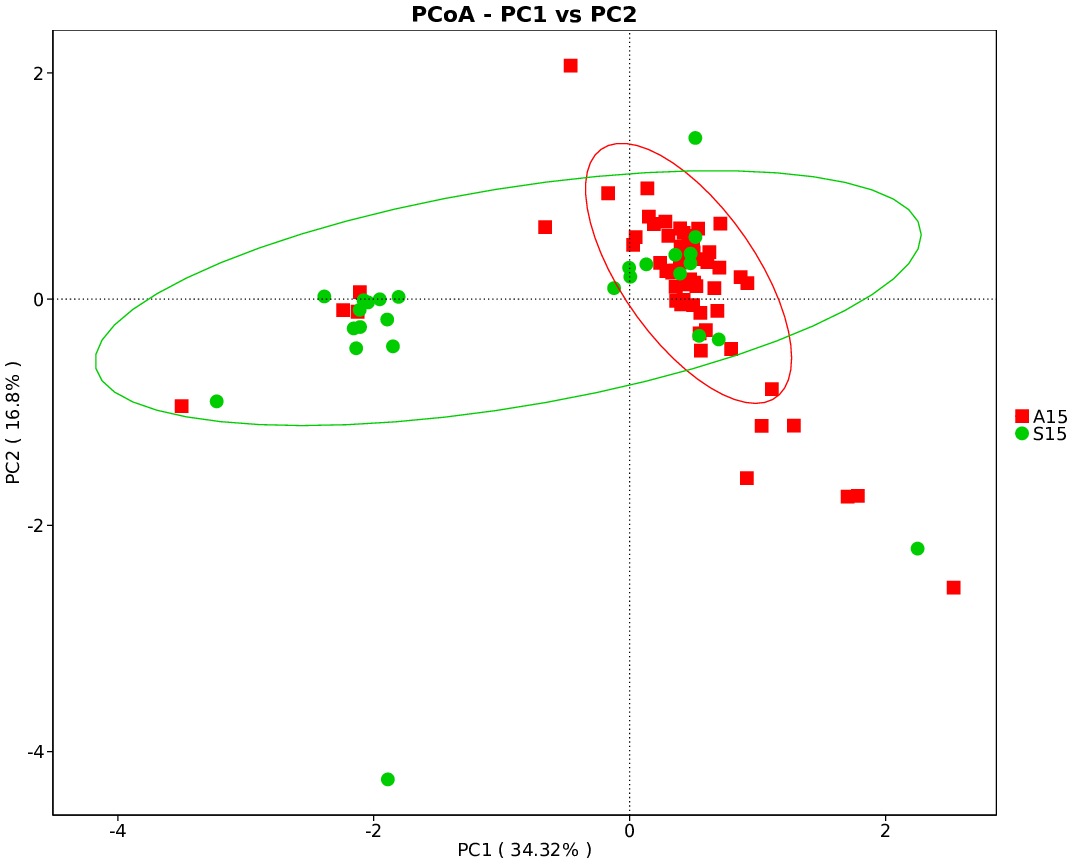

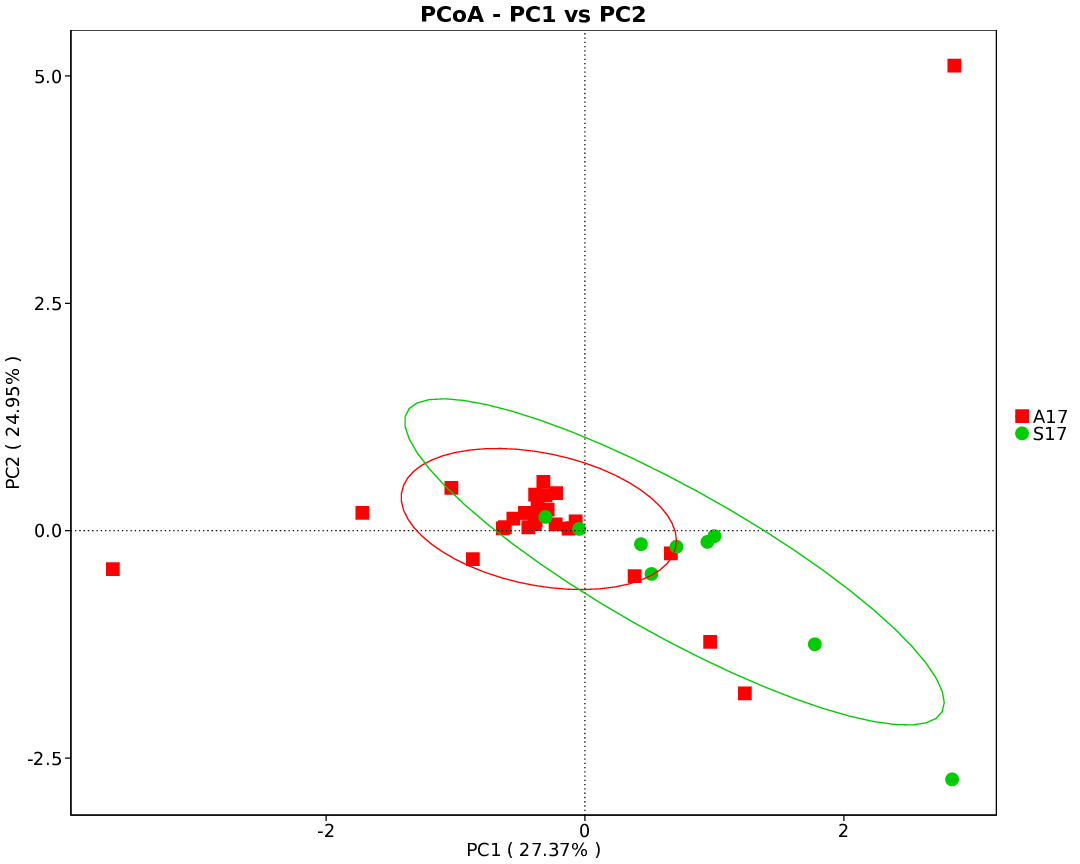


**Supplementary Figure 3.** Microbial differences and PCoA analysis between the SGA and AGA groups on days 1, 3, 5, and 7. (A) Microbial differences on day 1. (B) Microbial differences on day 3. (C) Microbial differences on day 5. (D) Microbial differences on day 7. (E) PCoA analysis on day 1. (F) PCoA analysis on day 3. (G) PCoA analysis on day 5. (H) PCoA analysis on day 7. S11, gut microbiota of the SGA group on day 1; A11, gut microbiota of the AGA group on day 1; S13, gut microbiota of the SGA group on day 3; A13, gut microbiota of the AGA group on day 3; S15, gut microbiota of the SGA group on day 5; A15, gut microbiota of the AGA group on day 5; S17, gut microbiota of the SGA group on day 7; A17, gut microbiota of the AGA group on day 7.


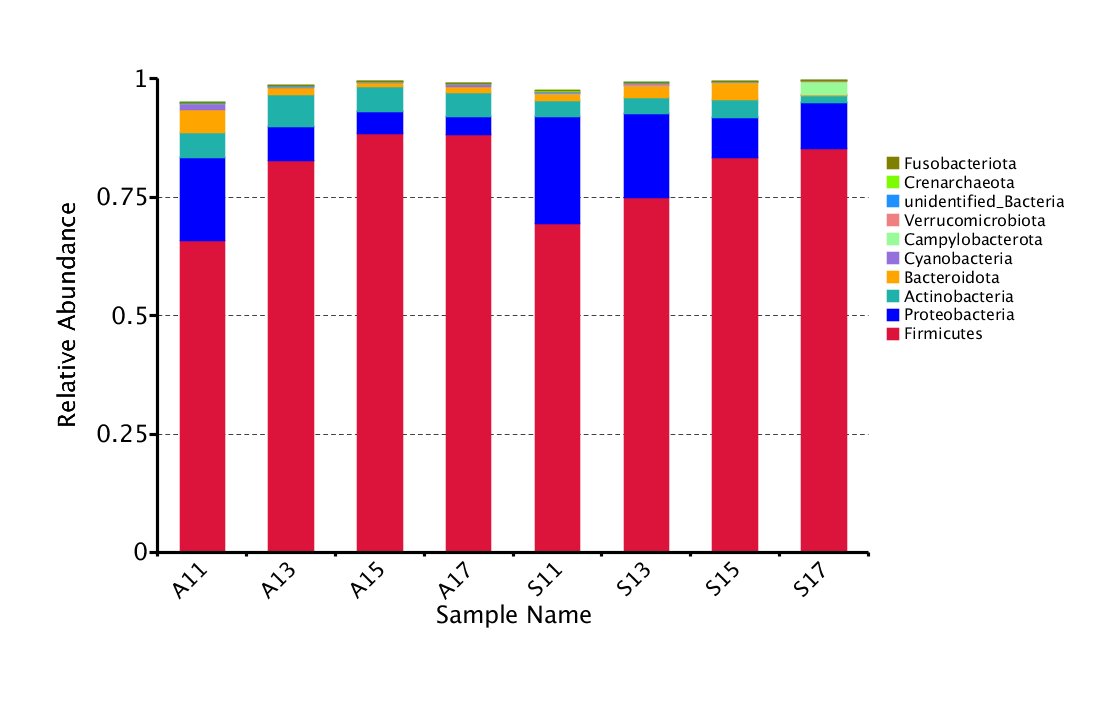


**Supplementary Figure 4.** Histogram of relative abundance of gut microbiota at the phylum level. S11, gut microbiota of the SGA group on day 1; A11, gut microbiota of the AGA group on day 1; S13, gut microbiota of the SGA group on day 3; A13, gut microbiota of the AGA group on day 3; S15, gut microbiota of the SGA group on day 5; A15, gut microbiota of the AGA group on day 5; S17, gut microbiota of the SGA group on day 7; A17, gut microbiota of the AGA group on day 7.

**
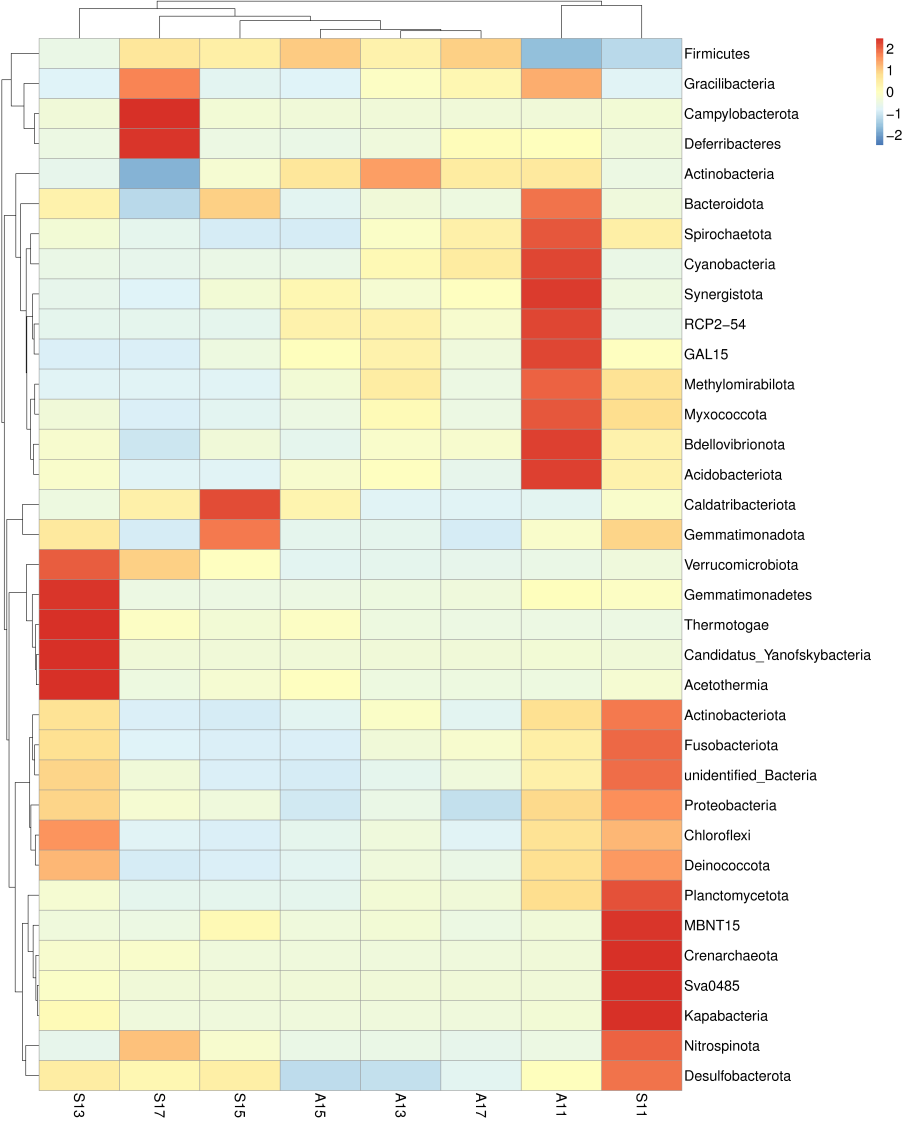
**

Supplementary Figure 5. Heatmap of taxonomy abundance in each group at the phylum level. S11, gut microbiota of the SGA group on day 1; A11, gut microbiota of the AGA group on day 1; S13, gut microbiota of the SGA group on day 3; A13, gut microbiota of the AGA group on day 3; S15, gut microbiota of the SGA group on day 5; A15, gut microbiota of the AGA group on day 5; S17, gut microbiota of the SGA group on day 7; A17, gut microbiota of the AGA group on day 7.


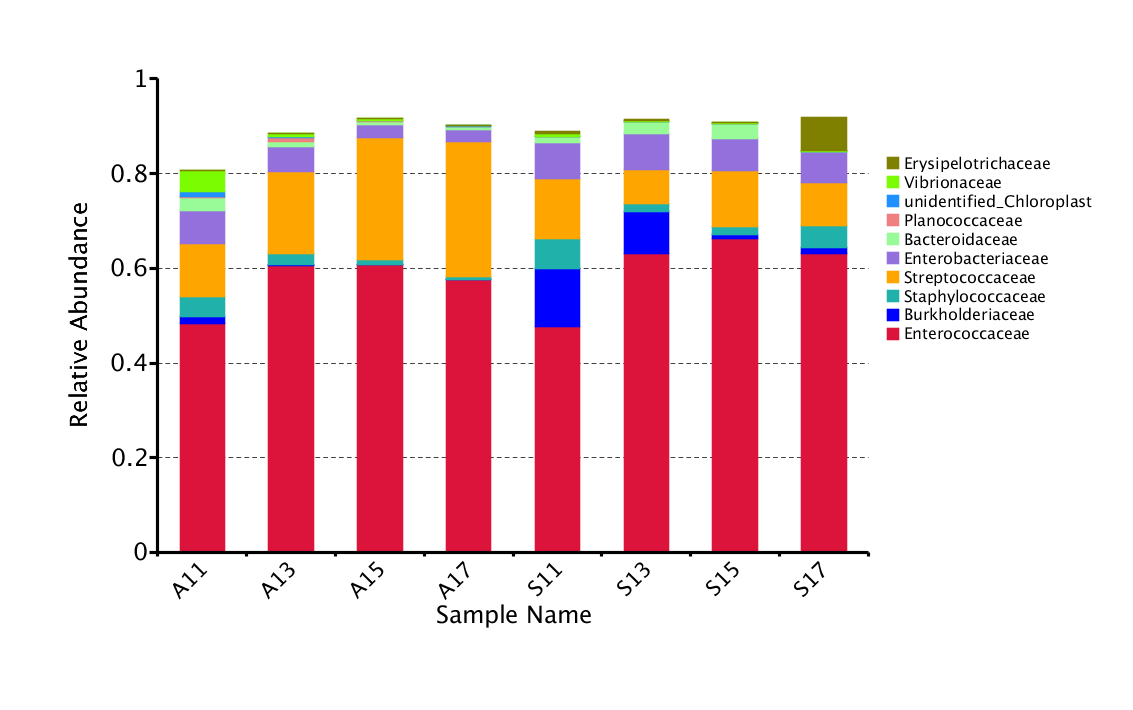


**Supplementary Figure 6.** Histogram of relative abundance of gut microbiota between the SGA and AGA groups at the family level. S11, gut microbiota of the SGA group on day 1; A11, gut microbiota of the AGA group on day 1; S13, gut microbiota of the SGA group on day 3; A13, gut microbiota of the AGA group on day 3; S15, gut microbiota of the SGA group on day 5; A15, gut microbiota of the AGA group on day 5; S17, gut microbiota of the SGA group on day 7; A17, gut microbiota of the AGA group on day 7.


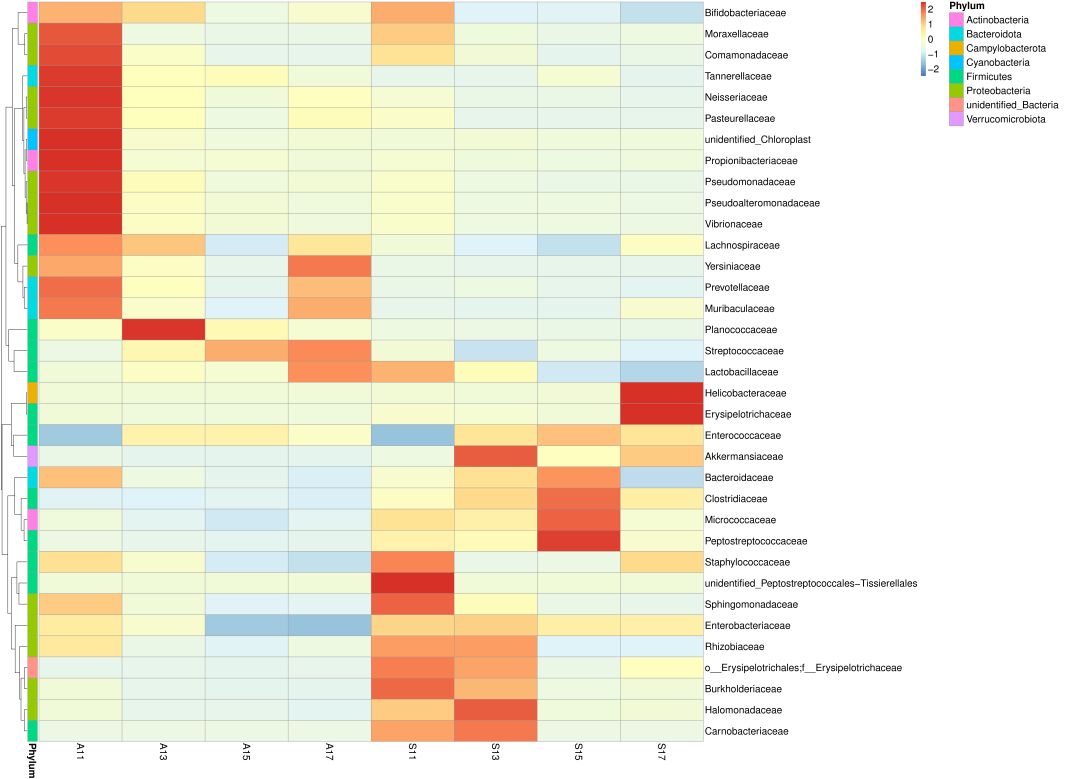


Supplementary Figure 7. Heatmap of taxonomy abundance in each group at the family level. S11, gut microbiota of the SGA group on day 1; A11, gut microbiota of the AGA group on day 1; S13, gut microbiota of the SGA group on day 3; A13, gut microbiota of the AGA group on day 3; S15, gut microbiota of the SGA group on day 5; A15, gut microbiota of the AGA group on day 5; S17, gut microbiota of the SGA group on day 7; A17, gut microbiota of the AGA group on day 7.

(A)


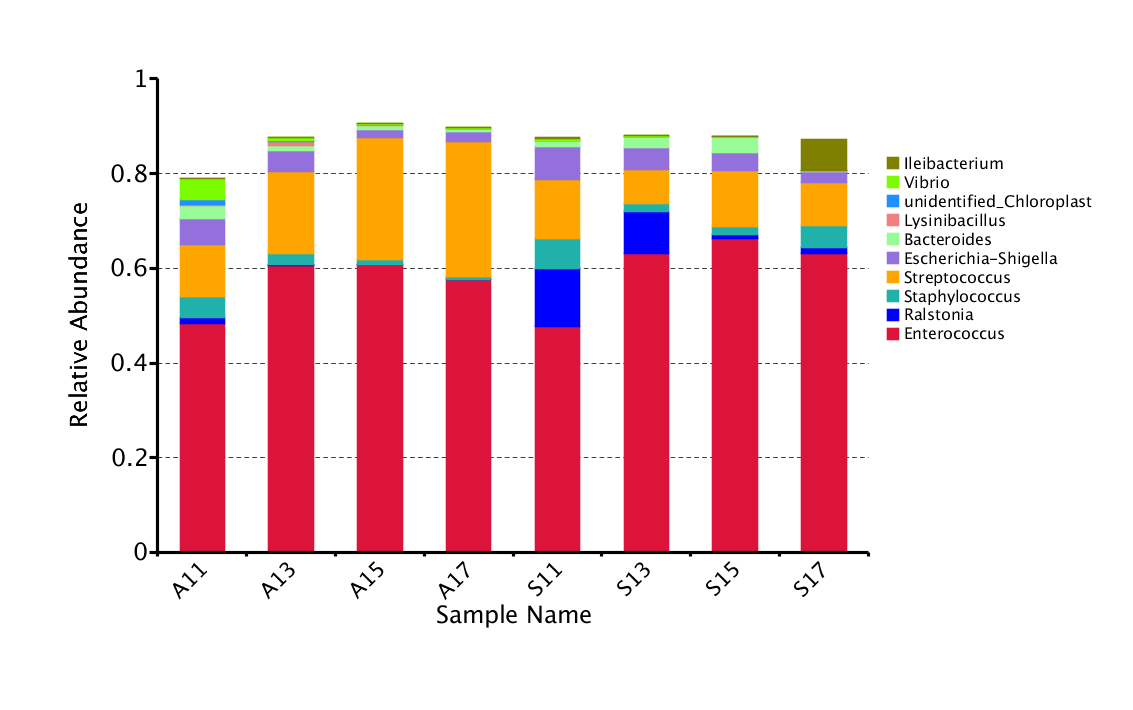


(B)


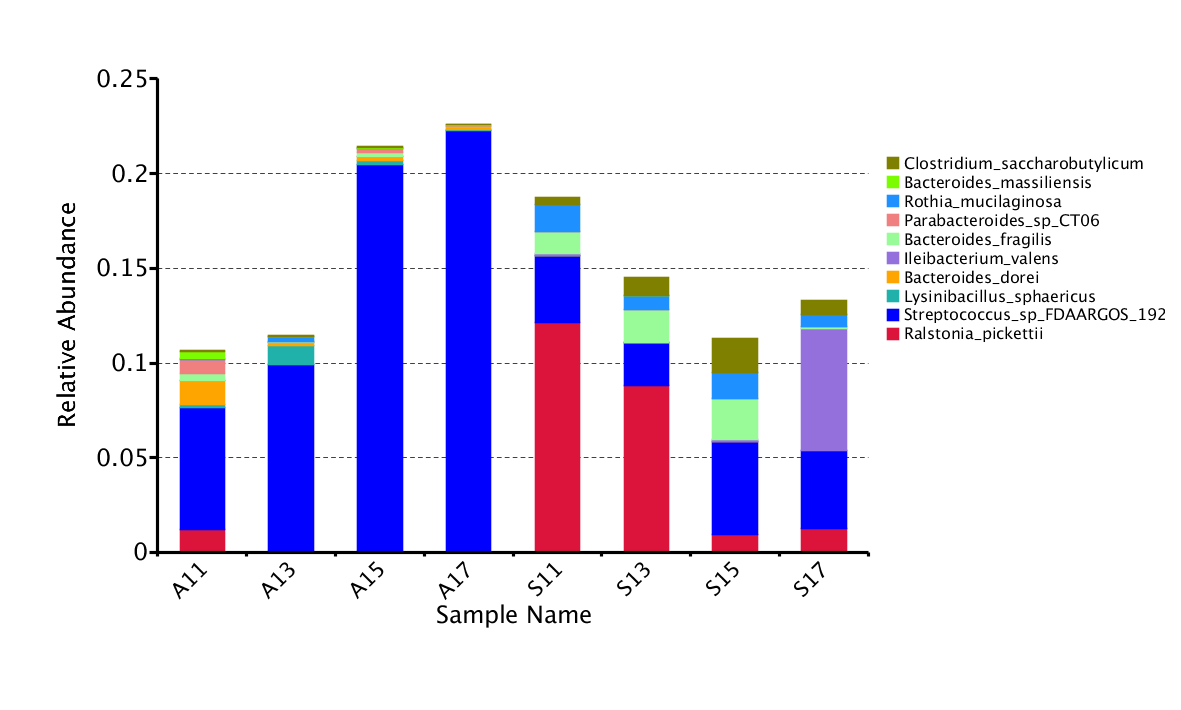


**Supplementary Figure 8.** (A) Histogram of relative abundance of gut microbiota between the SGA and AGA groups at the genus level. (B) Histogram of relative abundance of gut microbiota between the SGA and AGA groups at the species level. S11, gut microbiota of the SGA group on day 1; A11, gut microbiota of the AGA group on day 1; S13, gut microbiota of the SGA group on day 3; A13, gut microbiota of the AGA group on day 3; S15, gut microbiota of the SGA group on day 5; A15, gut microbiota of the AGA group on day 5; S17, gut microbiota of the SGA group on day 7; A17, gut microbiota of the AGA group on day 7.


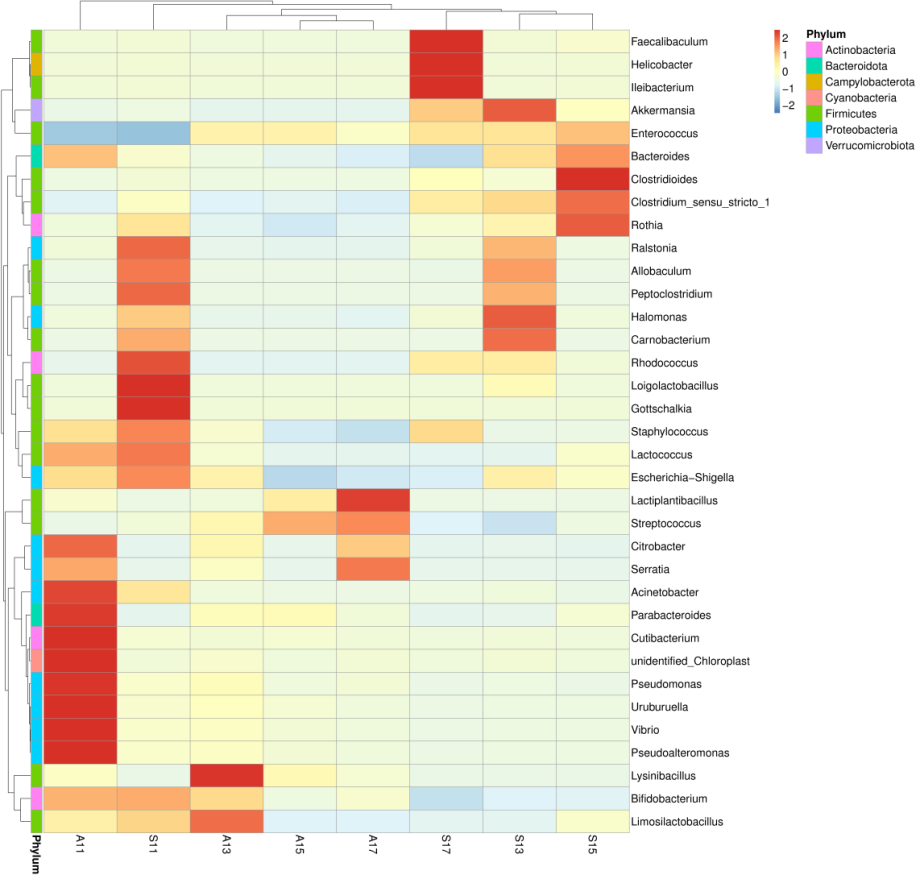


**Supplementary Figure 9.** Heatmap of taxonomy abundance in each group at the genus level. S11, gut microbiota of the SGA group on day 1; A11, gut microbiota of the AGA group on day 1; S13, gut microbiota of the SGA group on day 3; A13, gut microbiota of the AGA group on day 3; S15, gut microbiota of the SGA group on day 5; A15, gut microbiota of the AGA group on day 5; S17, gut microbiota of the SGA group on day 7; A17, gut microbiota of the AGA group on day 7.


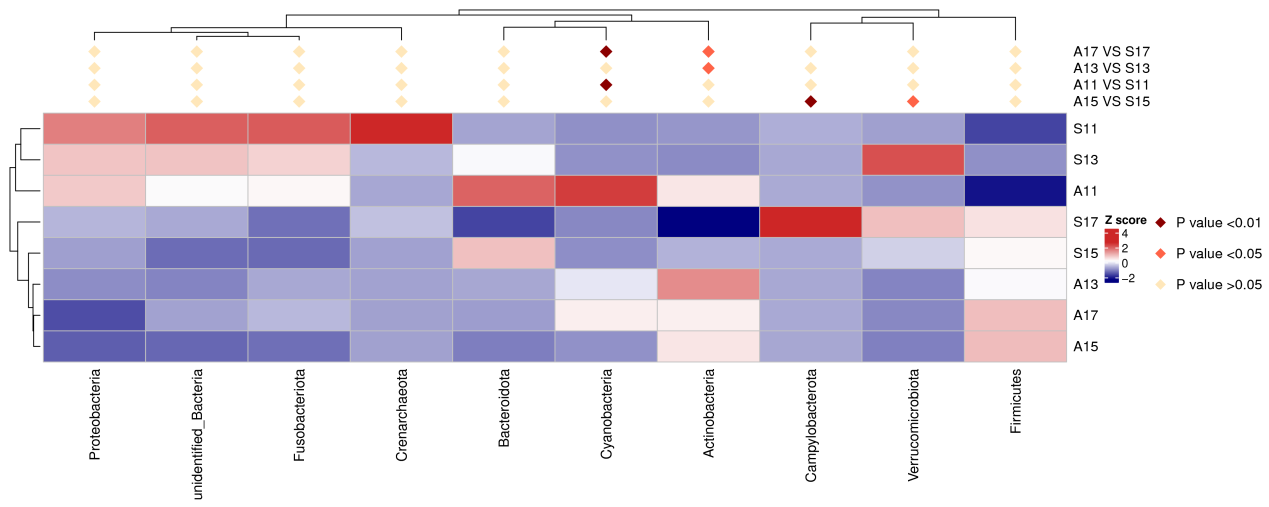


Supplementary Figure 10. Results of heatmap analysis of species with significant difference between the SGA and AGA groups at the phylum level. S11, gut microbiota of the SGA group on day 1; A11, gut microbiota of the AGA group on day 1; S13, gut microbiota of the SGA group on day 3; A13, gut microbiota of the AGA group on day 3; S15, gut microbiota of the SGA group on day 5; A15, gut microbiota of the AGA group on day 5; S17, gut microbiota of the SGA group on day 7; A17, gut microbiota of the AGA group on day 7.


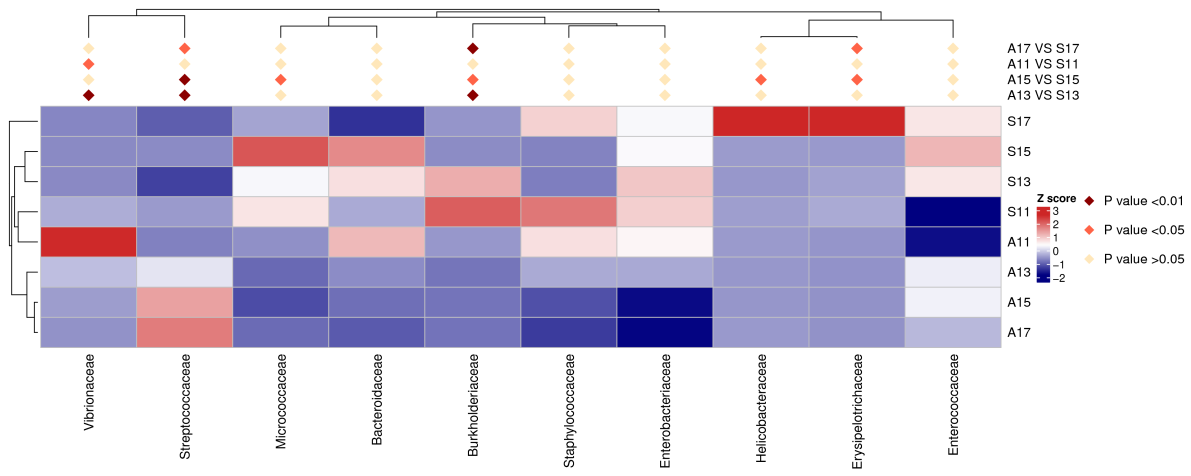


Supplementary Figure 11. Results of heatmap analysis of species with significant difference between the SGA and AGA groups at the family level. S11, gut microbiota of the SGA group on day 1; A11, gut microbiota of the AGA group on day 1; S13, gut microbiota of the SGA group on day 3; A13, gut microbiota of the AGA group on day 3; S15, gut microbiota of the SGA group on day 5; A15, gut microbiota of the AGA group on day 5; S17, gut microbiota of the SGA group on day 7; A17, gut microbiota of the AGA group on day 7.

(A) (B)

**
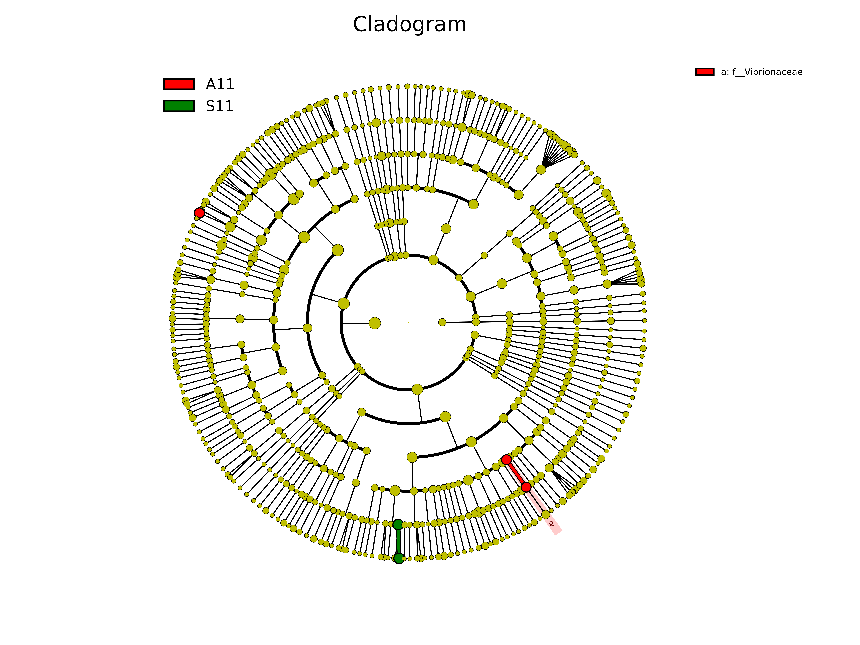
**
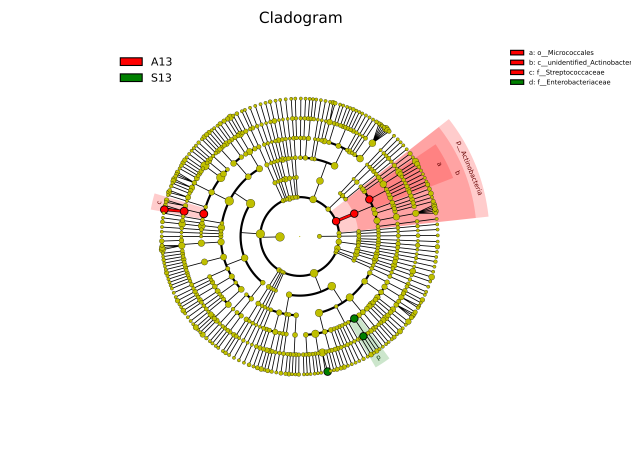


(C) (D)


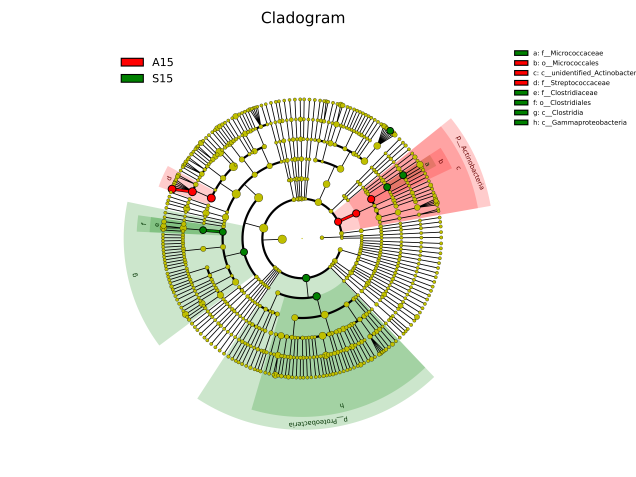

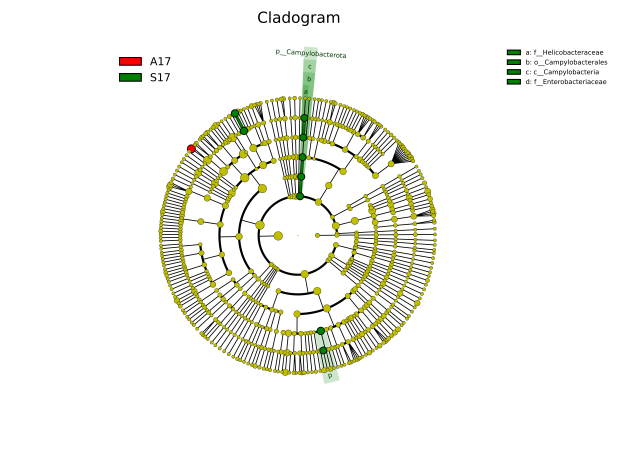


Supplementary Figure 12. Cladograms of the SGA and AGA groups on days 1, 3, 5, and 7. (A) Cladogram on day 1. (B) Cladogram on day 3. (C) Cladogram on day 5. (D) Cladogram on day 7. S11, gut microbiota of the SGA group on day 1; A11, gut microbiota of the AGA group on day 1; S13, gut microbiota of the SGA group on day 3; A13, gut microbiota of the AGA group on day 3; S15, gut microbiota of the SGA group on day 5; A15, gut microbiota of the AGA group on day 5; S17, gut microbiota of the SGA group on day 7; A17, gut microbiota of the AGA group on day 7.

(A) (B)


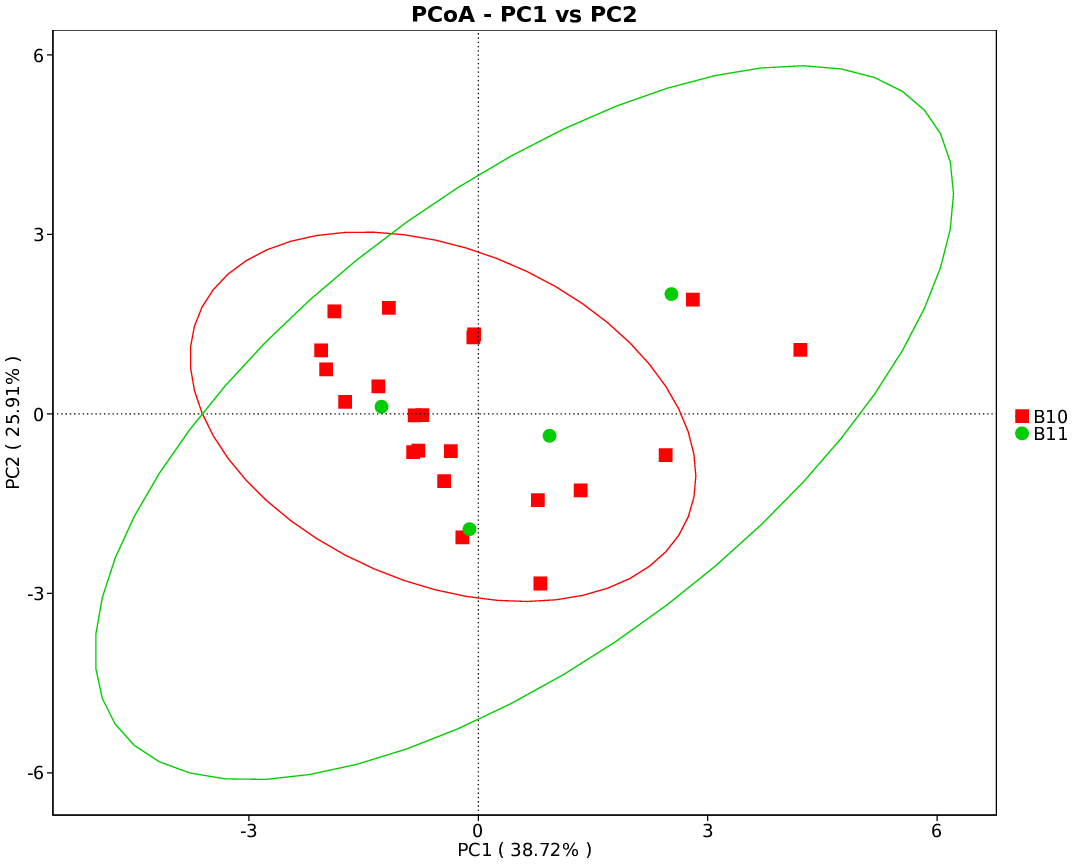

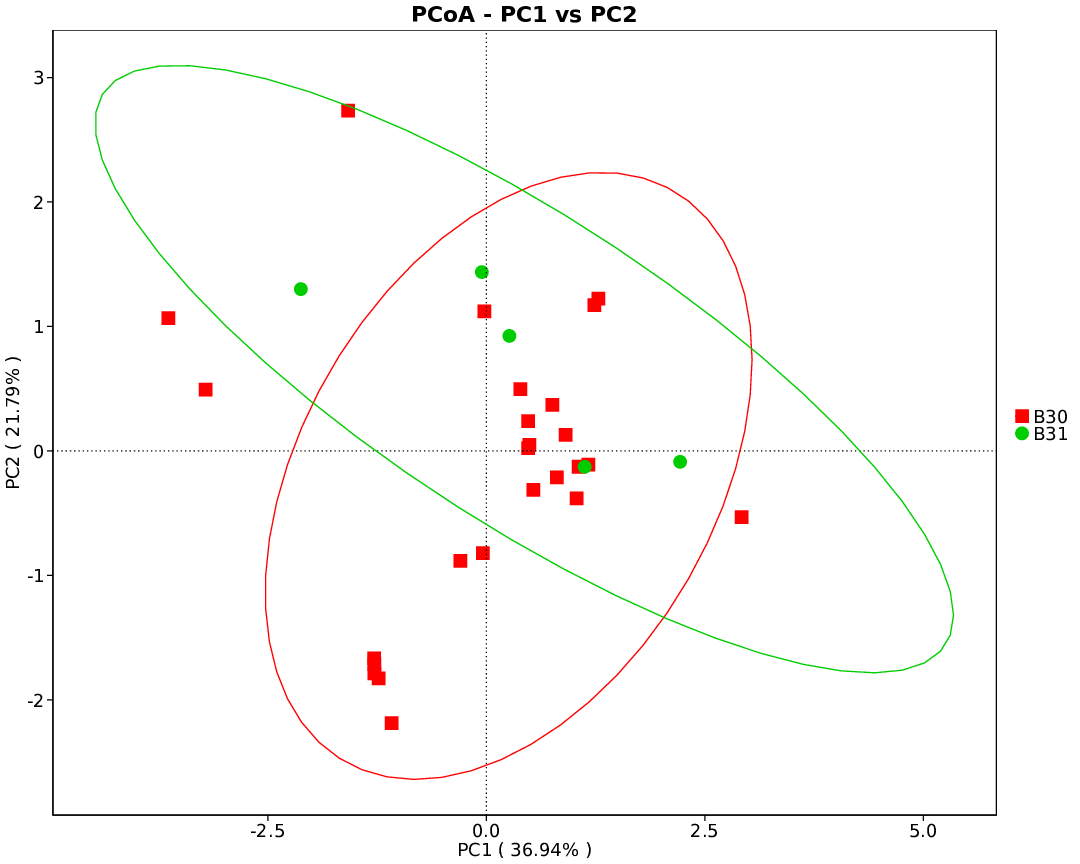


(C) (D)


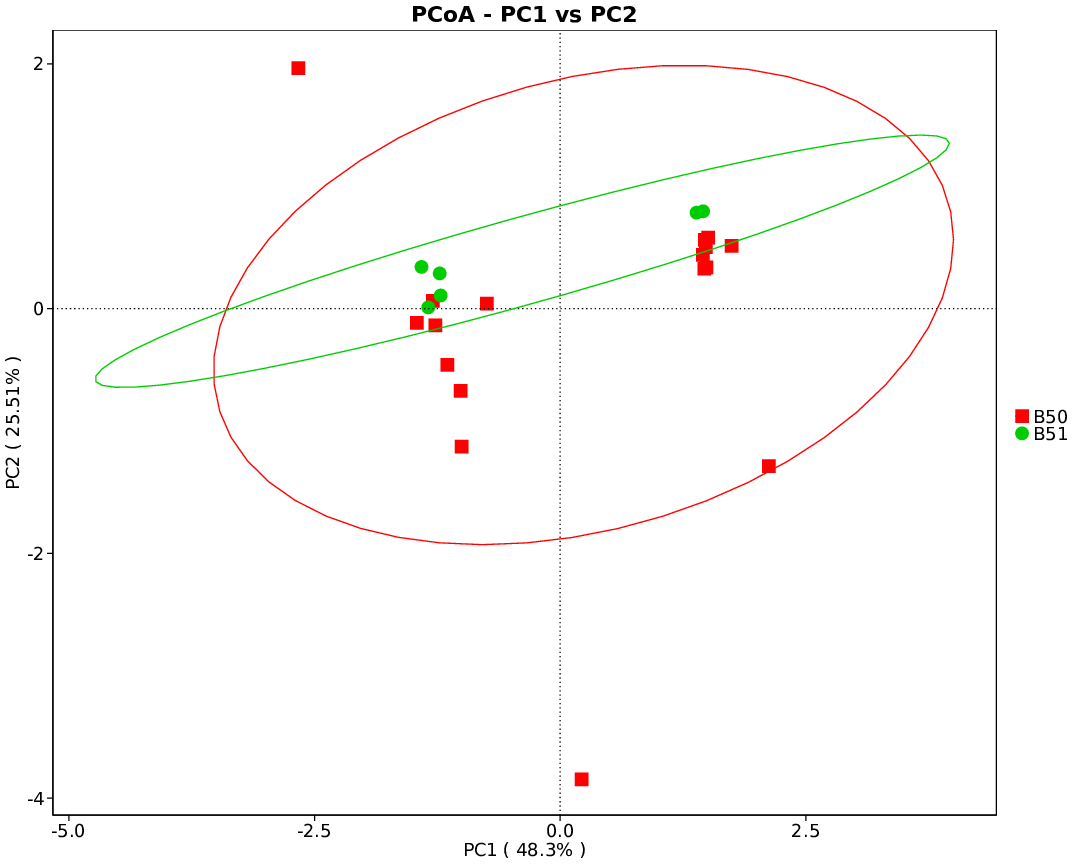

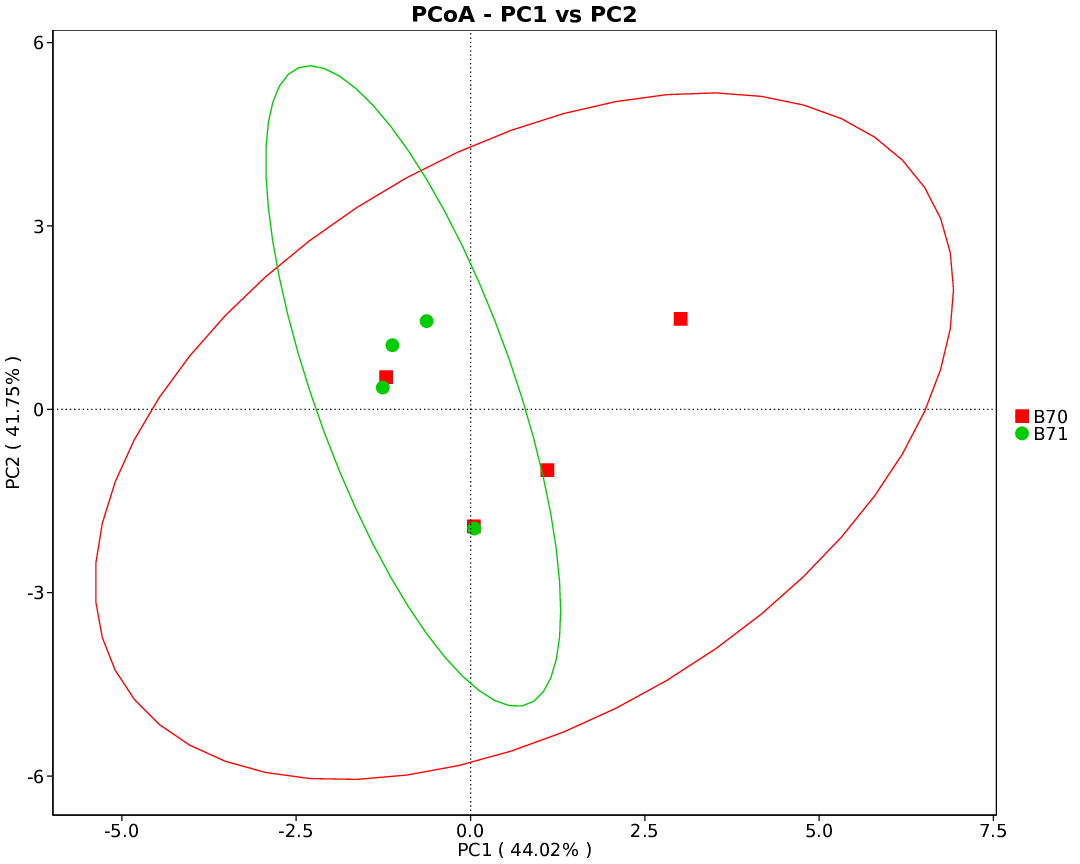


Supplementary Figure 13. PCoA analysis between the good and poor communication score groups on days 1, 3, 5, and 7. (A) PCoA analysis on day 1. (B) PCoA analysis on day 3. (C) PCoA analysis on day 5. (D) PCoA analysis on day 7. B11, gut microbiota of the poor communication score group on day 1; B10, gut microbiota of the good communication score group on day 1; B31, gut microbiota of the poor communication score group on day 3; B30, gut microbiota of the good communication score group on day 3; B51, gut microbiota of the poor communication score group on day 5; B50, gut microbiota of the good communication score group on day 5; B71, gut microbiota of the poor communication score group on day 7; B70, gut microbiota of the good communication score group on day 7.

(A) (B)

**
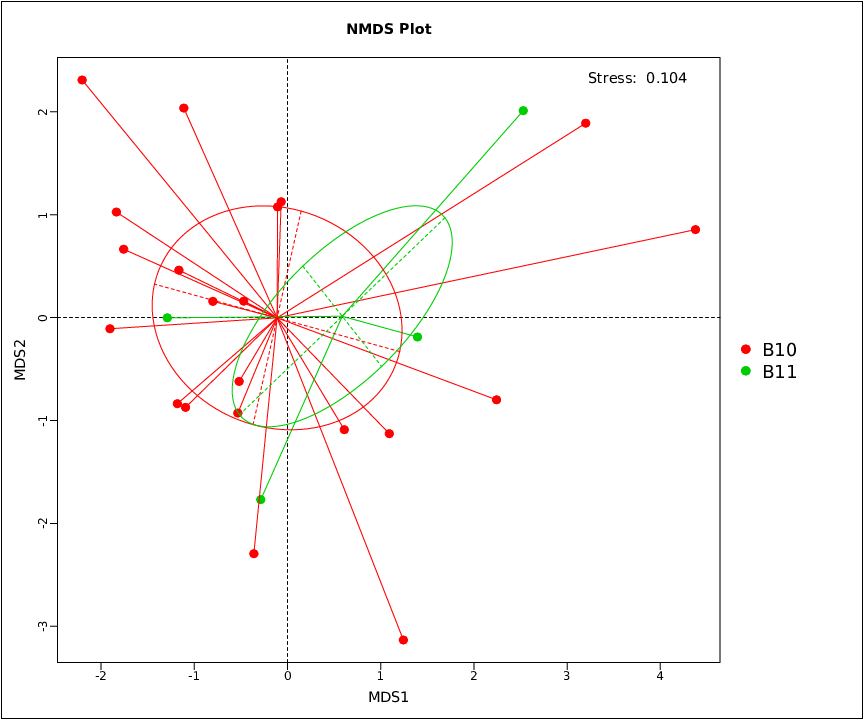

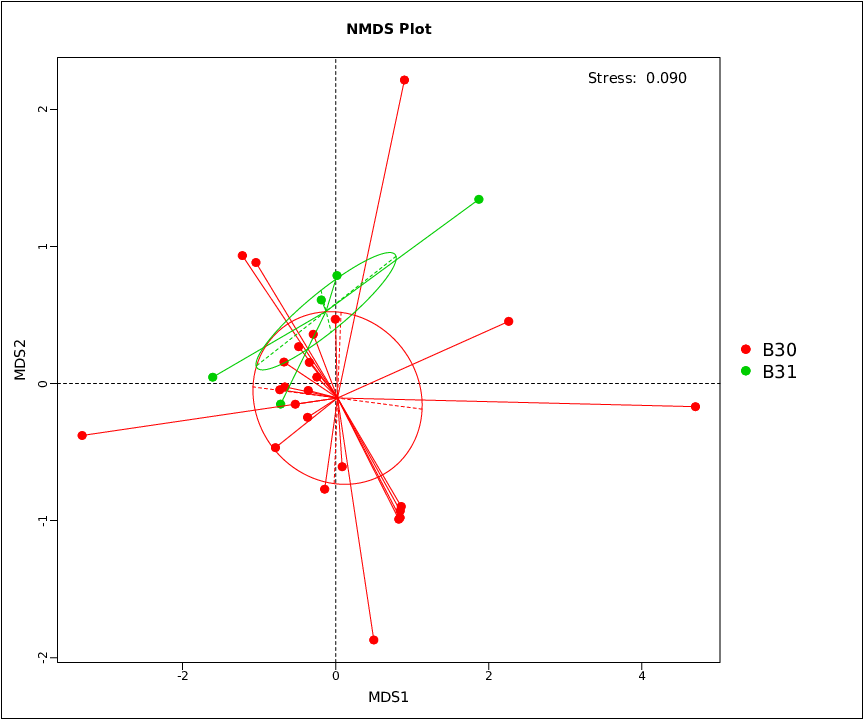
**

(C) (D)

**
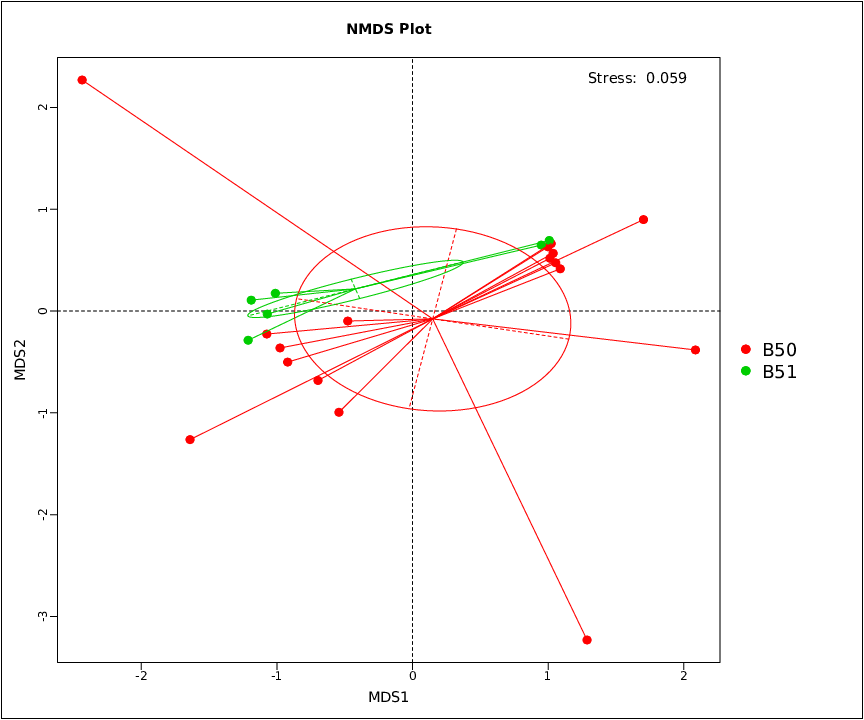

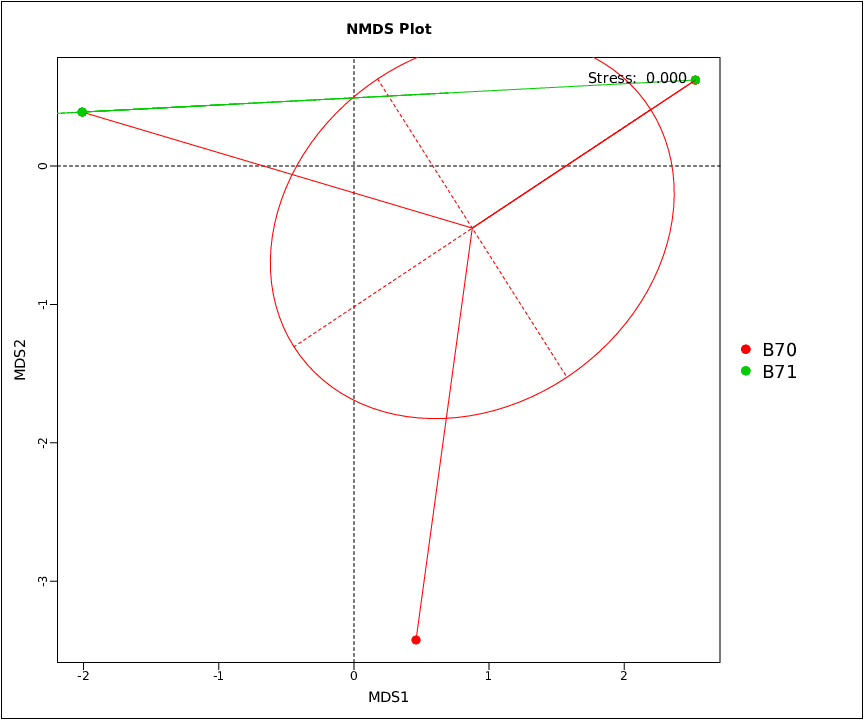
**

Supplementary Figure 14. NMDS analysis between the good and poor communication score groups on days 1, 3, 5, and 7. (A) NMDS analysis on day 1. (B) NMDS analysis on day 3. (C) NMDS analysis on day 5. (D) NMDS analysis on day 7. B11, gut microbiota of the poor communication score group on day 1; B10, gut microbiota of the good communication score group on day 1; B31, gut microbiota of the poor communication score group on day 3; B30, gut microbiota of the good communication score group on day 3; B51, gut microbiota of the poor communication score group on day 5; B50, gut microbiota of the good communication score group on day 5; B71, gut microbiota of the poor communication score group on day 7; B70, gut microbiota of the good communication score group on day 7.


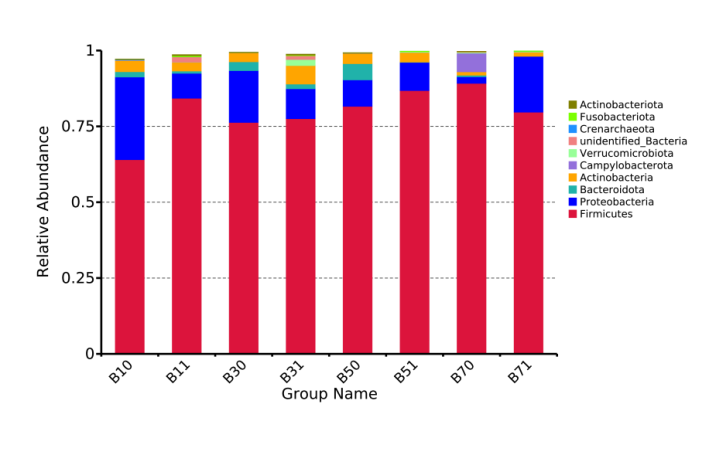


**Supplementary Figure 15.** Histogram of relative abundance of gut microbiota between the good and poor communication score groups at the phylum level. B11, gut microbiota of the poor communication score group on day 1; B10, gut microbiota of the good communication score group on day 1; B31, gut microbiota of the poor communication score group on day 3; B30, gut microbiota of the good communication score group on day 3; B51, gut microbiota of the poor communication score group on day 5; B50, gut microbiota of the good communication score group on day 5; B71, gut microbiota of the poor communication score group on day 7; B70, gut microbiota of the good communication score group on day 7.


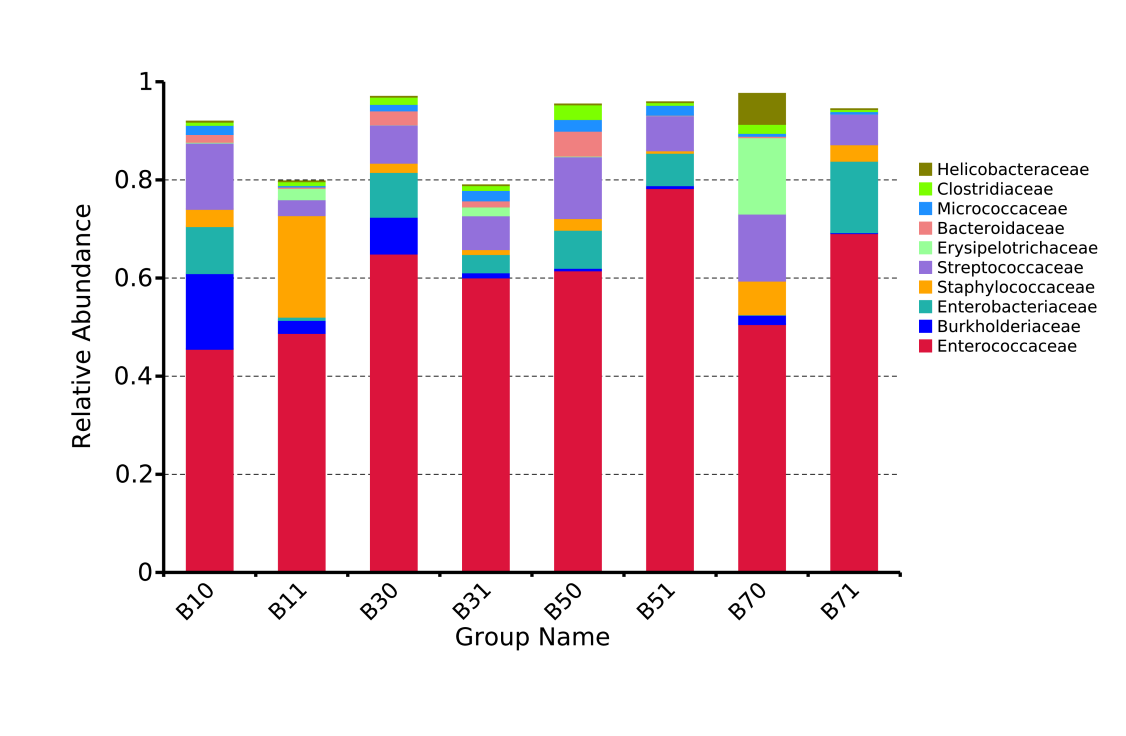


**Supplementary Figure 16.** Histogram of relative abundance of gut microbiota between the good and poor communication score groups at the family level. B11, gut microbiota of the poor communication score group on day 1; B10, gut microbiota of the good communication score group on day 1; B31, gut microbiota of the poor communication score group on day 3; B30, gut microbiota of the good communication score group on day 3; B51, gut microbiota of the poor communication score group on day 5; B50, gut microbiota of the good communication score group on day 5; B71, gut microbiota of the poor communication score group on day 7; B70, gut microbiota of the good communication score group on day 7.


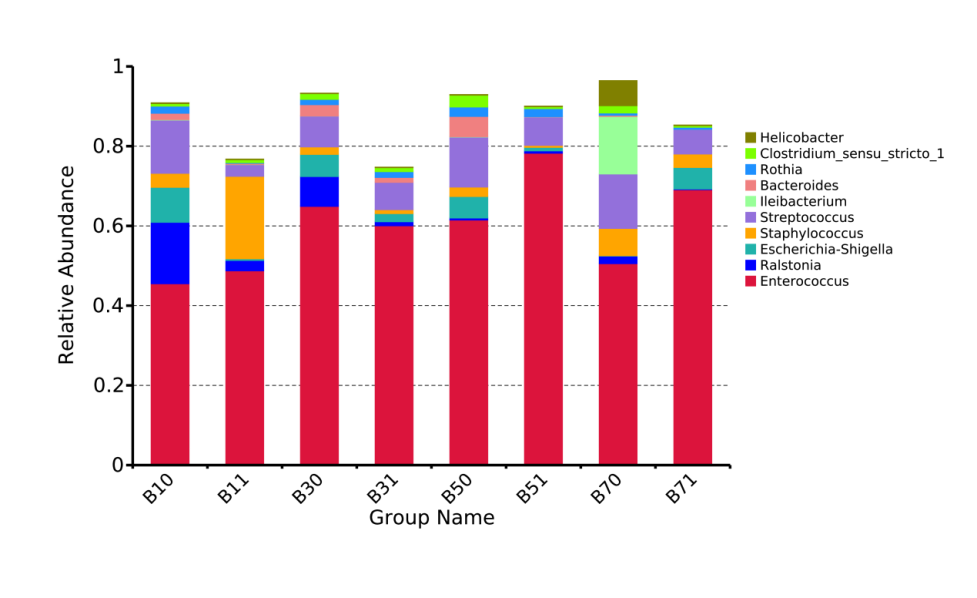


Supplementary Figure 17. Histogram of relative abundance of gut microbiota between the good and poor communication score groups at the genus level. B11, gut microbiota of the poor communication score group on day 1; B10, gut microbiota of the good communication score group on day 1; B31, gut microbiota of the poor communication score group on day 3; B30, gut microbiota of the good communication score group on day 3; B51, gut microbiota of the poor communication score group on day 5; B50, gut microbiota of the good communication score group on day 5; B71, gut microbiota of the poor communication score group on day 7; B70, gut microbiota of the good communication score group on day 7.


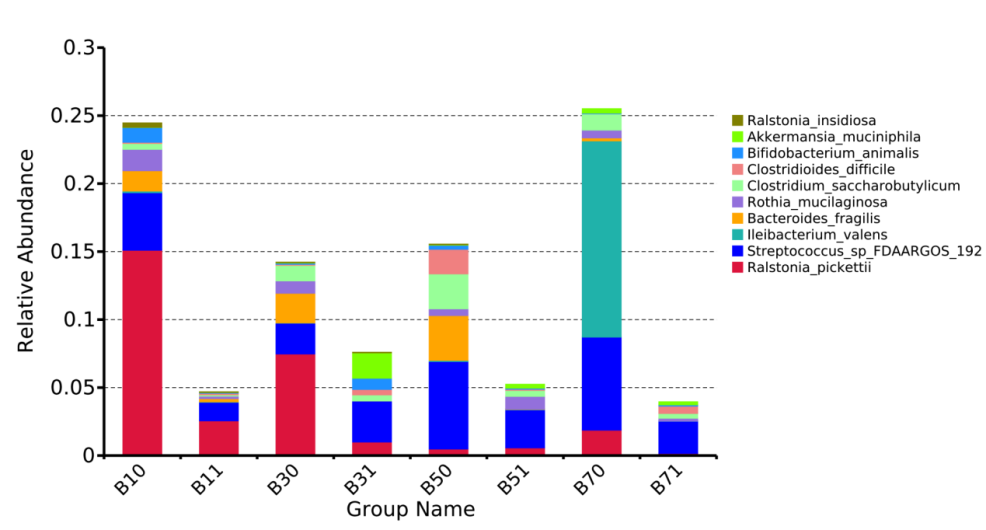


Supplementary Figure 18. Histogram of relative abundance of gut microbiota between the good and poor communication score groups at the species level. B11, gut microbiota of the poor communication score group on day 1; B10, gut microbiota of the good communication score group on day 1; B31, gut microbiota of the poor communication score group on day 3; B30, gut microbiota of the good communication score group on day 3; B51, gut microbiota of the poor communication score group on day 5; B50, gut microbiota of the good communication score group on day 5; B71, gut microbiota of the poor communication score group on day 7; B70, gut microbiota of the good communication score group on day 7.

(A)


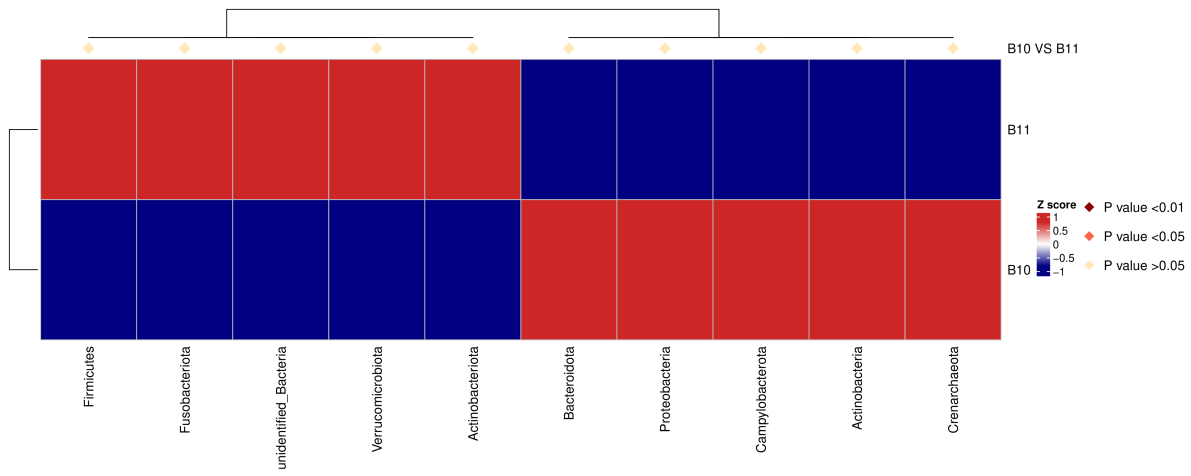


(B)


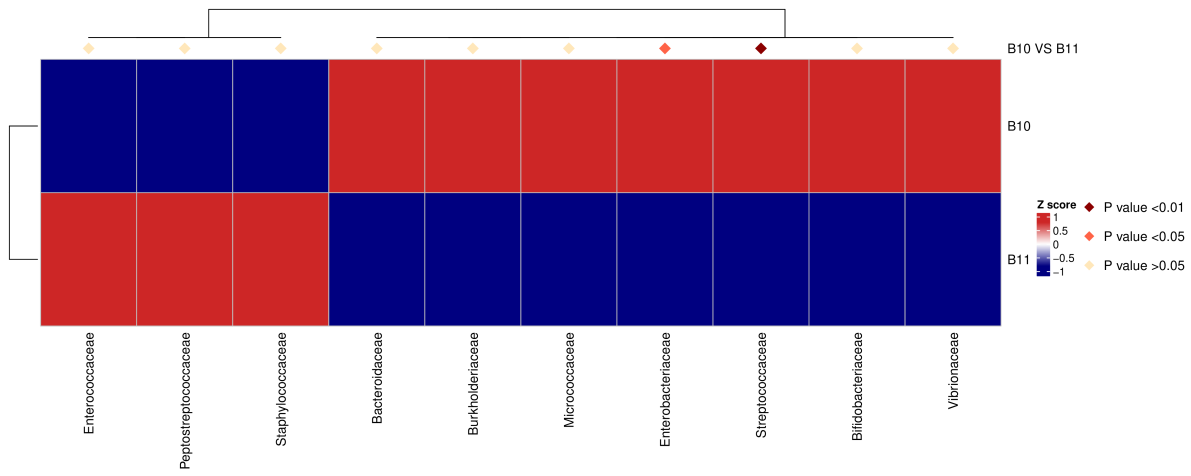


(C)


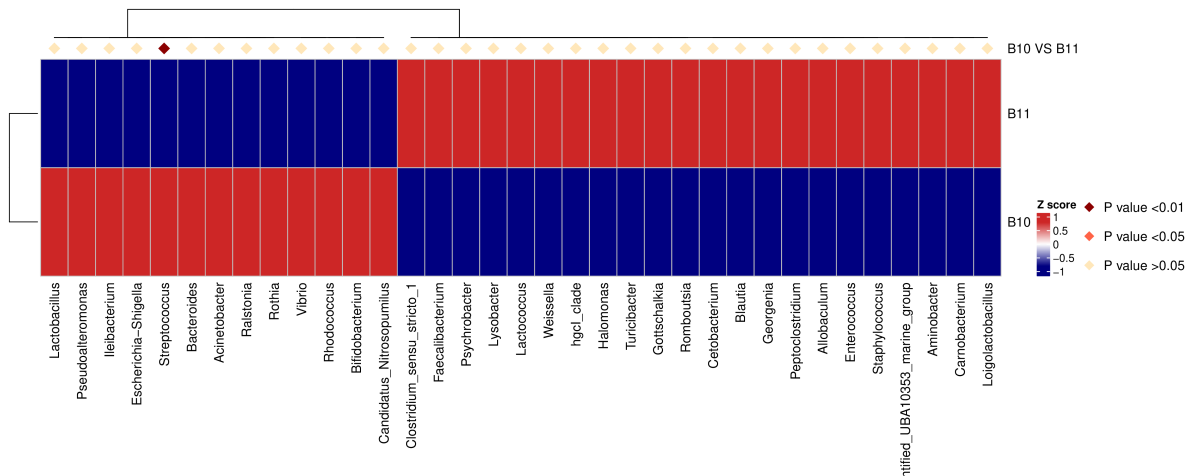


(D)


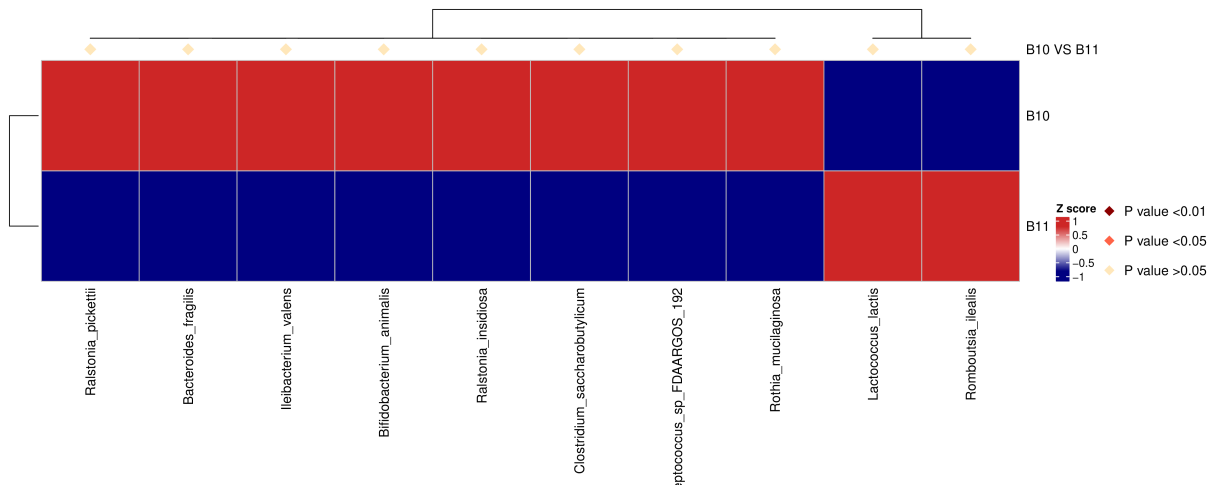


Supplementary Figure 19. Results of heatmap analysis of species with significant difference between the good and poor communication score groups at levels of phylum, family, genus and species on day 1. (A) Heatmap analysis at the phylum level; (B) Heatmap analysis at the family level; (C) Heatmap analysis at the genus level; (D) Heatmap analysis at the species level. B11, gut microbiota of the poor communication score group on day 1; B10, gut microbiota of the good communication score group on day 1.

(A)


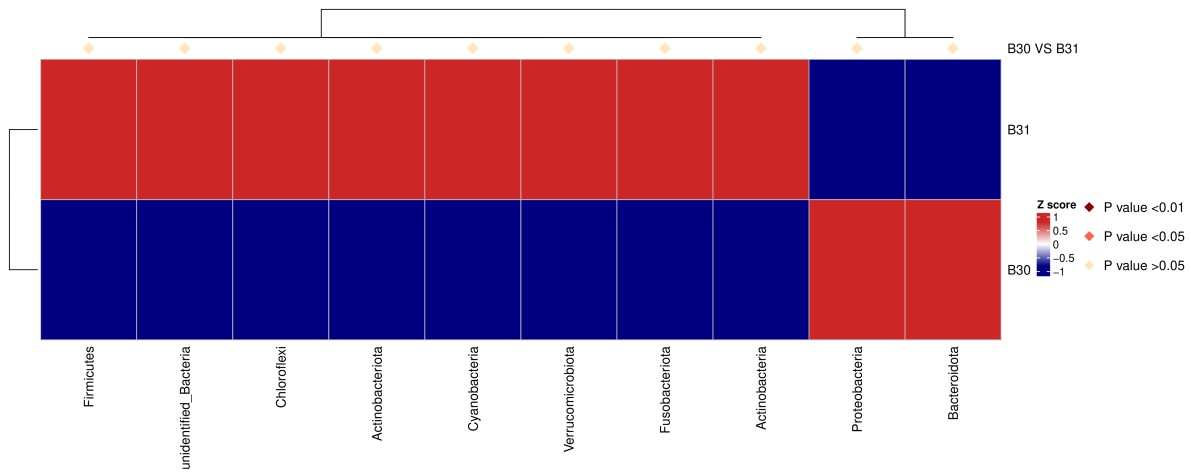


(B)


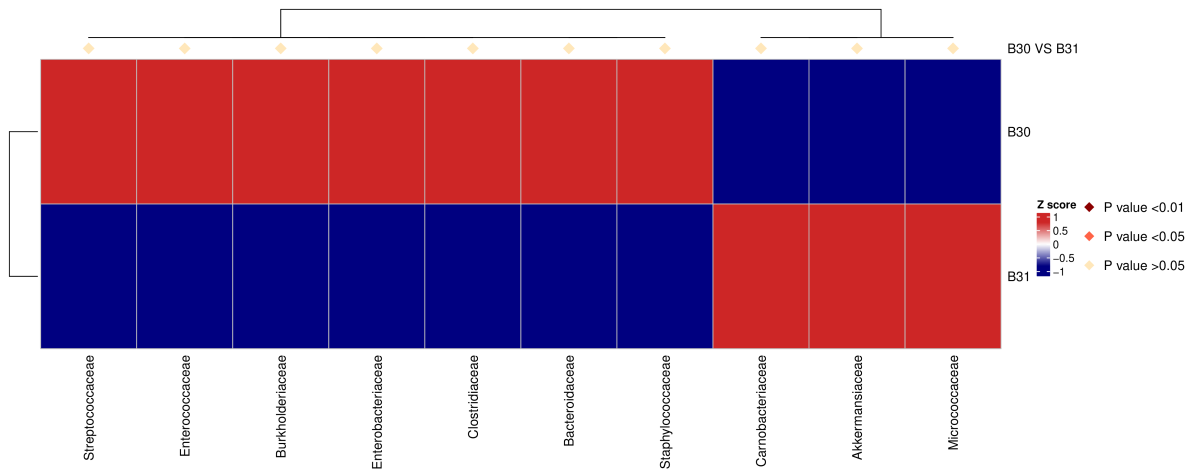


(C)

**
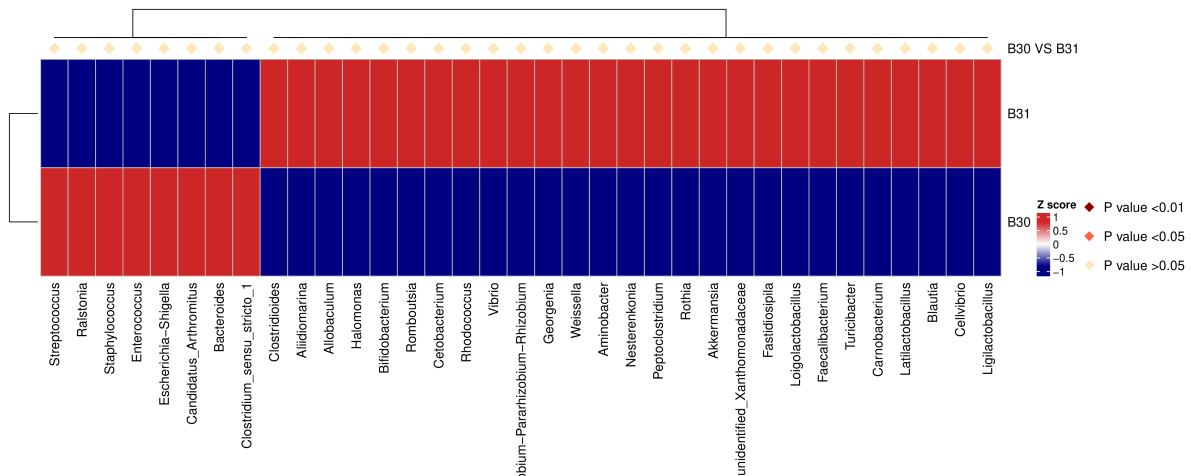
**

(D)

**
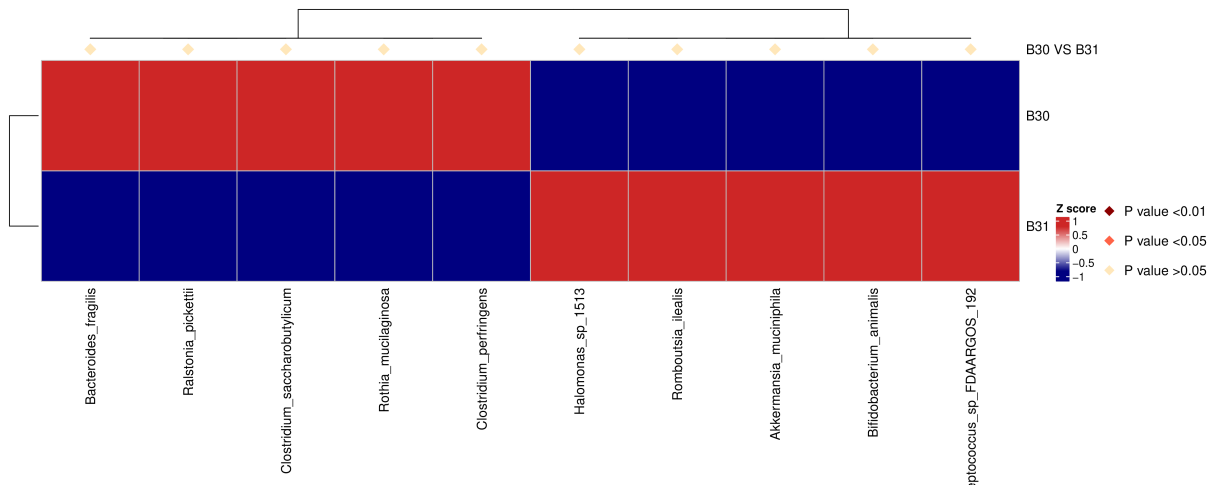
**

Supplementary Figure 20. Results of heatmap analysis of species with significant difference between the good and poor communication score groups at levels of phylum, family, genus and species on day 3. (A) Heatmap analysis at the phylum level; (B) Heatmap analysis at the family level; (C) Heatmap analysis at the genus level; (D) Heatmap analysis at the species level. B31, gut microbiota of the poor communication score group on day 3; B30, gut microbiota of the good communication score group on day 3.

(A)


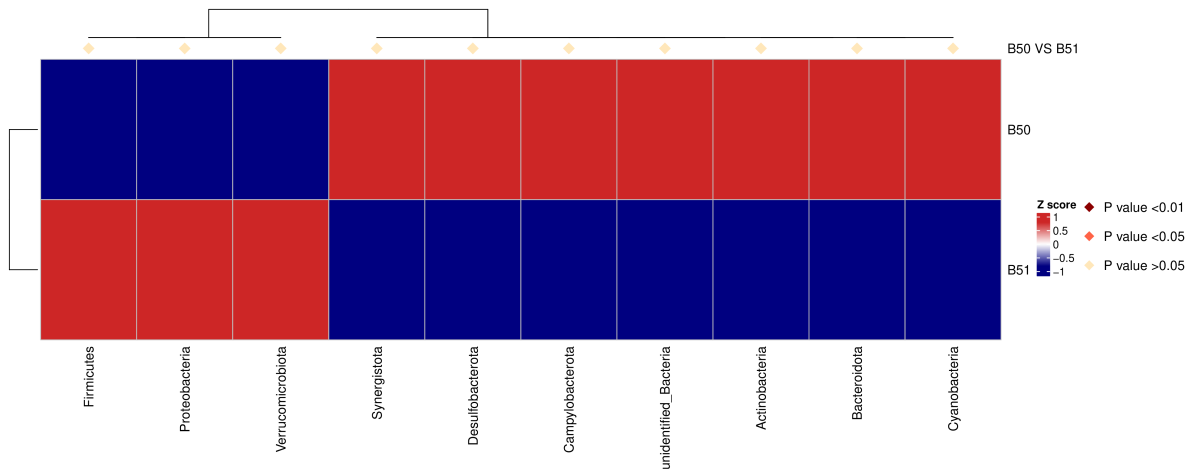


(B)


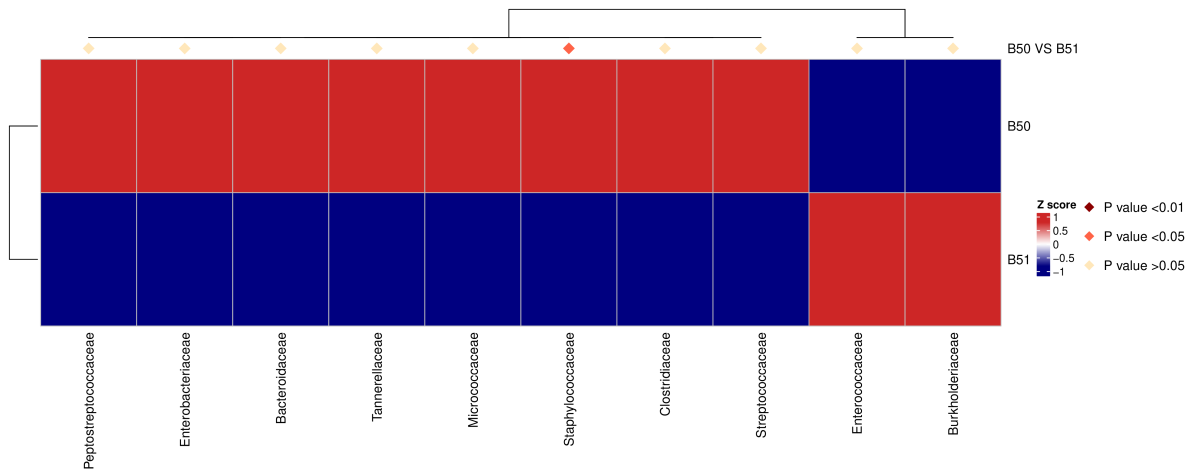


(C)

**
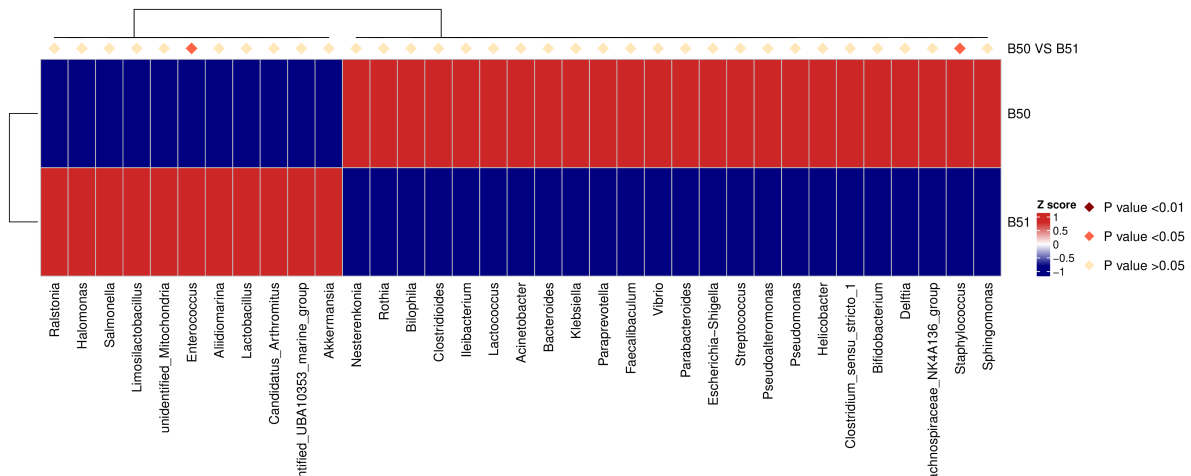
**

(D)


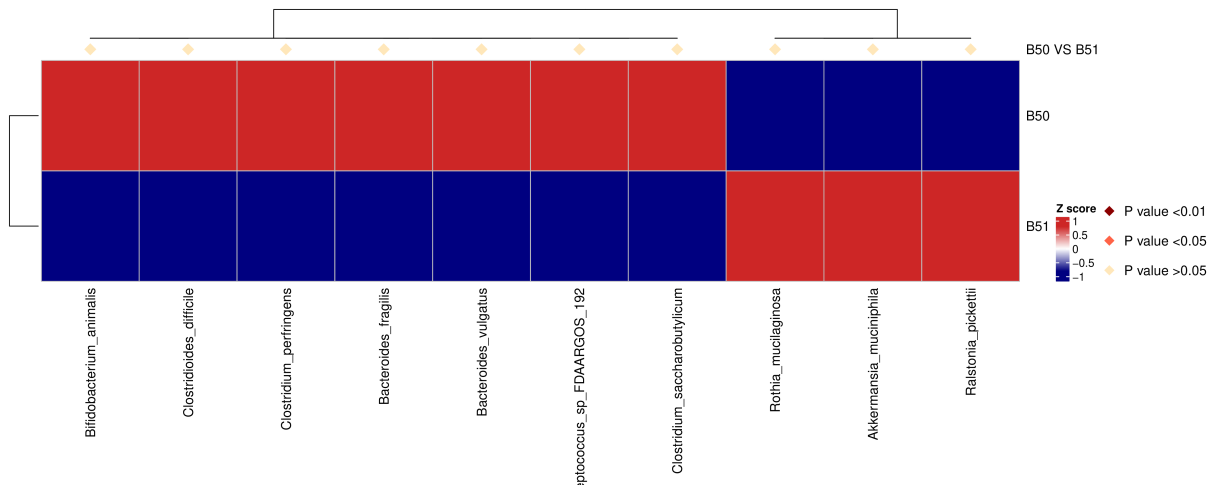


Supplementary Figure 21. Results of heatmap analysis of species with significant difference between the good and poor communication score groups at levels of phylum, family, genus and species on day 5. (A) Heatmap analysis at the phylum level; (B) Heatmap analysis at the family level; (C) Heatmap analysis at the genus level; (D) Heatmap analysis at the species level. B51, gut microbiota of the poor communication score group on day 5; B50, gut microbiota of the good communication score group on day 5.

(A)


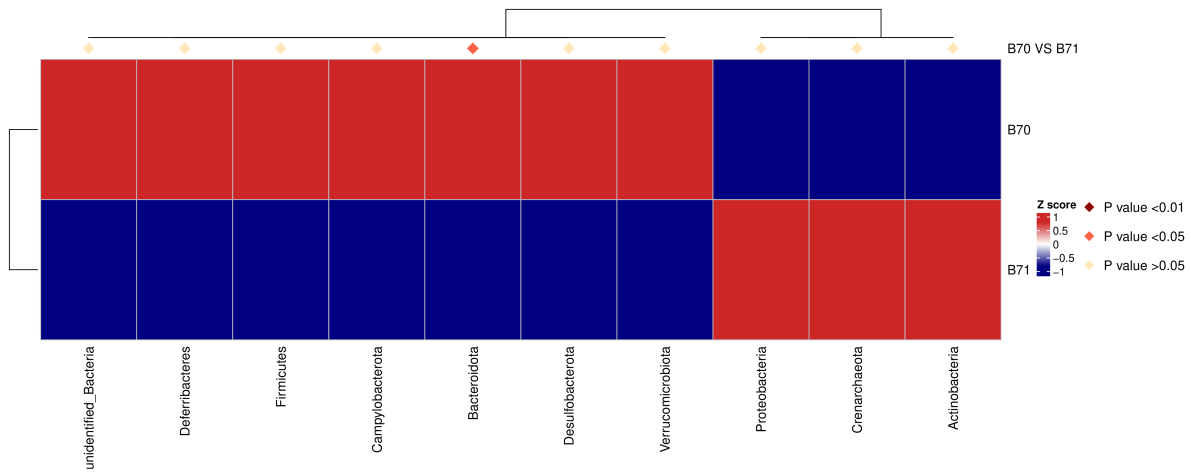


(B)


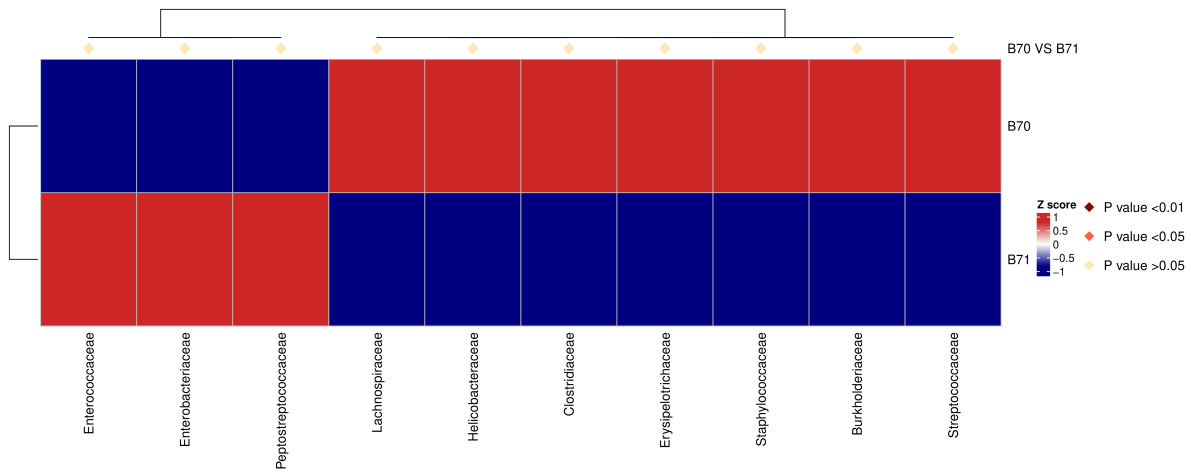


(C)

**
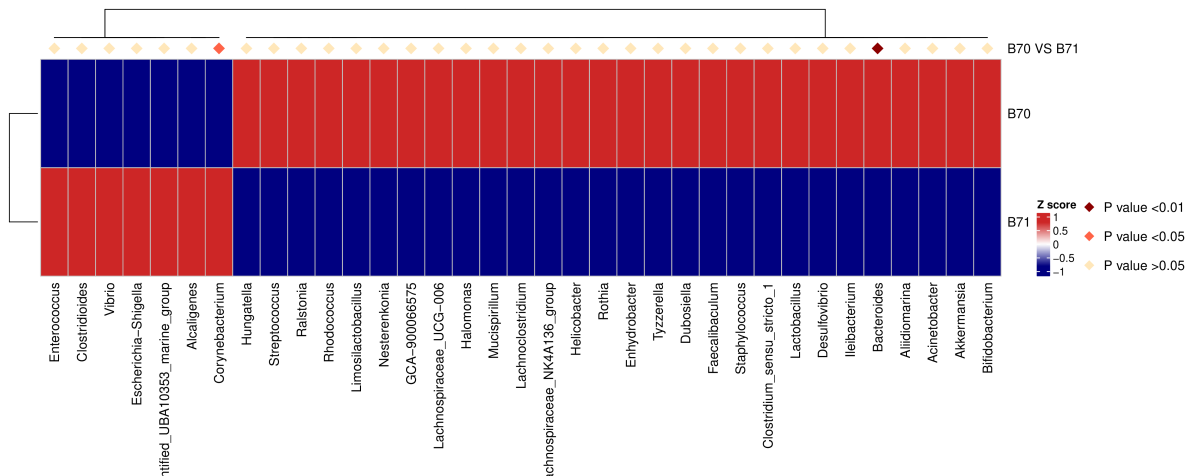
**

(D)


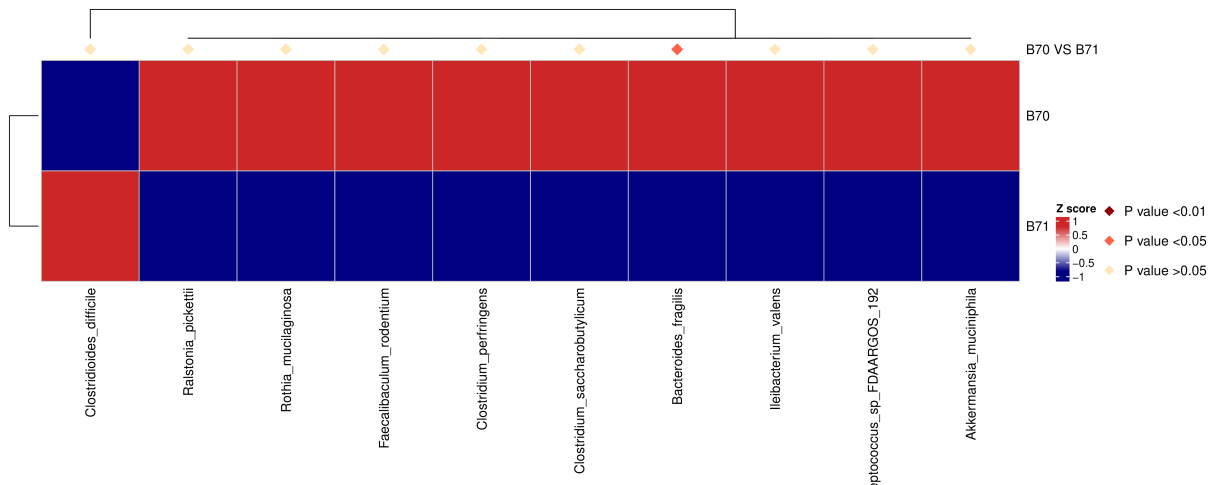


Supplementary Figure 22. Results of heatmap analysis of species with significant difference between the good and poor communication score groups at levels of phylum, family, genus and species on day 7. (A) Heatmap analysis at the phylum level; (B) Heatmap analysis at the family level; (C) Heatmap analysis at the genus level; (D) Heatmap analysis at the species level. B71, gut microbiota of the poor communication score group on day 7; B70, gut microbiota of the good communication score group on day 7.

(A) (B)


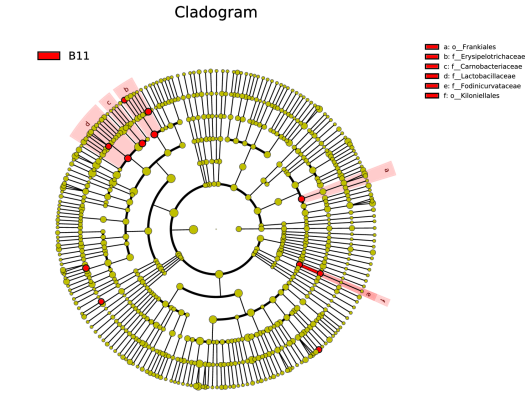

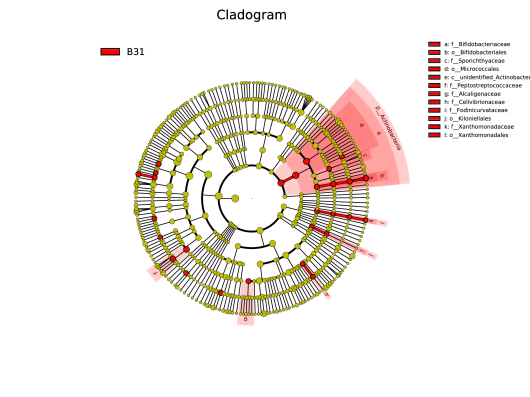


(C)


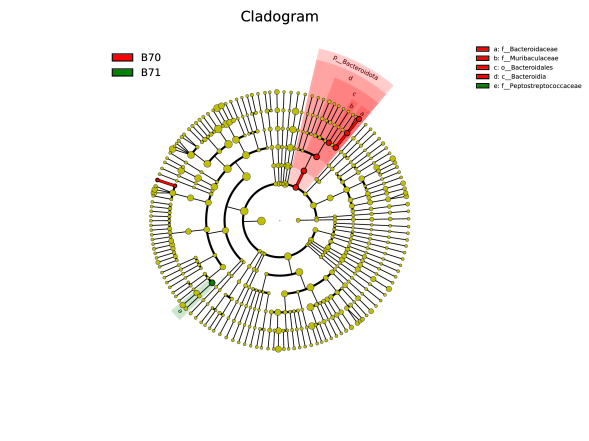


Supplementary Figure 23. Cladograms of differential microbiota of the good and poor communication score groups on days 1, 3, and 7. (A) Cladogram on day 1. (B) Cladogram on day 3. (C) Cladogram on day 7. B11, gut microbiota of the poor communication score group on day 1; B10, gut microbiota of the good communication score group on day 1; B31, gut microbiota of the poor communication score group on day 3; B30, gut microbiota of the good communication score group on day 3; B71, gut microbiota of the poor communication score group on day 7; B70, gut microbiota of the good communication score group on day 7.

(A) (B)

**
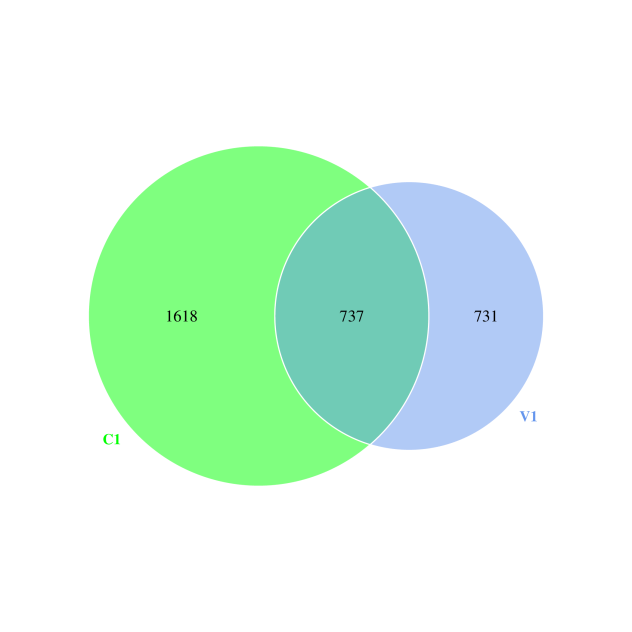

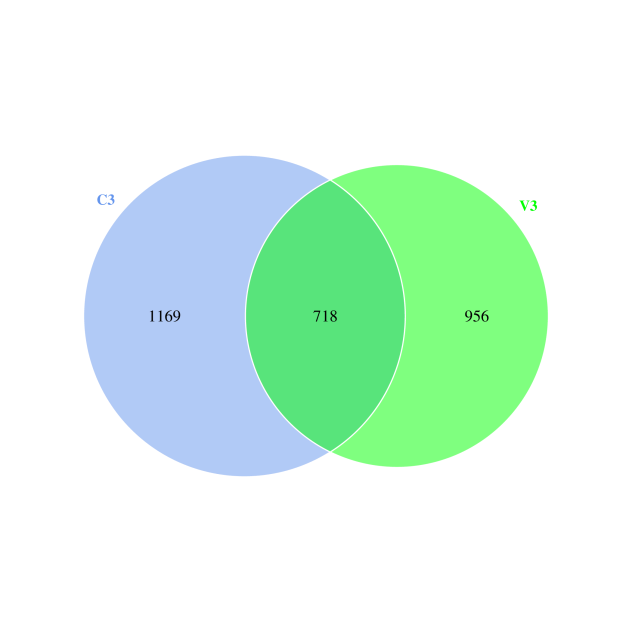
**

(C)

**
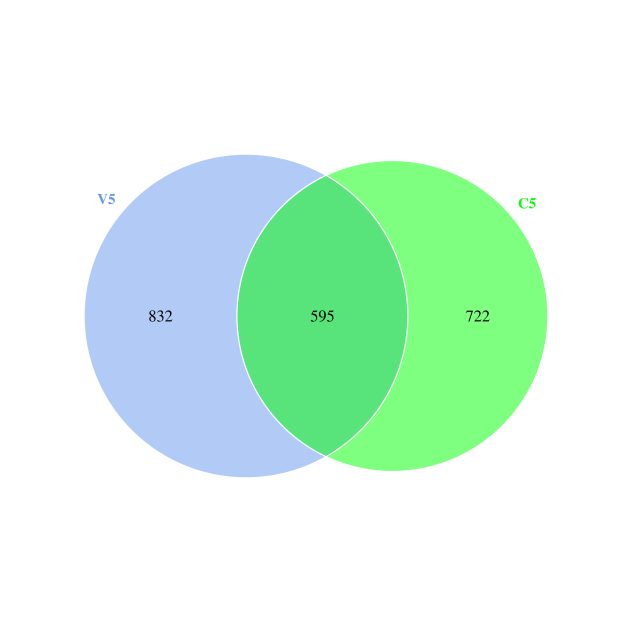
**

Supplementary Figure 24. Venn diagrams of the caesarean birth and vaginal delivery groups on days 1, 3, and 5. (A) Venn diagram on day 1; (B) Venn diagram on day 3; (C) Venn diagram on day 5. C1, gut microbiota of the caesarean birth group on day 1; V1, gut microbiota of the vaginal delivery group on day 1; C3, gut microbiota of the caesarean birth group on day 3; V3, gut microbiota of the vaginal delivery group on day 3; C5, gut microbiota of the caesarean birth group on day 5; V5, gut microbiota of the vaginal delivery group on day 5.

(A) (B)


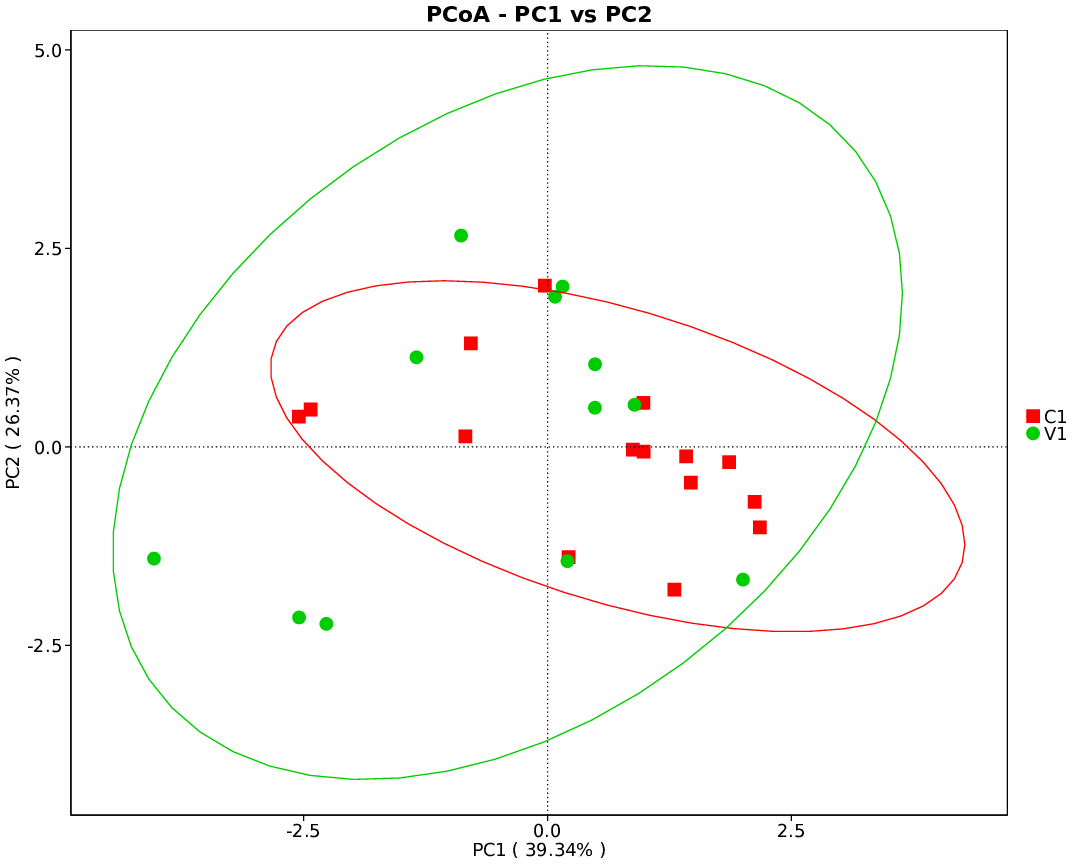

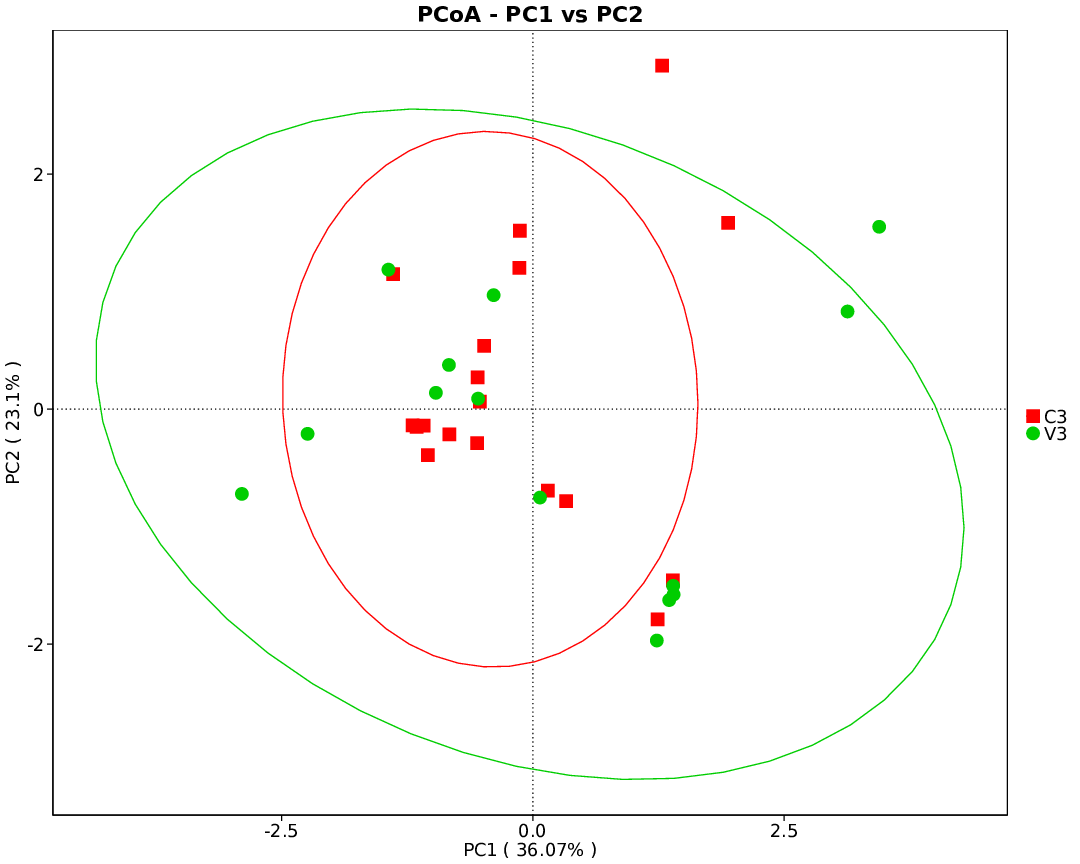


(C)


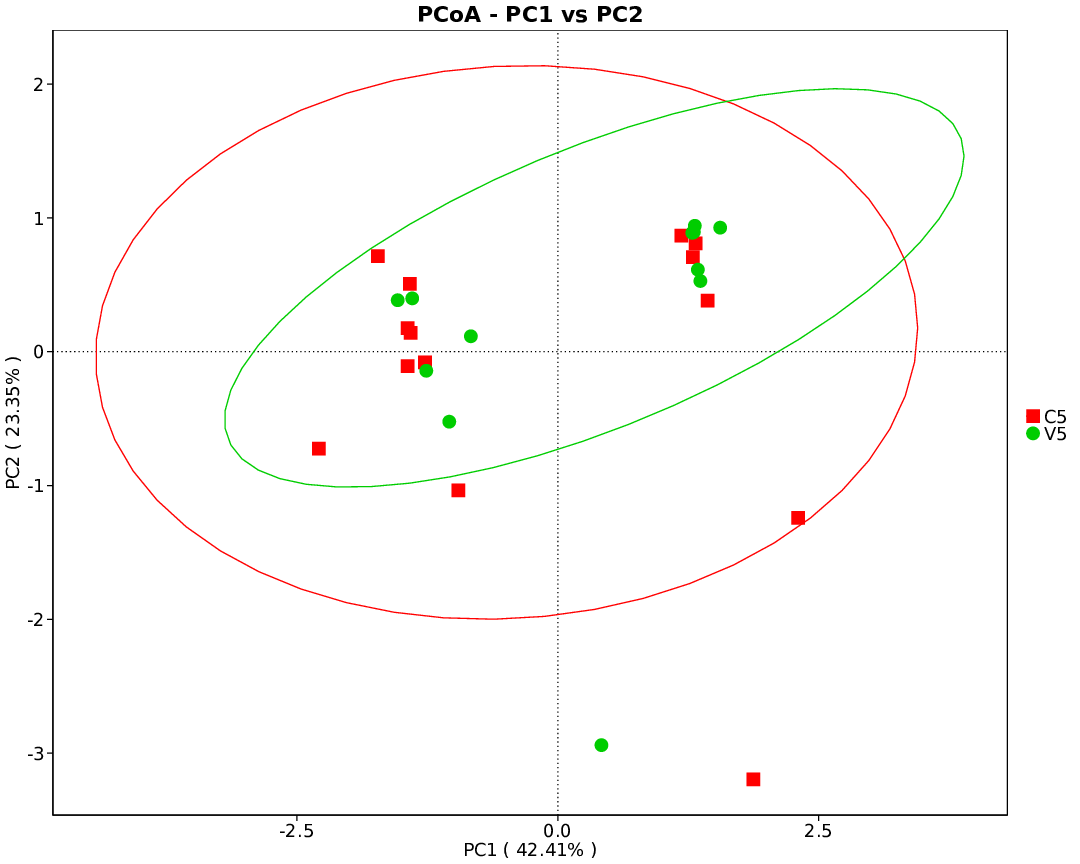


Supplementary Figure 25. PCoA analysis between the caesarean birth and vaginal delivery groups on days 1, 3, and 5. (A) PCoA analysis on day 1. (B) PCoA analysis on day 3. (C) PCoA analysis on day 5. C1, gut microbiota of the caesarean birth group on day 1; V1, gut microbiota of the vaginal delivery group on day 1; C3, gut microbiota of the caesarean birth group on day 3; V3, gut microbiota of the vaginal delivery group on day 3; C5, gut microbiota of the caesarean birth group on day 5; V5, gut microbiota of the vaginal delivery group on day 5.

(A) (B)


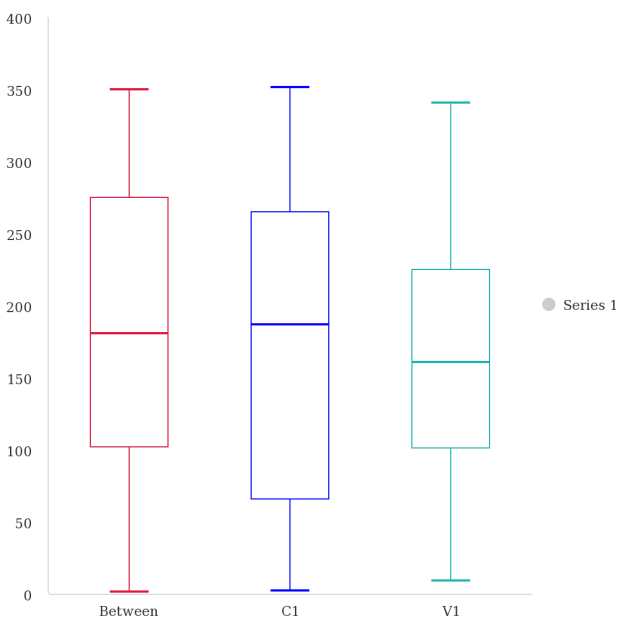

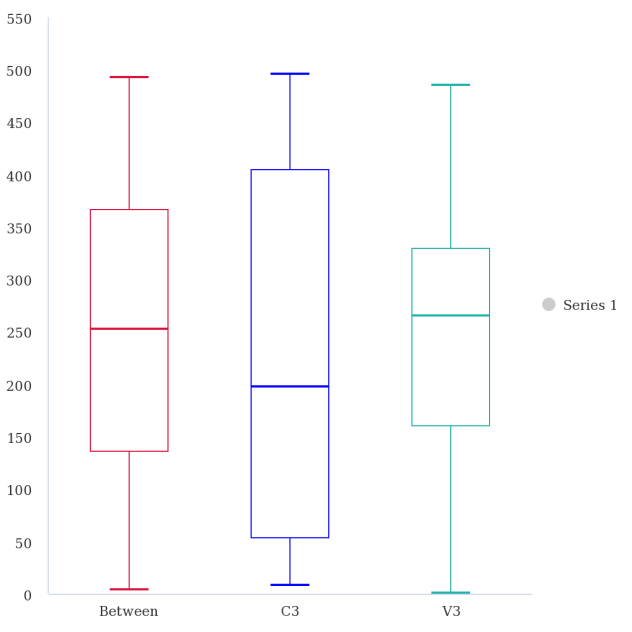


(C)


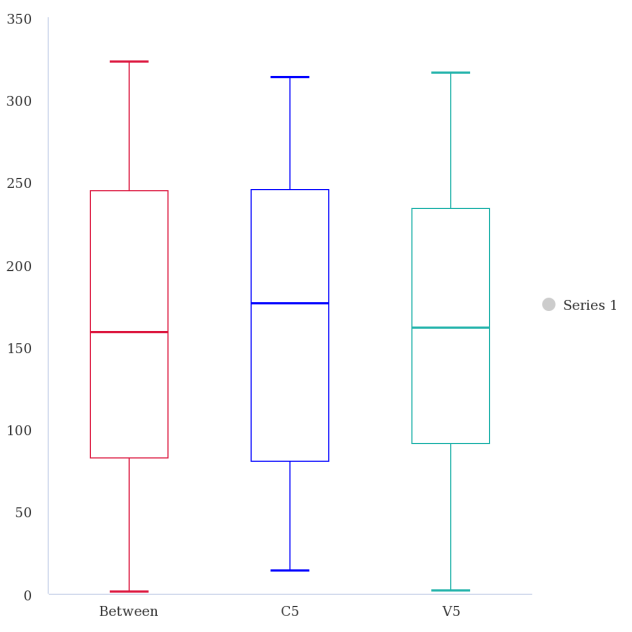


Supplementary Figure 26. Microbial differences between the caesarean birth and vaginal delivery groups on days 1, 3, and 5. (A) Microbial differences on day 1. (B) Microbial differences on day 3. (C) Microbial differences on day 5. C1, gut microbiota of the caesarean birth group on day 1; V1, gut microbiota of the vaginal delivery group on day 1; C3, gut microbiota of the caesarean birth group on day 3; V3, gut microbiota of the vaginal delivery group on day 3; C5, gut microbiota of the caesarean birth group on day 5; V5, gut microbiota of the vaginal delivery group on day 5.

(A) (B)


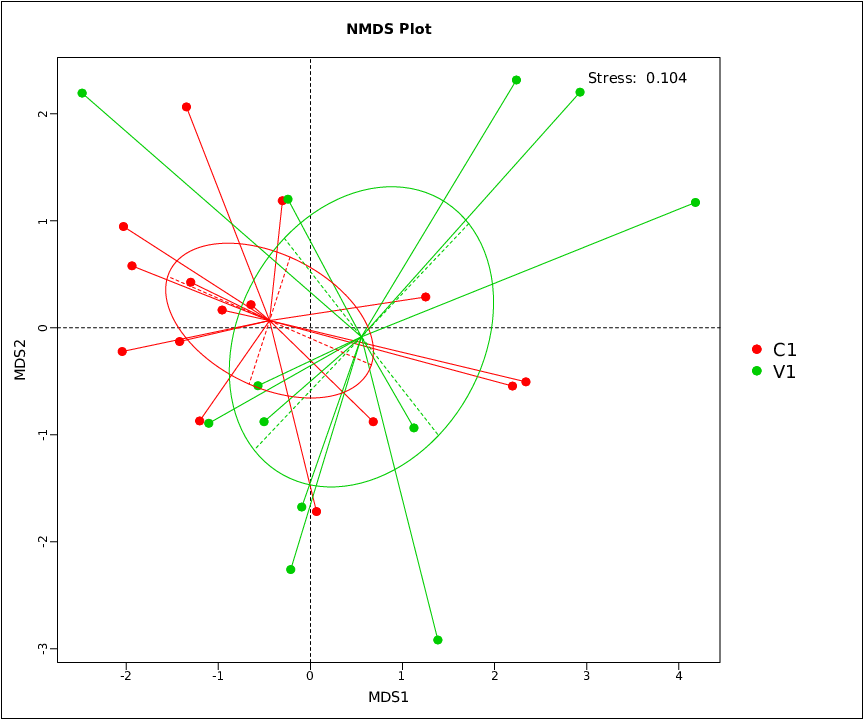

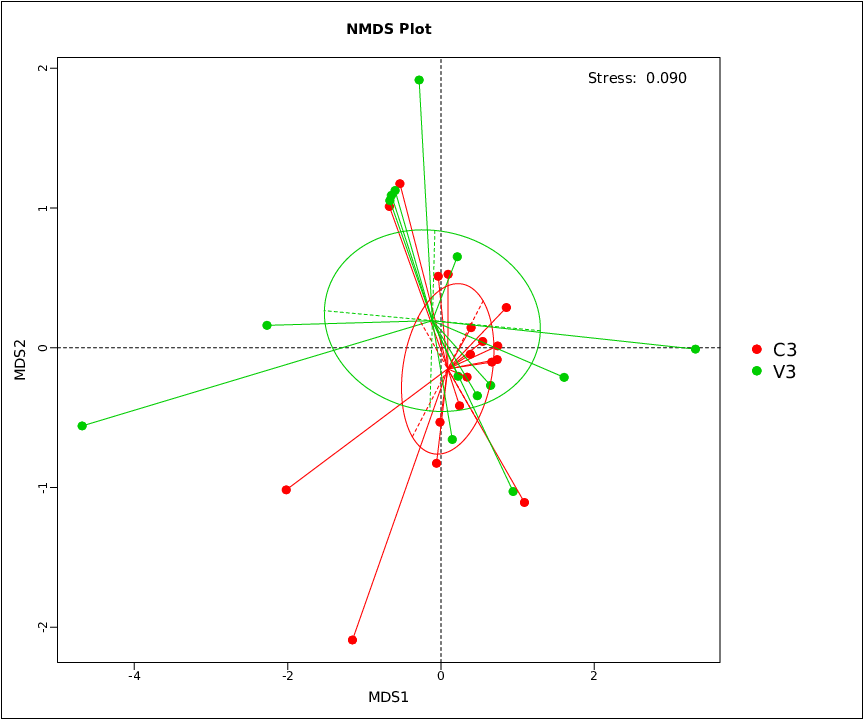


(C)


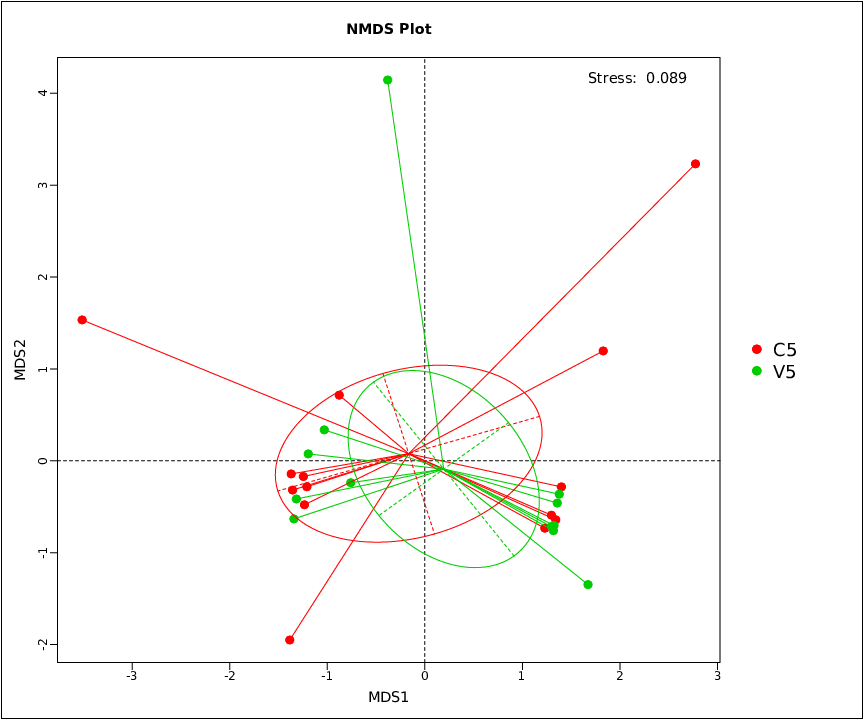


Supplementary Figure 27. NMDS analysis between the caesarean birth and vaginal delivery groups on days 1, 3, and 5. (A) NMDS analysis on day 1. (B) NMDS analysis on day 3. (C) NMDS analysis on day 5. C1, gut microbiota of the caesarean birth group on day 1; V1, gut microbiota of the vaginal delivery group on day 1; C3, gut microbiota of the caesarean birth group on day 3; V3, gut microbiota of the vaginal delivery group on day 3; C5, gut microbiota of the caesarean birth group on day 5; V5, gut microbiota of the vaginal delivery group on day 5.

(A)


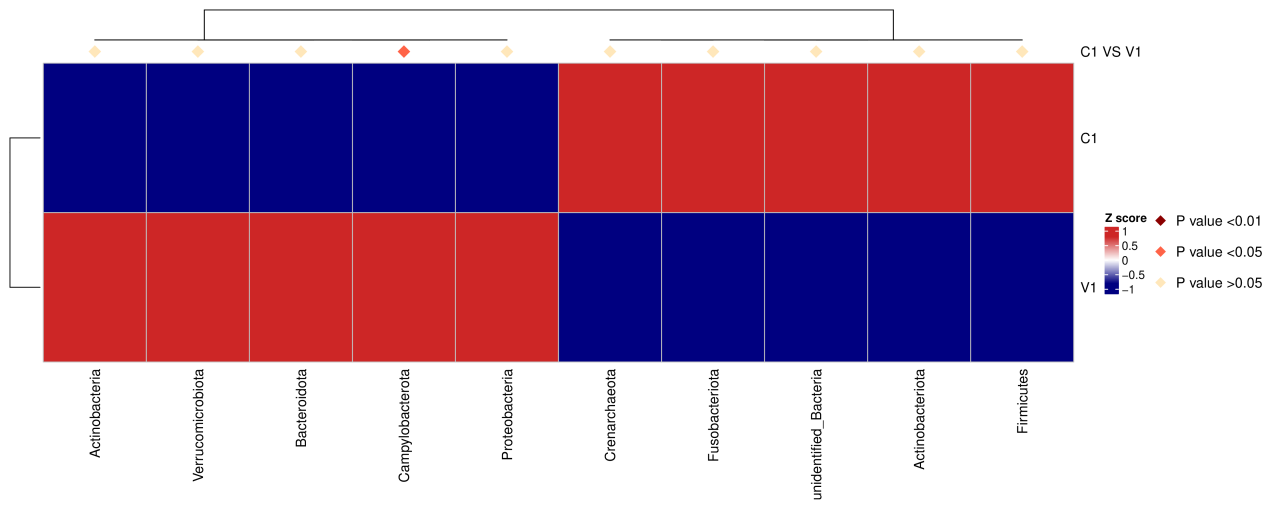


(B)


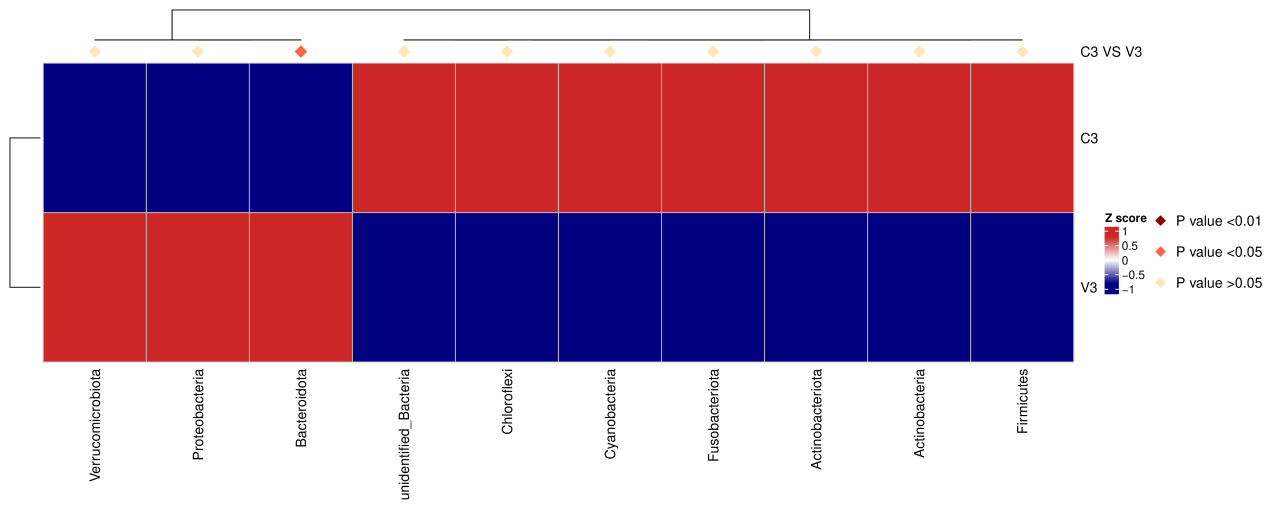


(C)


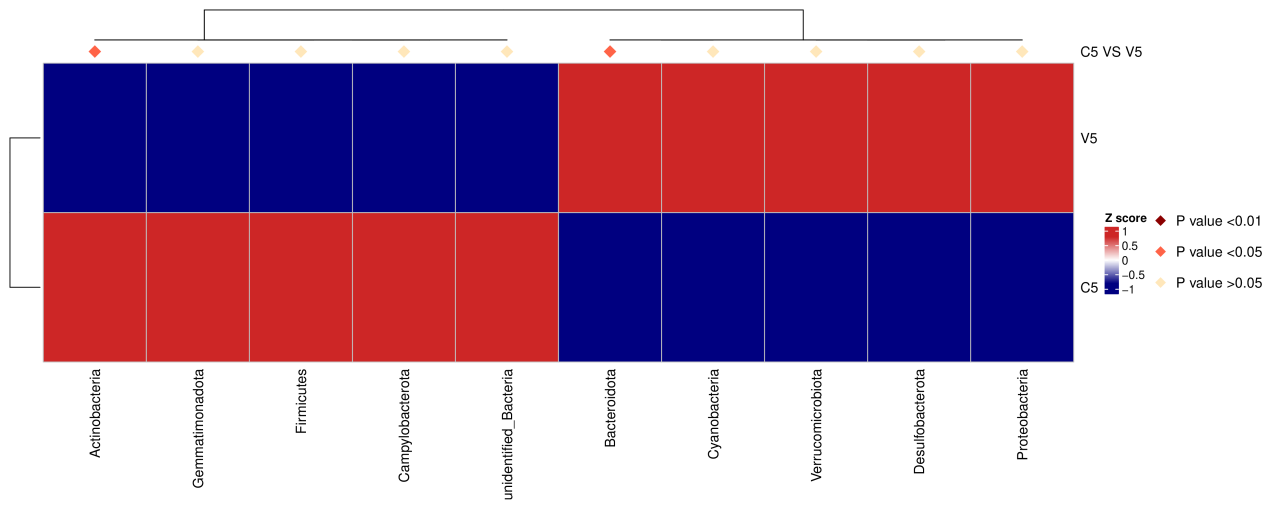


**Supplementary Figure 28.** Heatmap analysis between the caesarean birth and vaginal delivery groups at the phylum level on days 1, 3, and 5. (A) Heatmap analysis on day 1; (B) Heatmap analysis on day 3; (C) Heatmap analysis on day 5. C1, gut microbiota of the caesarean birth group on day 1; V1, gut microbiota of the vaginal delivery group on day 1; C3, gut microbiota of the caesarean birth group on day 3; V3, gut microbiota of the vaginal delivery group on day 3; C5, gut microbiota of the caesarean birth group on day 5; V5, gut microbiota of the vaginal delivery group on day 5.

(A)


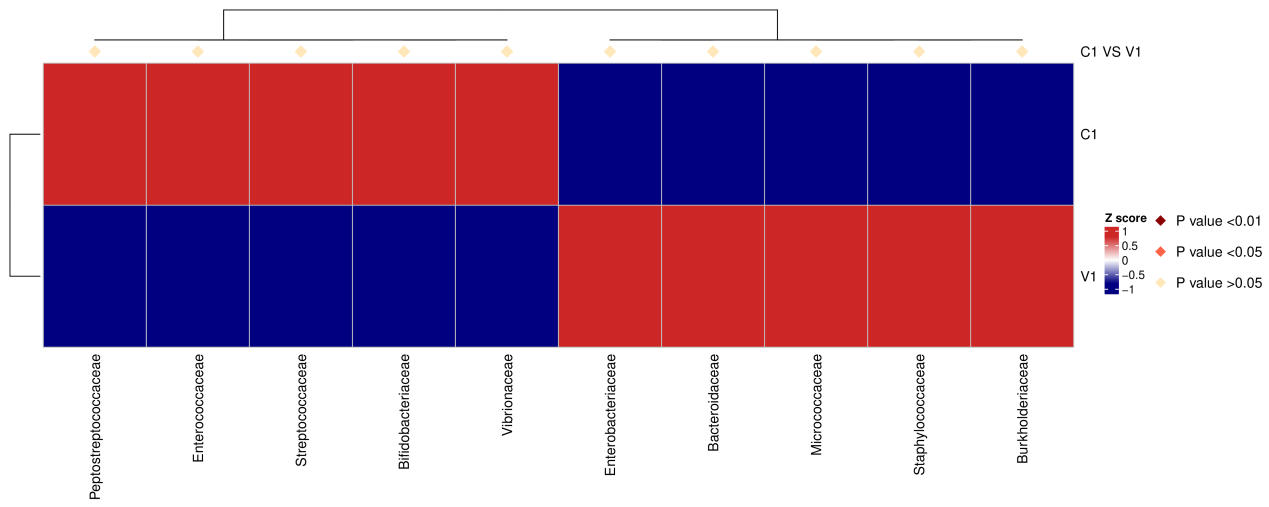


(B)


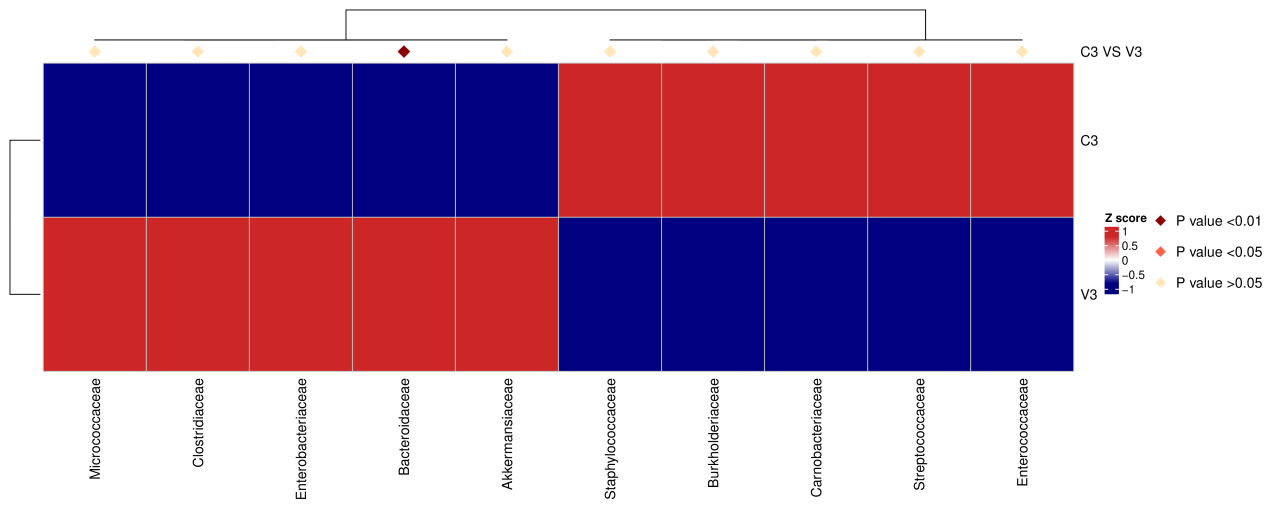


(C)


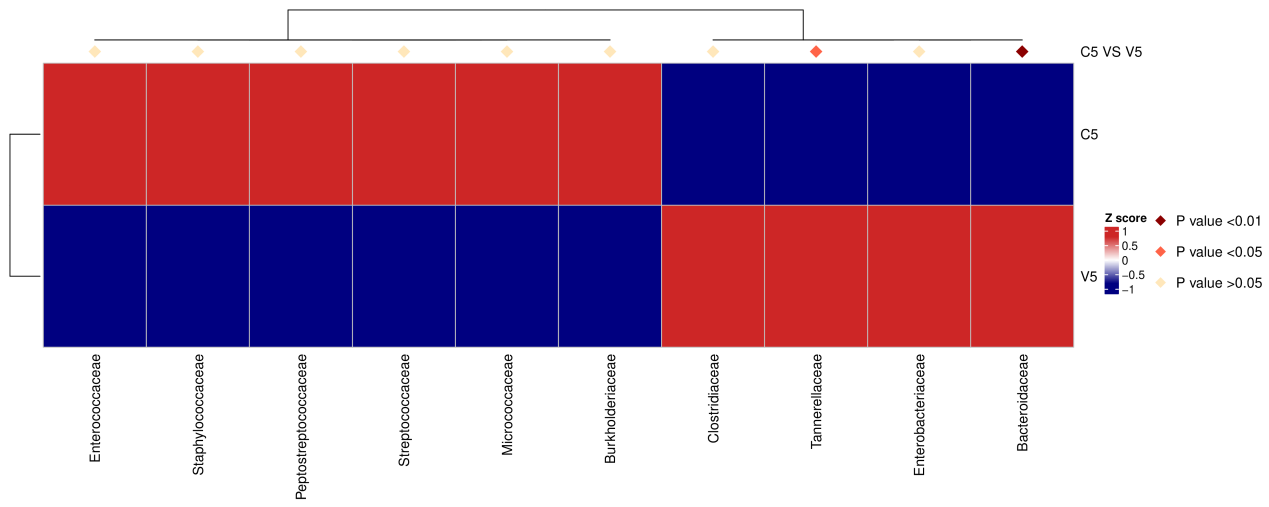


**Supplementary Figure 29.** Heatmap analysis between the caesarean birth and vaginal delivery groups at the family level on days 1, 3, and 5. (A) Heatmap analysis on day 1; (B) Heatmap analysis on day 3; (C) Heatmap analysis on day 5. C1, gut microbiota of the caesarean birth group on day 1; V1, gut microbiota of the vaginal delivery group on day 1; C3, gut microbiota of the caesarean birth group on day 3; V3, gut microbiota of the vaginal delivery group on day 3; C5, gut microbiota of the caesarean birth group on day 5; V5, gut microbiota of the vaginal delivery group on day 5.

(A)


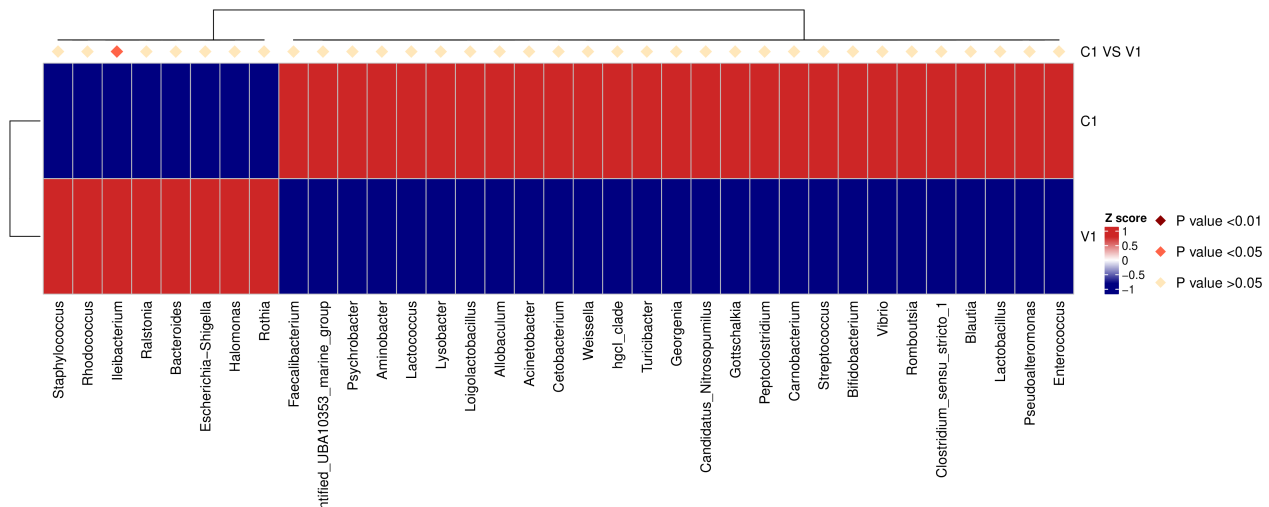


(B)


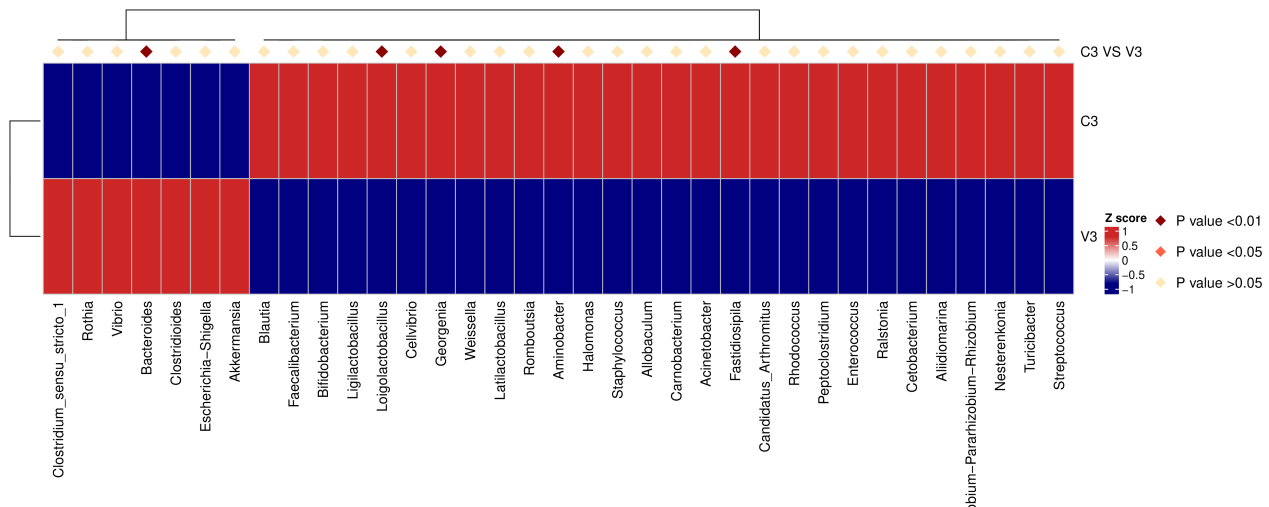


(C)


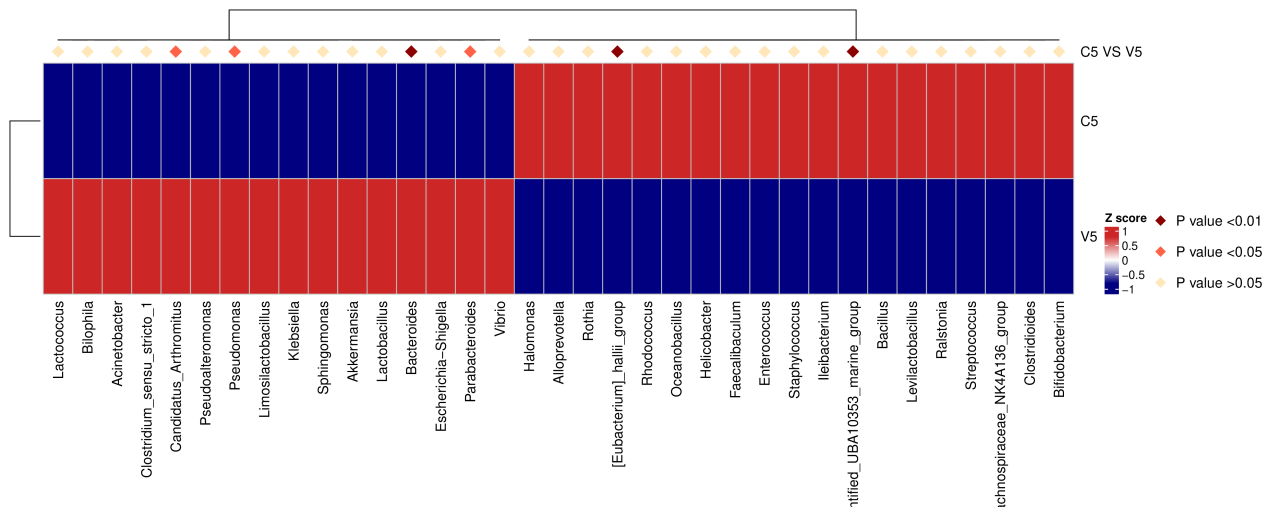


**Supplementary Figure 30.** Heatmap analysis between the caesarean birth and vaginal delivery groups at the genus level on days 1, 3, and 5. (A) Heatmap analysis on day 1; (B) Heatmap analysis on day 3; (C) Heatmap analysis on day 5. C1, gut microbiota of the caesarean birth group on day 1; V1, gut microbiota of the vaginal delivery group on day 1; C3, gut microbiota of the caesarean birth group on day 3; V3, gut microbiota of the vaginal delivery group on day 3; C5, gut microbiota of the caesarean birth group on day 5; V5, gut microbiota of the vaginal delivery group on day 5.

.

(A)


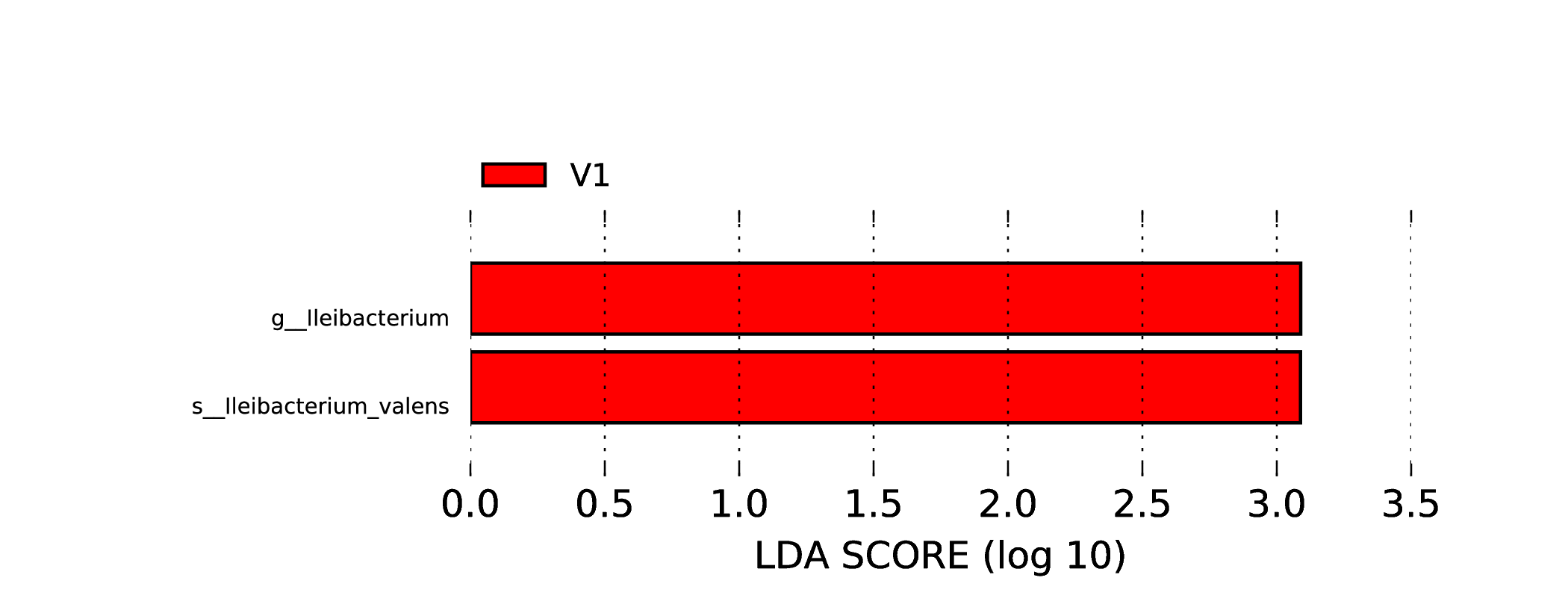


(B)


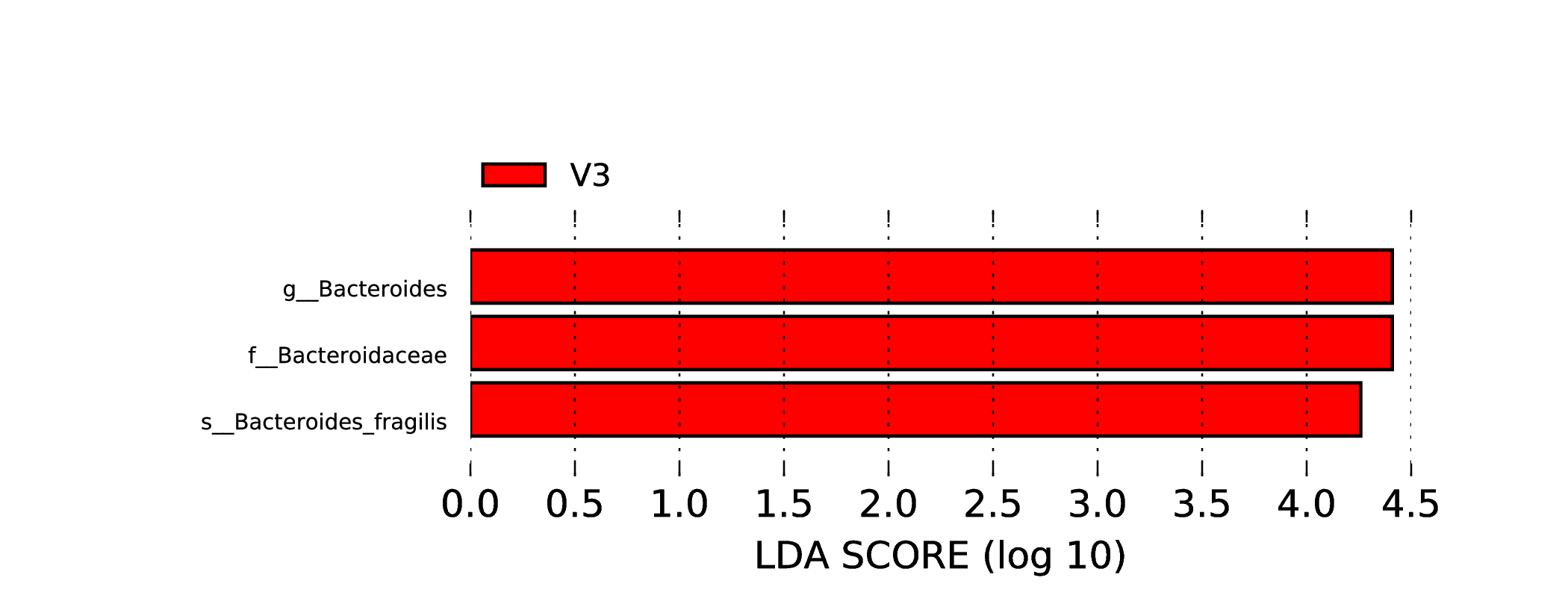


(C)


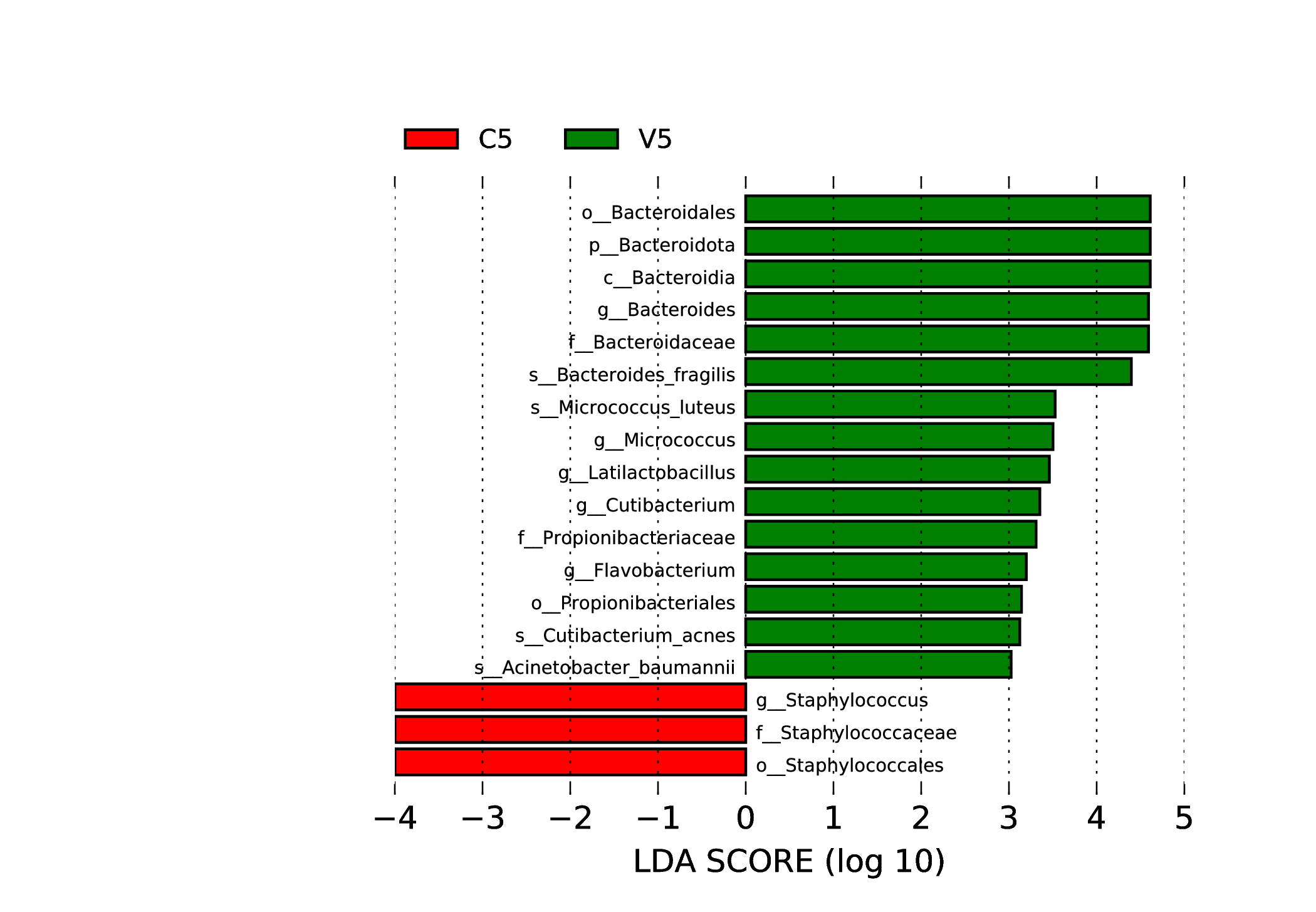


**Supplementary Figure 31.** LEfSe comparison between the caesarean birth and vaginal delivery groups. (A) LDA score histogram of differential microbiota of the two groups on day 1. (B) LDA score histogram of differential microbiota of the two groups on day 3. (C) LDA score histogram of differential microbiota of the two groups on day 5. V1, gut microbiota of the vaginal delivery group on day 1; V3, gut microbiota of the vaginal delivery group on day 3; V5, gut microbiota of the vaginal delivery group on day 5.

1. (B)

**
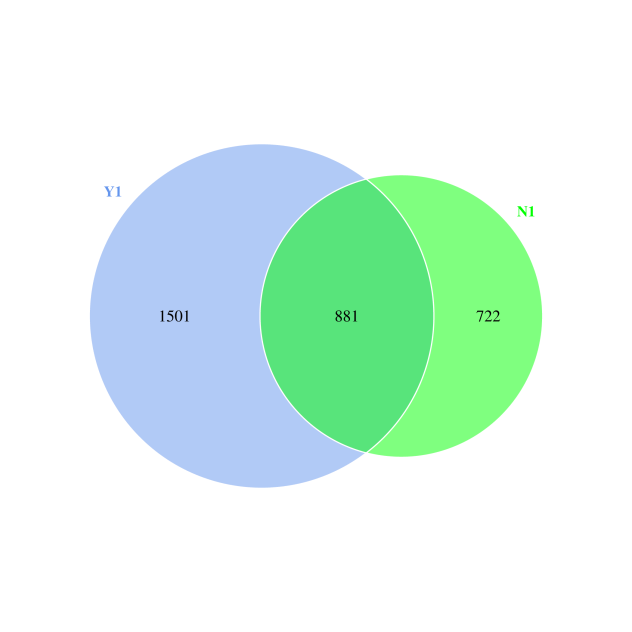

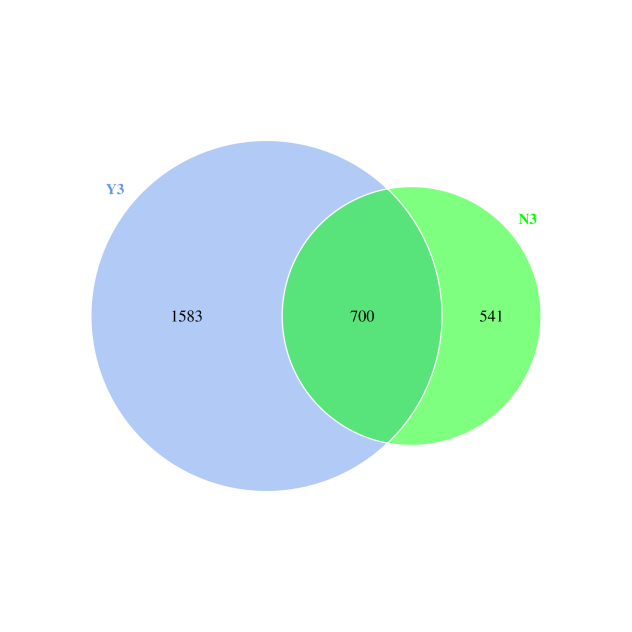
**

(C)

**
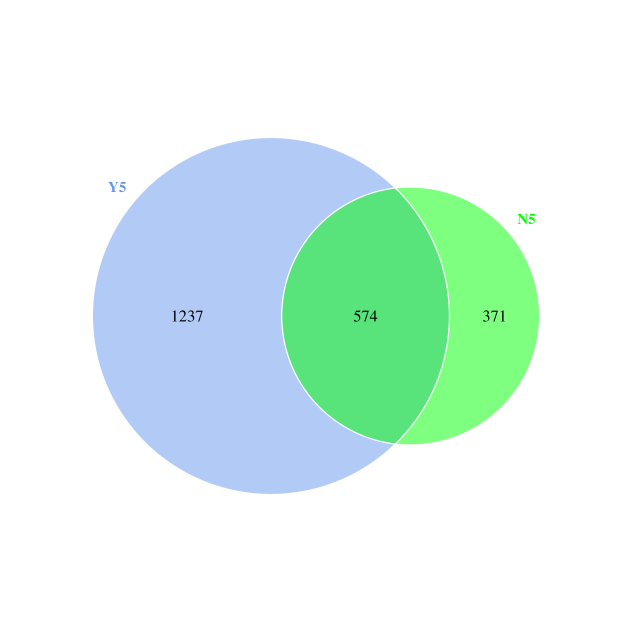
**

Supplementary Figure 32. Venn diagrams of the group with antibiotics and the group without antibiotics on days 1, 3, and 5. (A) Venn diagram on day 1; (B) Venn diagram on day 3; (C) Venn diagram on day 5. N1, gut microbiota of the group without antibiotics on day 1; Y1, gut microbiota of the group with antibiotics on day 1; N3, gut microbiota of the group without antibiotics on day 3; Y3, gut microbiota of the group with antibiotics on day 3; N5, gut microbiota of the group without antibiotics on day 5; Y5, gut microbiota of the group with antibiotics on day 5.

(A) (B)


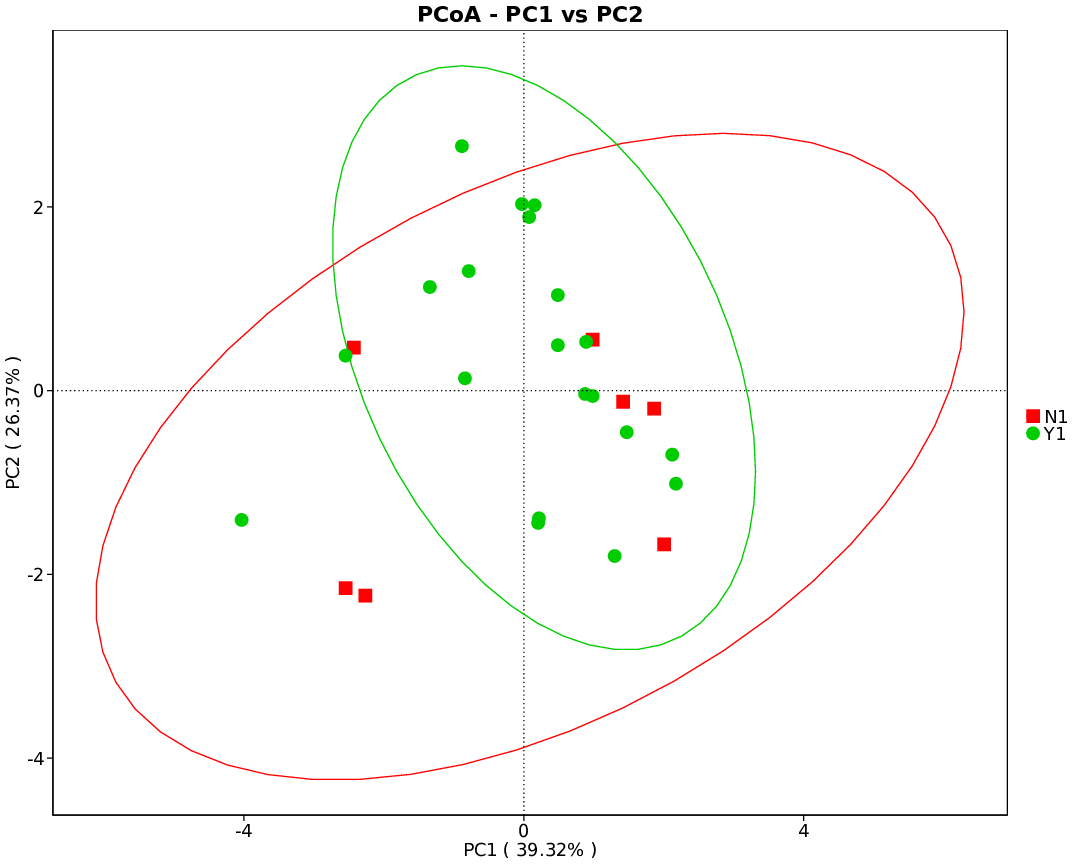

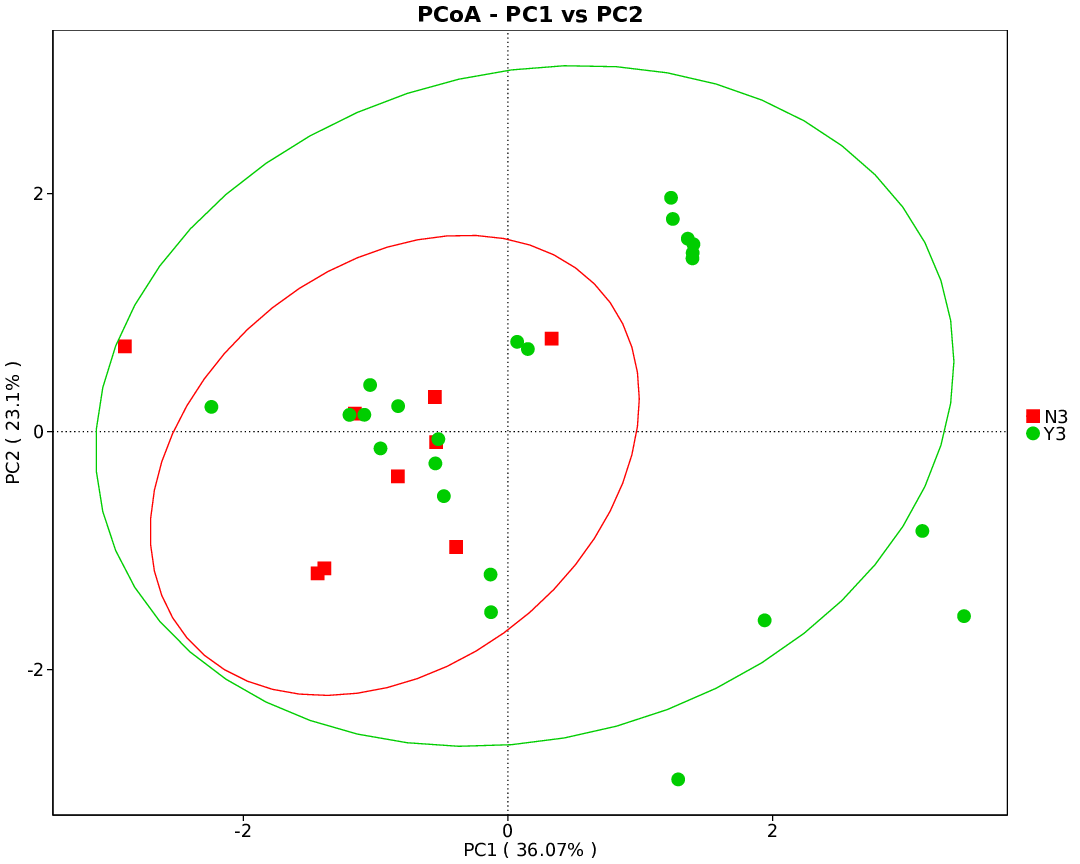


(C)


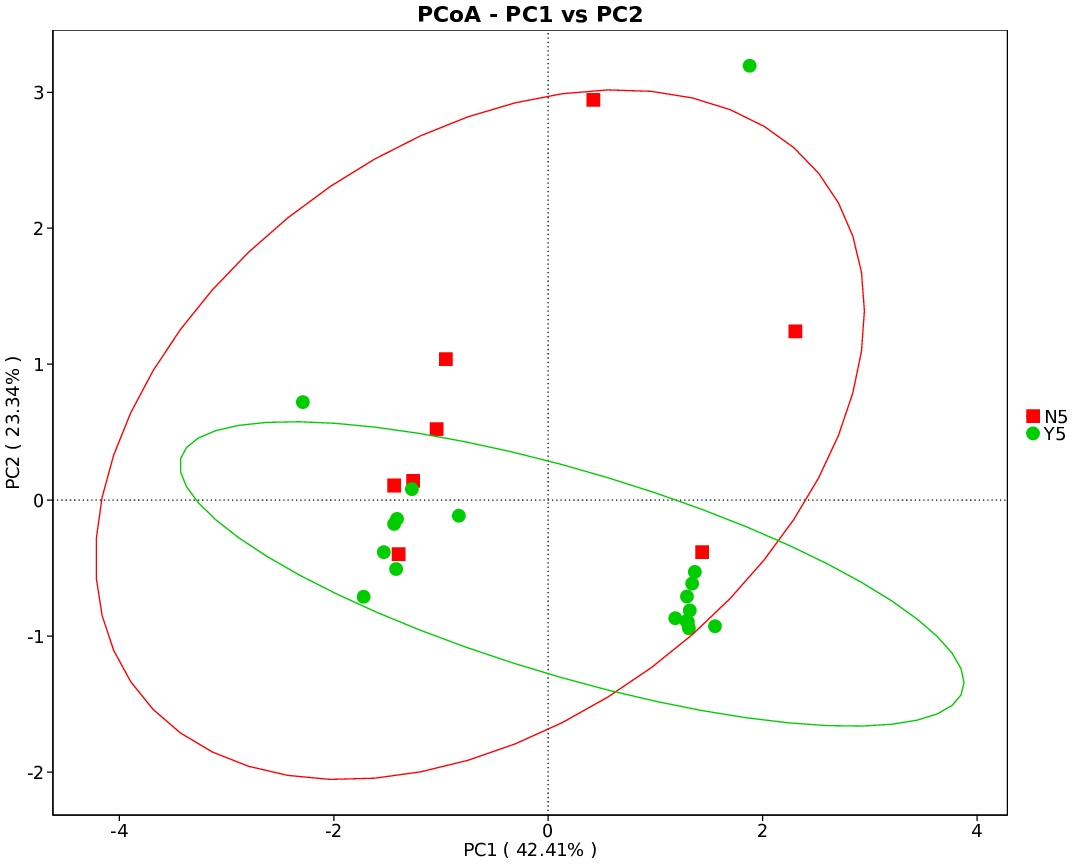


Supplementary Figure 33. PCoA analysis between the group with antibiotics and the group without antibiotics on days 1, 3, and 5. (A) PCoA analysis on day 1. (B) PCoA analysis on day 3. (C) PCoA analysis on day 5. N1, gut microbiota of the group without antibiotics on day 1; Y1, gut microbiota of the group with antibiotics on day 1; N3, gut microbiota of the group without antibiotics on day 3; Y3, gut microbiota of the group with antibiotics on day 3; N5, gut microbiota of the group without antibiotics on day 5; Y5, gut microbiota of the group with antibiotics on day 5.

(A) (B)


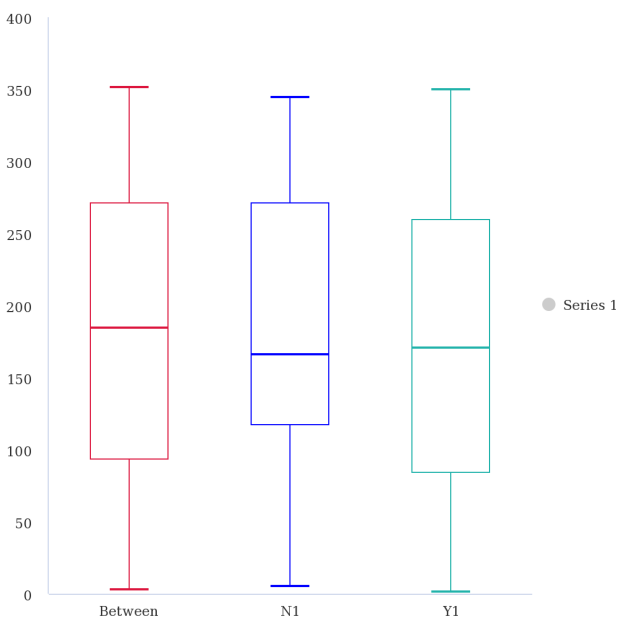

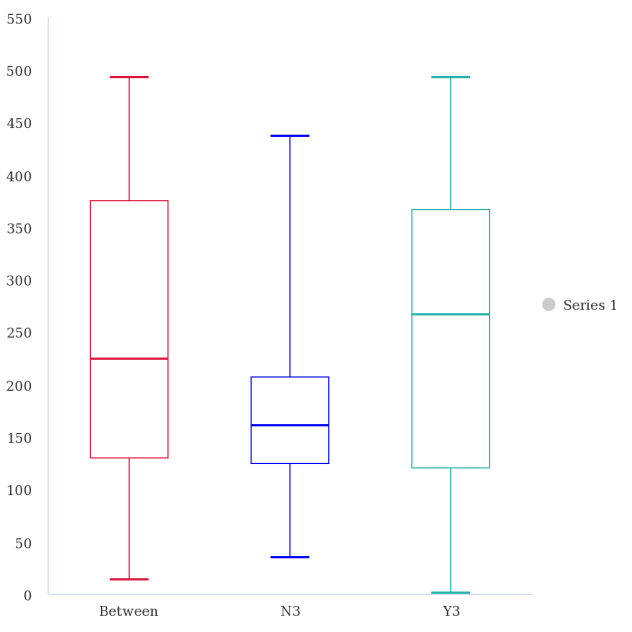


(C)


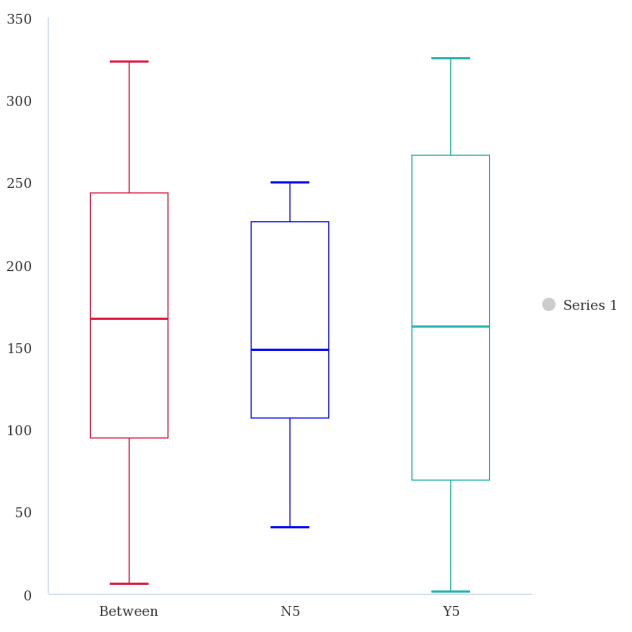


**Supplementary Figure 34.** Microbial differences between the group with antibiotics and the group without antibiotics on days 1, 3, and 5. (A) Microbial differences on day 1. (B) Microbial differences on day 3. (C) Microbial differences on day 5. N1, gut microbiota of the group without antibiotics on day 1; Y1, gut microbiota of the group with antibiotics on day 1; N3, gut microbiota of the group without antibiotics on day 3; Y3, gut microbiota of the group with antibiotics on day 3; N5, gut microbiota of the group without antibiotics on day 5; Y5, gut microbiota of the group with antibiotics on day 5.

(A) (B)


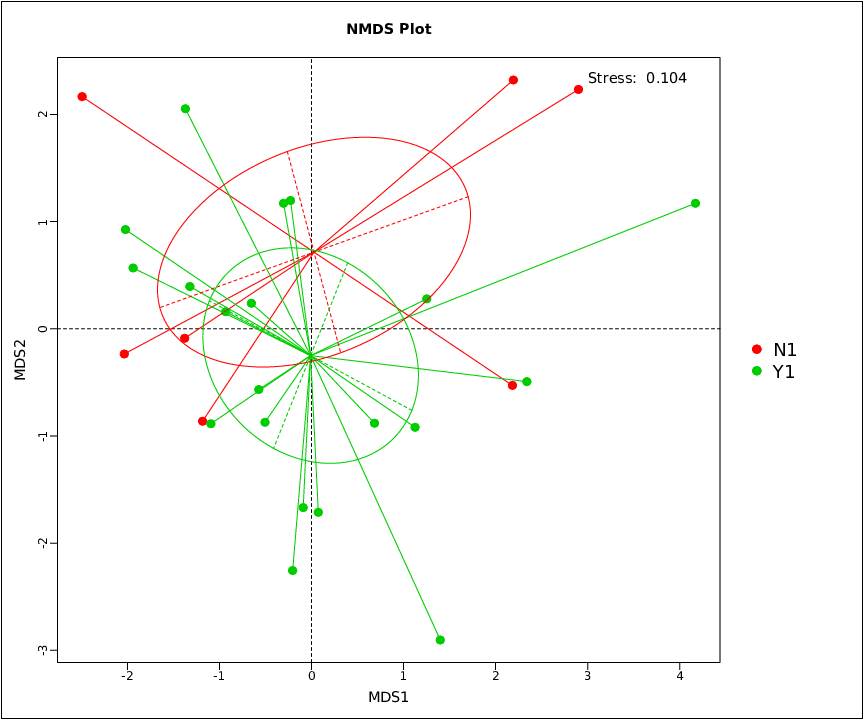

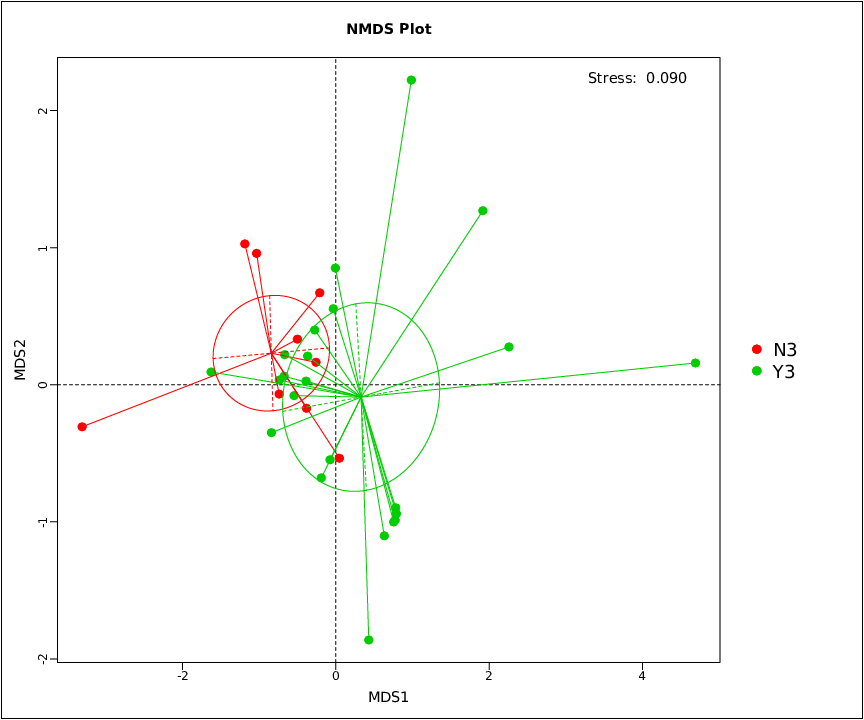


(C)


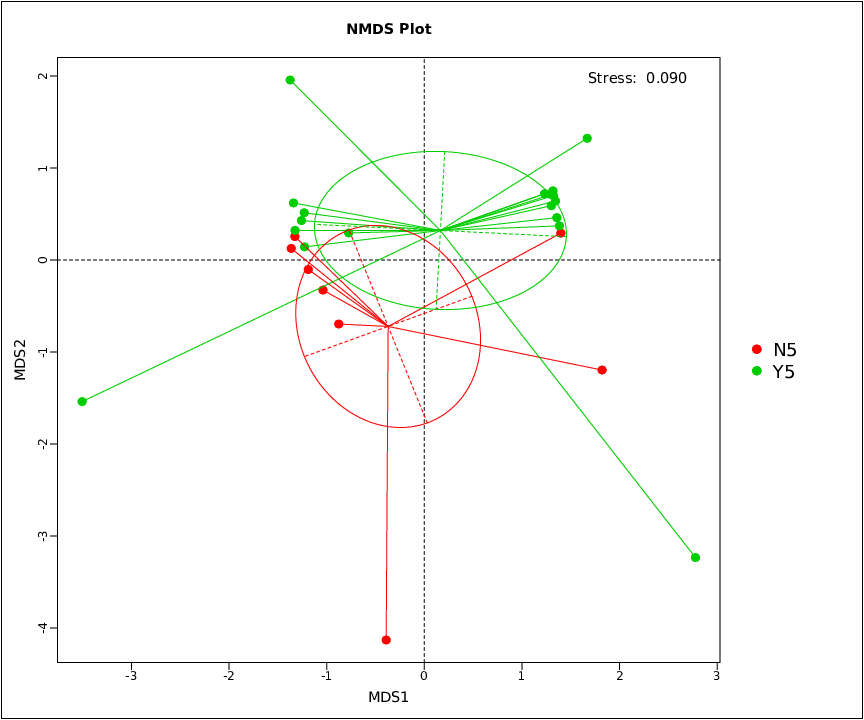


Supplementary Figure 35. NMDS analysis between the group with antibiotics and the group without antibiotics on days 1, 3, and 5. (A) NMDS analysis on day 1. (B) NMDS analysis on day 3. (C) NMDS analysis on day 5. N1, gut microbiota of the group without antibiotics on day 1; Y1, gut microbiota of the group with antibiotics on day 1; N3, gut microbiota of the group without antibiotics on day 3; Y3, gut microbiota of the group with antibiotics on day 3; N5, gut microbiota of the group without antibiotics on day 5; Y5, gut microbiota of the group with antibiotics on day 5.

(A)


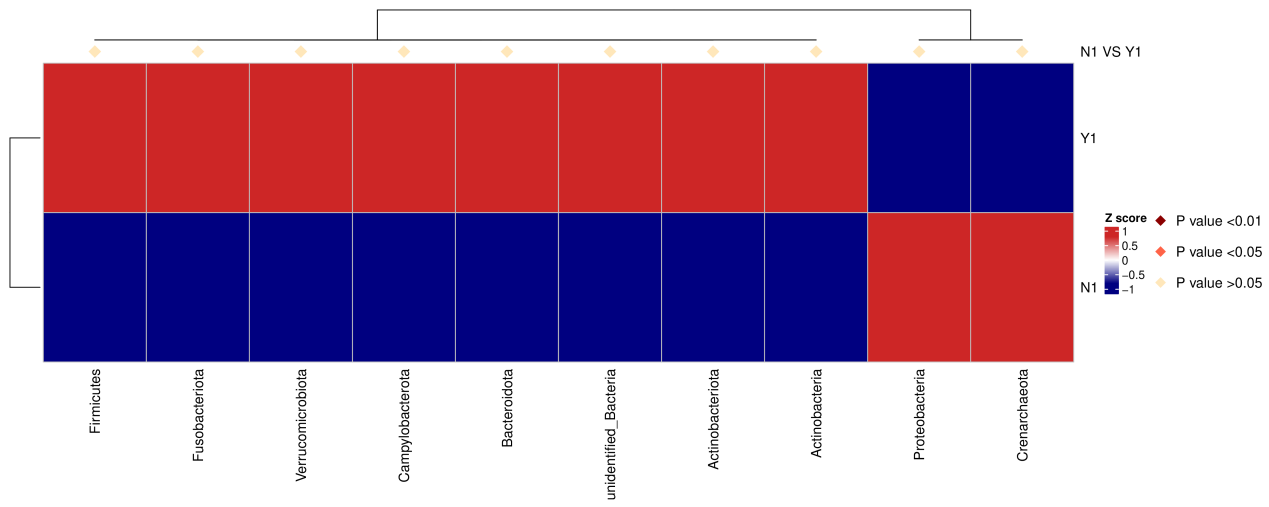


(B)


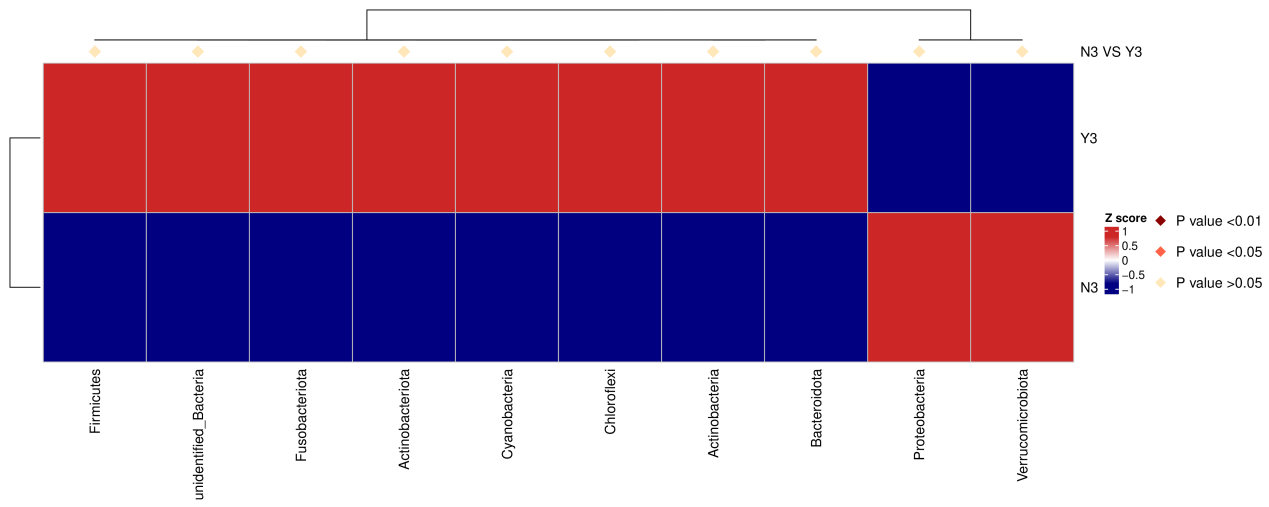


(C)


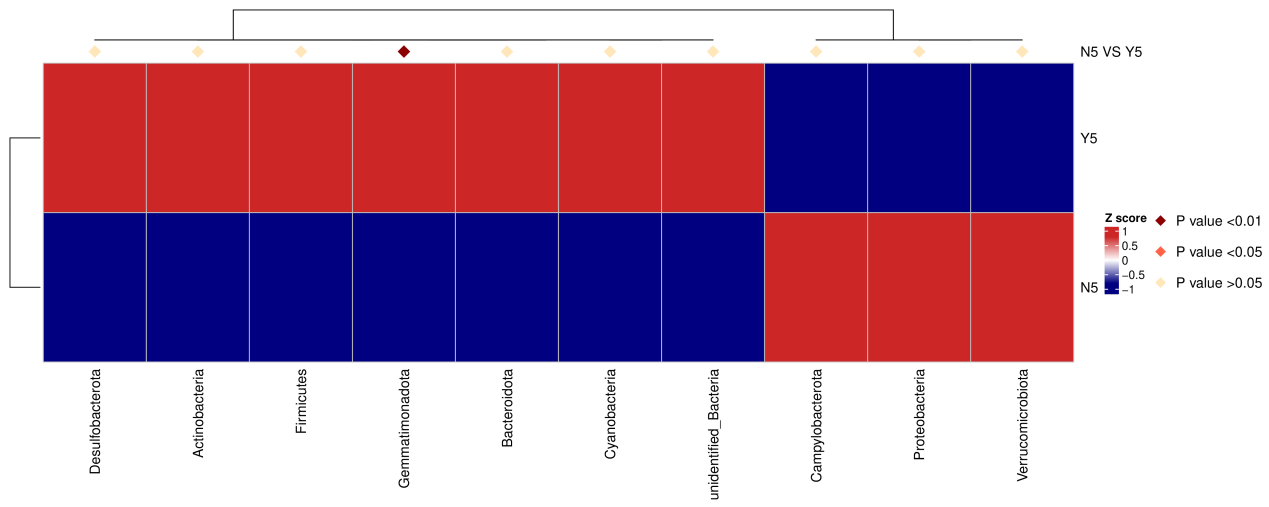


**Supplementary Figure 36.** Heatmap analysis between the group with antibiotics and the group without antibiotics at the phylum level on days 1, 3, and 5. (A) Heatmap analysis on day 1; (B) Heatmap analysis on day 3; (C) Heatmap analysis on day 5. N1, gut microbiota of the group without antibiotics on day 1; Y1, gut microbiota of the group with antibiotics on day 1; N3, gut microbiota of the group without antibiotics on day 3; Y3, gut microbiota of the group with antibiotics on day 3; N5, gut microbiota of the group without antibiotics on day 5; Y5, gut microbiota of the group with antibiotics on day 5.

(A)


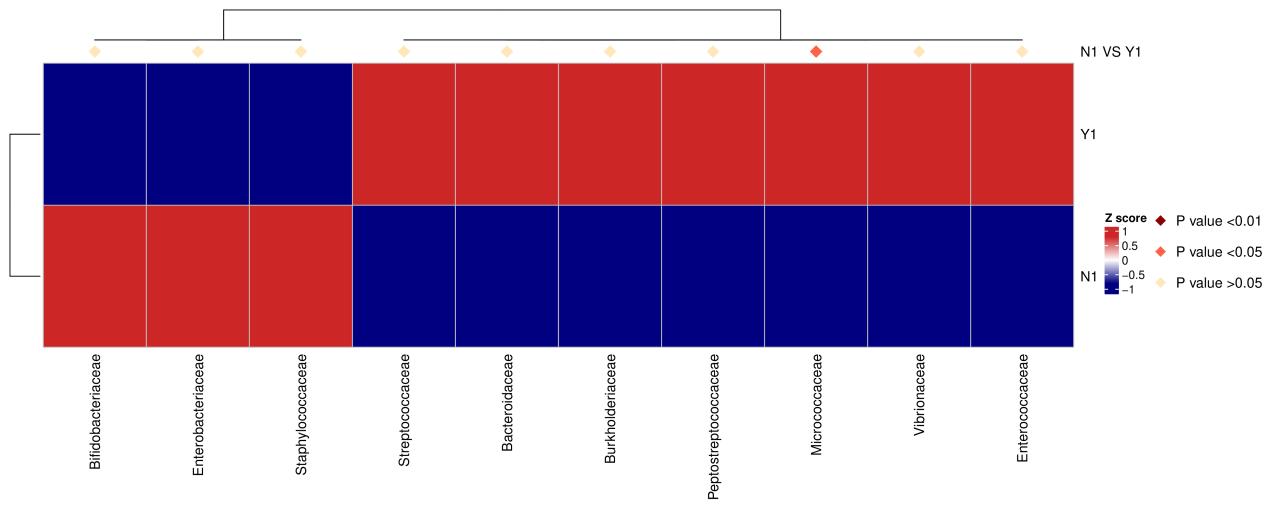


(B)


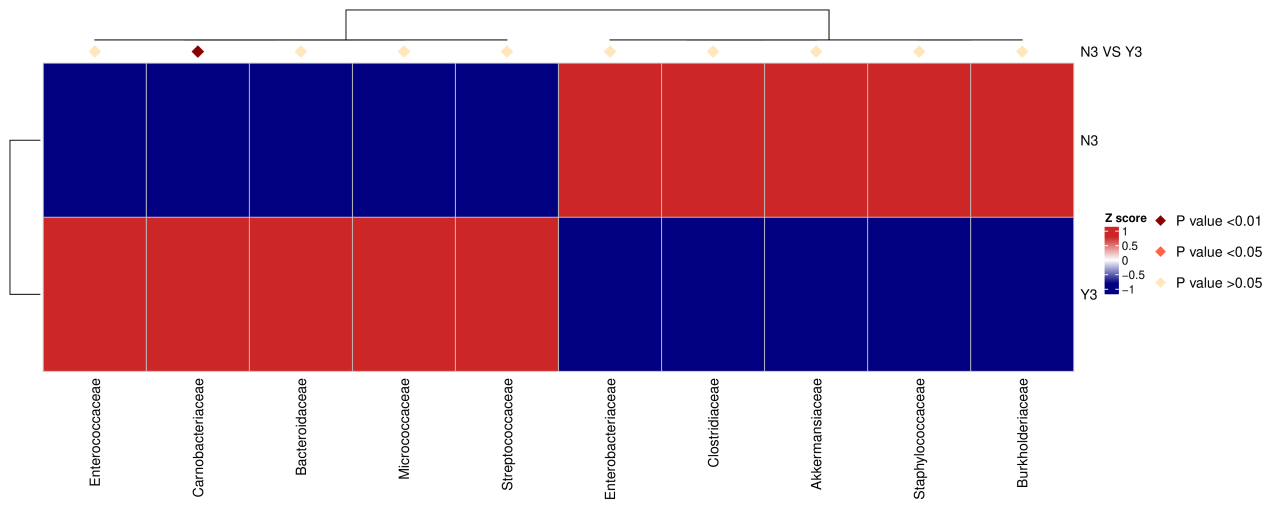


(C)


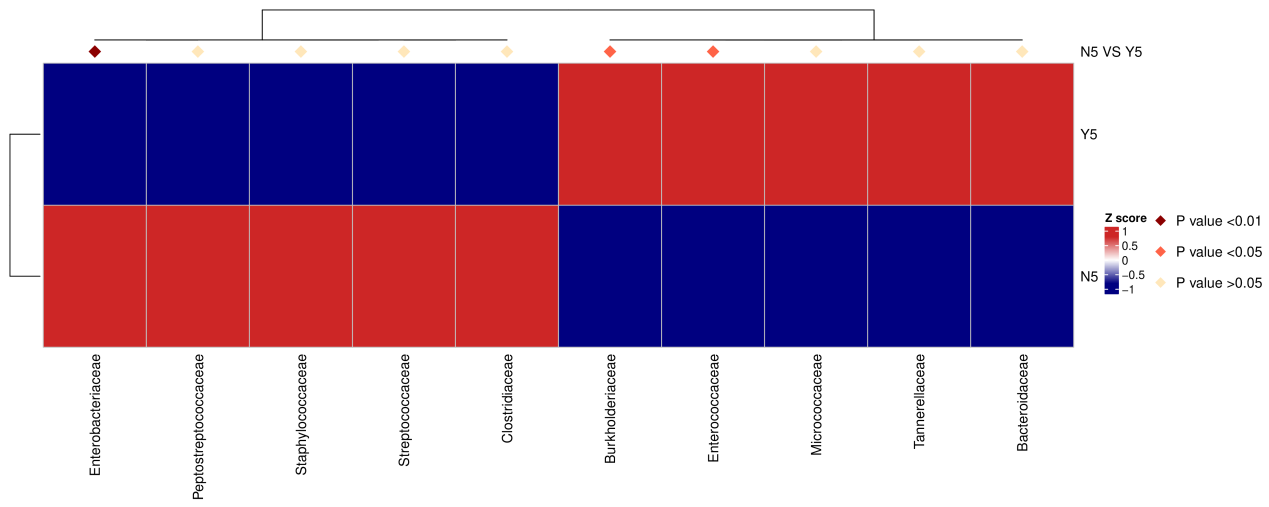


Supplementary Figure 37. Heatmap analysis between the group with antibiotics and the group without antibiotics at the family level on days 1, 3, and 5. (A) Heatmap analysis on day 1; (B) Heatmap analysis on day 3; (C) Heatmap analysis on day 5. N1, gut microbiota of the group without antibiotics on day 1; Y1, gut microbiota of the group with antibiotics on day 1; N3, gut microbiota of the group without antibiotics on day 3; Y3, gut microbiota of the group with antibiotics on day 3; N5, gut microbiota of the group without antibiotics on day 5; Y5, gut microbiota of the group with antibiotics on day 5.

(A)


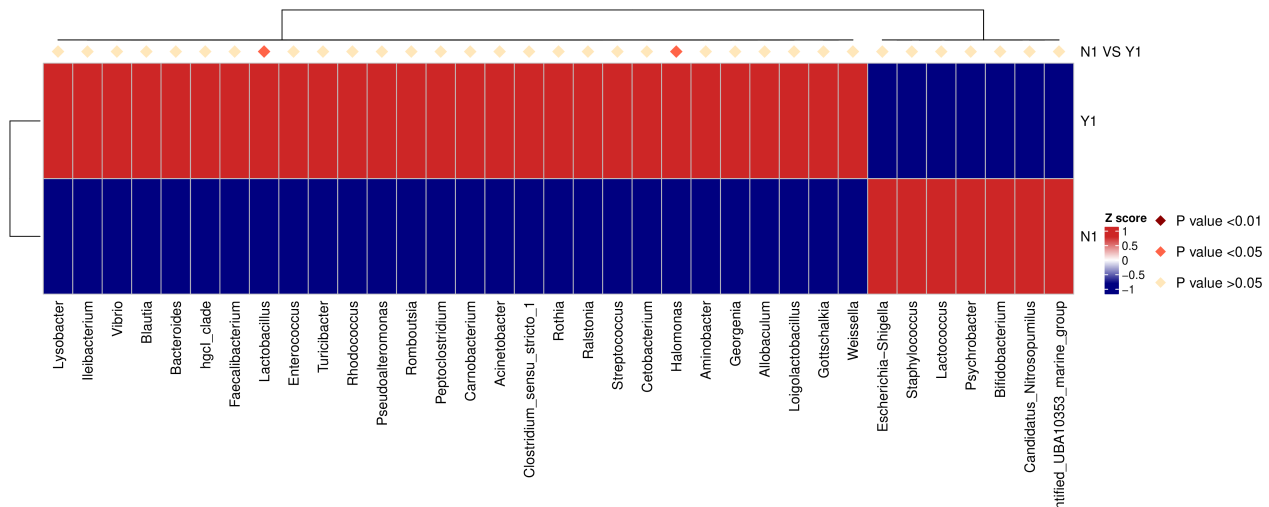


(B)


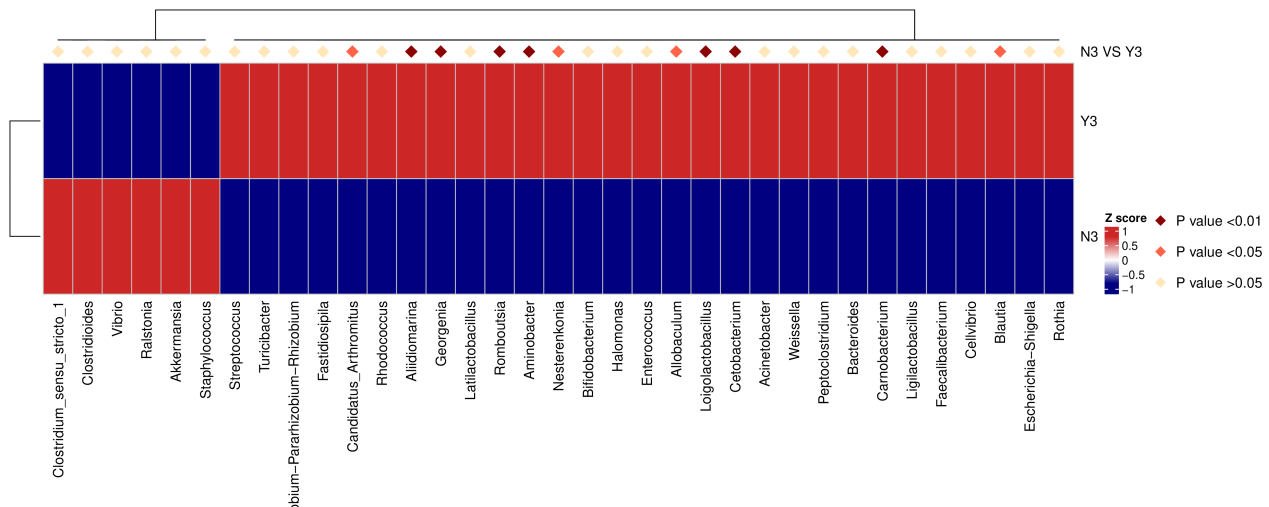


(C)


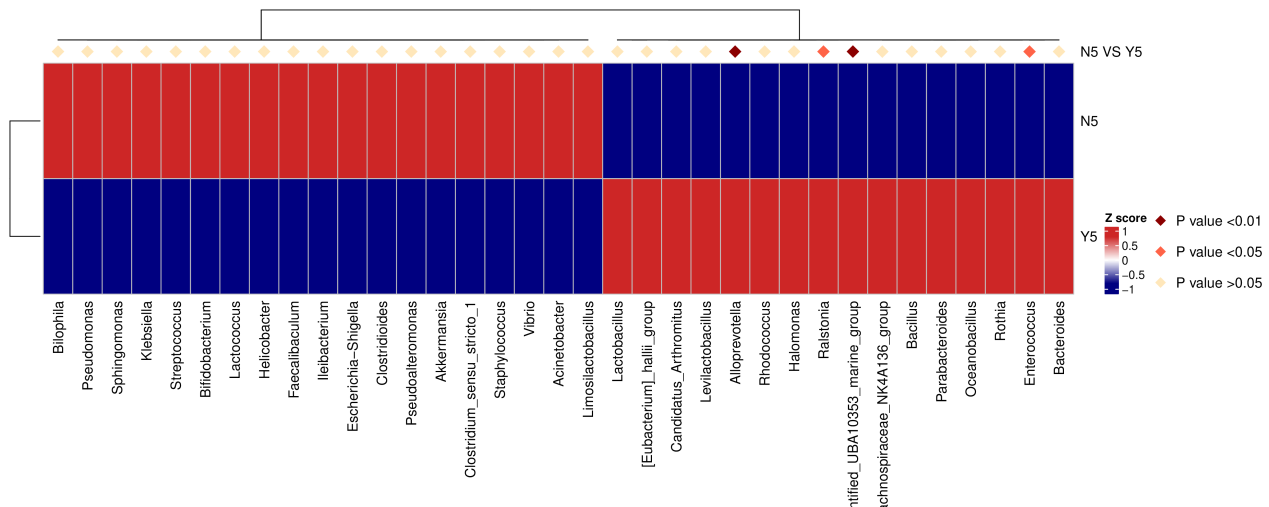


Supplementary Figure 38. Heatmap analysis between the group with antibiotics and the group without antibiotics at the genus level on days 1, 3, and 5. (A) Heatmap analysis on day 1; (B) Heatmap analysis on day 3; (C) Heatmap analysis on day 5. N1, gut microbiota of the group without antibiotics on day 1; Y1, gut microbiota of the group with antibiotics on day 1; N3, gut microbiota of the group without antibiotics on day 3; Y3, gut microbiota of the group with antibiotics on day 3; N5, gut microbiota of the group without antibiotics on day 5; Y5, gut microbiota of the group with antibiotics on day 5.

(A)

(B)

(C)

Supplementary Figure 39. LEfSe comparison between the group with antibiotics and the group without antibiotics. (A) LDA score histogram of differential microbiota of the two groups on day 1. (B) LDA score histogram of differential microbiota of the two groups on day 3. (C) LDA score histogram of differential microbiota of the two groups on day 5. N1, gut microbiota of the group without antibiotics on day 1; Y1, gut microbiota of the group with antibiotics on day 1; N3, gut microbiota of the group without antibiotics on day 3; Y3, gut microbiota of the group with antibiotics on day 3; N5, gut microbiota of the group without antibiotics on day 5; Y5, gut microbiota of the group with antibiotics on day 5.

**Supplementary Tables**

**Supplementary Table 1.** Comparison of alpha diversity of gut microbiota in the SGA group on days 1, 3, 5, and 7.

| **Days** | **Observed_species** | **Chao1** | **ACE** | **Shannon** | **Simpson** |
| --- | --- | --- | --- | --- | --- |
| D1 | 207 (78, 362) | 302.1 (98.9, 446) | 318.5 (105.9, 497.0) | 1.94 (1.35, 2.68) | 0.55 (0.34, 0.66) |
| D3 | 238 (114.8, 282.3) | 320.8 (196.0, 387.8) | 346.2 (195.7, 404.4) | 1.78 (0.89, 2.82) | 0.47 (0.20, 0.67) |
| D5 | 197 (87.3, 280) | 278.6 (119.2, 391.6) | 292.3 (128.2, 438.2) | 1.61 (1.06, 2.78) | 0.47 (0.26, 0.73) |
| D7 | 115 (70, 244.5) | 121.7 (84.8, 319.8) | 126.1 (91.7, 338.7) | 1.61 (0.76, 3.03) | 0.47 (0.19, 0.75) |
| H | 0.06 | 0.01 | 0.07 | 0.20 | 0.58 |
| *P* | 0.808 | 0.915 | 0.796 | 0.654 | 0.447 |

**Supplementary Table 2.** Comparison of alpha diversity between the SGA and AGA groups on days 1, 3, 5 and 7.

| **Days** | **Alpha index** | **SGA group** | **AGA group** | **z** | ***P*** |
| --- | --- | --- | --- | --- | --- |
| D1 | Observed_species | 207 (78, 362) | 335 (231, 594.5) | 3.02 | 0.003 |
|  | Chao1 | 302.1 (98.9, 446.0) | 469.09 (320.5, 789.0) | 3.14 | 0.002 |
|  | ACE | 318.5 (105.9, 497.0) | 516.9 (351.2, 829.2) | 3.08 | 0.002 |
|  | Shannon | 1.94 (1.35, 2.68) | 2.83 (1.89, 3.48) | 2.78 | 0.006 |
|  | Simpson | 0.55 (0.34, 0.66) | 0.70 (0.45, 0.81) | 2.21 | 0.027 |
| D3 | Observed_species | 238 (114.8, 282.3) | 306 (195, 419) | 2.48 | 0.013 |
|  | Chao1 | 320.8 (196.0, 387.8) | 415.1 (264.1, 624.1) | 2.53 | 0.012 |
|  | ACE | 346.2 (195.7, 404.4) | 444.6 (279.2, 653.6) | 2.63 | 0.009 |
|  | Shannon | 1.78 (0.89, 2.82) | 2.24 (1.61, 3.02) | 1.52 | 0.129 |
|  | Simpson | 0.47 (0.20, 0.67) | 0.57 (0.41, 0.69) | 1.29 | 0.196 |
| D5 | Observed_species | 197 (87.3, 280) | 280 (207, 331) | 2.941 | 0.003 |
|  | Chao1 | 278.6 (119.2, 391.6) | 398.8 (296.2, 494.3) | 2.932 | 0.003 |
|  | ACE | 292.3 (128.2, 438.2) | 434.7 (306.1, 508.3) | 2.761 | 0.006 |
|  | Shannon | 1.61 (1.06, 2.78) | 2.33 (1.88, 2.70) | 1.619 | 0.105 |
|  | Simpson | 0.47 (0.26, 0.73) | 0.62 (0.50, 0.68) | 1.2 | 0.230 |
| D7 | Observed_species | 115 (70, 244.5) | 264 (208.8, 337.8) | 2.921 | 0.003 |
|  | Chao1 | 121.7 (84.8, 319.8) | 369.4 (317.5, 503.8) | 3.328 | 0.001 |
|  | ACE | 126.1 (91.7, 338.7) | 412.5 (307.0, 540.0) | 3.221 | 0.001 |
|  | Shannon | 1.61 (0.76, 3.03) | 2.13 (1.72, 2.61) | 0.708 | 0.479 |
|  | Simpson | 0.47 (0.19, 0.75) | 0.56 (0.46, 0.67) | 0.53 | 0.600 |

**Supplementary Table 3.** Anosim of the SGA and AGA groups on days 1, 3, 5, and 7.

| **Days** | **R-value** | **P-value** |
| --- | --- | --- |
| D1 | 0.2102 | 0.001 |
| D3 | 0.1762 | 0.004 |
| D5 | 0.3667 | 0.001 |
| D7 | 0.5273 | 0.002 |

**Supplementary Table 4.** List of abbreviations.

| **Abbreviation** | **Full name** |
| --- | --- |
| SGA | Small for Gestational Age |
| AGA | Appropriate for Gestational Age |
| ASQ-3 | Ages and Stages Questionnaires-3 |
| ASD | Autism Spectrum Disorders |
| PCR | Polymerase Chain Reaction |
| OTUs | Operational Taxonomic Units |
| ACE | Abundance-based Coverage Estimator |
| AD | Alzheimer's Disease |
| rDNA | Ribosome Deoxyribonucleic Acid |
| PCoA | Principal Co-ordinates Analysis |
| NMDS | Non-Metric Multi-Dimensional Scaling |
| Anosim | Analysis of Similarities |
| LEfSe | Linear Discriminant Analysis Effect Size |
| LDA | Linear Discriminant Analysis |
| SPSS | Statistical Package for Social Sciences |
| ANOVA | Analysis of Variance |
| PD | Parkinson’s disease |
| DM | Diabetes mellitus |
| GDM | Gestational diabetes mellitus |
| MS | Multiple Sclerosis |
| EAE | Experimental Autoimmune Encephalomyelitis |
| ETBF | Enterotoxigenic *B. fragilis* |
| NTBF | Nontoxigenic *B. fragilis* |
| PSA | polysaccharide A |
| SCFAs | Short-Chain Fatty Acids |
| BDNF | Brain-Derived Neurotrophic Factor |
| ALS | Amyotrophic Lateral Sclerosis |
| CS | caesarean section |

**Supplementary Table 5.** Comparison of alpha diversity between the good and poor communication score groups on days 1, 3, 5, and 7.

| **Days** | **Alpha index** | **Good communication score group** | **Poor communication score group** | **z** | ***P*** |
| --- | --- | --- | --- | --- | --- |
| D1 | Observed_species | 220 (118, 292.5) | 530 (146.3, 682) | 1.186 | 0.236 |
|  | Chao1 | 263 (145.1, 340.8) | 637.2 (16.4, 787.6) | 1.334 | 0.182 |
|  | ACE | 300.6 (153.6, 373.1) | 706.4 (218.8, 879.3) | 1.334 | 0.182 |
|  | Shannon | 2.04 (1.41, 2.69) | 1.86 (0.82, 4.94) | 0.222 | 0.824 |
|  | Simpson | 0.59 (0.47, 0.67) | 0.38 (0.17, 0.83) | 0.964 | 0.335 |
| D3 | Observed_species | 202 (79.5, 240) | 271 (242, 398.5) | 2.310 | 0.021 |
|  | Chao1 | 234.2 (91.7, 288.1) | 343.7 (289.0, 456.7) | 2.309 | 0.021 |
|  | ACE | 238.6 (93.9, 283.5) | 314.4 (278.1, 448.5) | 2.309 | 0.021 |
|  | Shannon | 1.61 (0.84, 2.79) | 2.66 (2.13, 4.77) | 1.92 | 0.055 |
|  | Simpson | 0.39 (0.19, 0.67) | 0.65 (0.51, 0.89) | 1.697 | 0.090 |
| D5 | Observed_species | 206 (80, 333.5) | 224.5 (88.3, 275.3) | 0.07 | 0.944 |
|  | Chao1 | 270 (110.4, 414.6) | 282.5 (126.0, 348.2) | 0 | >0.999 |
|  | ACE | 322 (121.0, 471.6) | 317.2 (136.7, 388.6) | 0.07 | 0.944 |
|  | Shannon | 1.68 (1.02, 3.21) | 2.05 (0.80, 2.70) | 0.35 | 0.726 |
|  | Simpson | 0.56 (0.26, 0.81) | 0.48 (0.20, 0.67) | 0.7 | 0.484 |
| D7 | Observed_species | 88 (62.5, 196.8) | 202 (99, 216.5) | 0.577 | 0.564 |
|  | Chao1 | 90.5 (63.7, 206.0) | 208.2 (103.0,219.6) | 0.577 | 0.564 |
|  | ACE | 93.1 (65.6, 211.9) | 217.2 (108.7, 225.2) | 0.577 | 0.564 |
|  | Shannon | 2.00 (0.95, 2.93) | 2.24 (0.73, 3.63) | 0 | >0.999 |
|  | Simpson | 0.57 (0.26, 0.77) | 0.51 (0.15, 0.83) | 0 | >0.999 |

**Supplementary Table 6.** Anosim of the good and poor communication score groups on days 1, 3, 5, and 7.

| **Days** | **R-value** | **P-value** |
| --- | --- | --- |
| D1 | 0.0296 | 0.302 |
| D3 | ‒0.0579 | 0.626 |
| D5 | ‒0.0105 | 0.504 |
| D7 | 0.0156 | 0.460 |

**Supplementary Table 7.** Gut microbiota analysis of the poor and good communication score groups on days 1, 3, 5, and 7 at levels of phylum, family, genus, and species.

| **Taxonomy** | **Days** | **Microbiota** | **Poor communication score group** | **Good communication score group** | ***P****<***0.05** |
| --- | --- | --- | --- | --- | --- |
| Phylum | D7 | *Bacteroidota* | 9.92×10^-5^ | 5.11×10^-3^ | 0.012 |
| Family | D1 | *Streptococcaceae* | 3.25×10^-2^ | 1.35×10^-1^ | 0.004 |
|  |  | *Enterobacteriaceae* | 6.53×10^-3^ | 9.58×10^-2^ | 0.042 |
|  | D5 | *Staphylococcaceae* | 5.06×10^-3^ | 2.37×10^-2^ | 0.033 |
| Genus | D1 | *Streptococcus* | 2.93×10^-2^ | 1.33×10^-1^ | 0.003 |
|  | D5 | *Staphylococcus* | 5.04×10^-3^ | 2.37×10^-2^ | 0.027 |
|  |  | *Enterococcus* | 7.83×10^-1^ | 6.15×10^-1^ | 0.048 |
|  | D7 | *Bacteroides* | 7.08×10^-5^ | 2.73×10^-3^ | 0.005 |
|  |  | *Corynebacterium* | 3.05×10^-4^ | 7.08×10^-6^ | 0.033 |
| Species | D7 | *Bacteroides_fragilis* | 7.08×10^-6^ | 2.21×10^-3^ | 0.023 |

**Supplementary Table 8.** Correlation analysis between communication scores at 6 months postage and differential microbiota of SGA infants with different communication performance.

| **Days and microbiota** | **ASQ-3 scores** | **Correlation index (*r*)** | ***P*** |
| --- | --- | --- | --- |
| D1 *f-Streptococcaceae* | Communication | -0.004 | 0.986 |
| D1 *f-Enterobacteriaceae* | Communication | 0.159 | 0.446 |
| D1 *g-Streptococcus* | Communication | 0.061 | 0.772 |
| D5 *f-Staphylococcaceae* | Communication | 0.011 | 0.961 |
| D5 *g-Staphylococcus* | Communication | 0.013 | 0.953 |
| D5 *g-Enterococcus* | Communication | -0.246 | 0.258 |
| D7 *p-Bacteroidota* | Communication | 0.895 | 0.003 |
| D7 *g-Bacteroides* | Communication | 0.875 | 0.004 |
| D7 *s-Bacteroides_fragilis* | Communication | 0.886 | 0.003 |
| D7 *g-Corynebacterium* | Communication | -0.510 | 0.197 |

**Supplementary Table 9.** Clinical characteristics of the caesarean birth and vaginal delivery groups in the SGA population.

| **Descriptive Variable** | **Vaginal delivery group** | **Caesarean birth group** | **Statistic value** | ***P*** |
| --- | --- | --- | --- | --- |
| Male | 6 (35.3%) | 13 (54.2%) | 1.425 | 0.233 |
| Gestational age (weeks) | 37.9 (3.0) | 37.6 (1.3) | -1.304 | 0.192 |
| Birthweight (grams) | 2432.4 ± 285.7 | 2304.6 ± 324.1 | 1.306 | 0.199 |
| Ampicillin to neonates (first week) | 12 (70.6%) | 19 (79.2%) | 0.068 | 0.794 |
| Mixed fed (formula + breast-feeding) | 17 (100%) | 24 (100%) | - | >0.999 |

**Supplementary Table 10.** Comparison of alpha diversity between the caesarean birth and vaginal delivery groups on days 1, 3, and 5.

| **Days** | **Alpha index** | **Vaginal delivery group** | **Caesarean birth group** | **z** | ***P*** |
| --- | --- | --- | --- | --- | --- |
| D1 | Observed_species | 172 (150) | 262 (360) | -1.415 | 0.157 |
|  | Chao1 | 181.75 (164.29) | 341.92 (433.51) | -1.415 | 0.157 |
|  | ACE | 188.65 (174.65) | 367.93 (498.72) | -1.464 | 0.143 |
|  | Shannon | 1.75 (1.19) | 2.16 (1.13) | -1.561 | 0.118 |
|  | Simpson | 0.47 (0.39) | 0.60 (0.28) | -1.073 | 0.283 |
| D3 | Observed_species | 195.5 (260) | 220.5 (85) | -0.665 | 0.506 |
|  | Chao1 | 213.57 (314.7) | 255.79 (100.24) | -0.684 | 0.494 |
|  | ACE | 216.84 (303.33) | 251.95 (102.96) | -0.684 | 0.494 |
|  | Shannon | 2.14 (2.27) | 1.66 (1.83) | -0.266 | 0.790 |
|  | Simpson | 0.65 (0.55) | 0.38 (0.43) | -0.114 | 0.909 |
| D5 | Observed_species | 222.5 (207) | 190 (182) | -0.103 | 0.918 |
|  | Chao1 | 287.99 (277.23) | 247.75 (227.3) | -0.411 | 0.681 |
|  | ACE | 326.29 (324.27) | 278.13 (249.46) | -0.411 | 0.681 |
|  | Shannon | 1.5 (2.54) | 1.69 (1.61) | -0.360 | 0.719 |
|  | Simpson | 0.46 (0.62) | 0.49 (0.45) | -0.463 | 0.643 |

**Supplementary Table 11.** Anosim of the caesarean birth and vaginal delivery groups on days 1, 3, and 5.

| **Days** | **R-value** | **P-value** |
| --- | --- | --- |
| D1 | 0.0589 | 0.142 |
| D3 | 0.0562 | 0.118 |
| D5 | 0.0109 | 0.298 |

**Supplementary Table 12.** Intestinal microbiota analysis of the caesarean birth and vaginal delivery groups on days 1, 3, and 5 at levels of phylum, family, and genus.

| **Taxonomy** | **Days** | **Microbiota** | **Vaginal delivery group** | **Caesarean birth group** | ***P*** |
| --- | --- | --- | --- | --- | --- |
| Phylum | D1 | *Campylobacterota* | 1.30×10^-3^ | 7.38×10^-5^ | 0.024 |
|  | D3 | *Bacteroidota* | 5.59×10^-2^ | 2.04×10^-3^ | 0.047 |
|  | D5 | *Bacteroidota* | 7.53×10^-2^ | 1.47×10^-3^ | 0.016 |
|  |  | *Actinobacteria* | 1.34×10^-2^ | 6.00×10^-2^ | 0.038 |
| Family | D3 | *Bacteroidaceae* | 5.47×10^-2^ | 4.24×10^-4^ | 0.005 |
|  | D5 | *Bacteroidaceae* | 7.16×10^-2^ | 8.43×10^-4^ | 0.001 |
|  |  | *Tannerellaceae* | 3.03×10^-3^ | 1.49×10^-5^ | 0.036 |
| Genus | D1 | *Ileibacterium* | 2.28×10^-3^ | 6.94×10^-5^ | 0.018 |
|  | D3 | *Bacteroides* | 5.47×10^-2^ | 4.24×10^-4^ | 0.005 |
|  |  | *Aminobacter* | 0.00 | 1.67×10^-3^ | 0.001 |
|  |  | *Georgenia* | 0.00 | 7.94×10^-4^ | 0.001 |
|  |  | *Fastidiosipila* | 0.00 | 4.07×10^-4^ | 0.001 |
|  |  | *Loigolactobacillus* | 0.00 | 4.64×10^-4^ | 0.001 |
|  | D5 | *Bacteroides* | 7.16×10^-2^ | 8.43×10^-4^ | 0.001 |
|  |  | *[Eubacterium]_hallii_group* | 0.00 | 7.80×10^-5^ | 0.001 |

**Supplementary Table 13.** Comparison of ASQ-3 scores between the caesarean birth and vaginal delivery groups

| **Delivery mode** | **Communication** | **Gross motor** | **Fine motor** | **Problem solving** | **Personal-social** |
| --- | --- | --- | --- | --- | --- |
| Vaginal delivery group | 60 (18) | 55 (18) | 55 (13) | 60 (10) | 55 (15) |
| Caesarean birth group | 55 (18) | 60 (10) | 60 (10) | 60 (10) | 55 (10) |
| Statistic value | -0.827 | -0.751 | -0.675 | -0.758 | -0.351 |
| *P* | 0.408 | 0.453 | 0.500 | 0.448 | 0.726 |

**Supplementary Table 14.** Clinical characteristics of the group with antibiotics and the group without antibiotics in the SGA population.

| **Descriptive Variable** | **Group without antibiotics** | **Group with antibiotics** | **Statistic value** | ***P*** |
| --- | --- | --- | --- | --- |
| Male | 6 (60.0%) | 13 (41.9%) | 0.399 | 0.528 |
| Gestational age (weeks) | 37.4 (1.1) | 37.6 (2.7) | -1.511 | 0.131 |
| Birthweight (grams) | 2264.0 ± 144.9 | 2387.7 ± 345.0 | -1.606 | 0.117 |
| Cesarean | 5 (50%) | 19 (61.3%) | 0.068 | 0.794 |
| Mixed fed (formula + breast-feeding) | 10 (100%) | 31 (100%) | - | >0.999 |

**Supplementary Table 15.** Comparison of alpha diversity between the group with antibiotics and the group without antibiotics on days 1, 3, 5 and 7.

| **Days** | **Alpha index** | **Group without antibiotics** | **Group with antibiotics** | **z** | ***P*** |
| --- | --- | --- | --- | --- | --- |
| D1 | Observed_species | 386 (261) | 189 (209) | -1.411 | 0.158 |
|  | Chao1 | 432.10 (351.04) | 205.56 (254.72) | -1.549 | 0.121 |
|  | ACE | 478.12 (416.73) | 221.23 (284.29) | -1.494 | 0.135 |
|  | Shannon | 1.63 (0.71) | 2.10 (1.37) | -0.941 | 0.347 |
|  | Simpson | 0.34 (0.35) | 0.59 (0.20) | -1.383 | 0.167 |
| D3 | Observed_species | 270 (135) | 201 (174) | -1.698 | 0.090 |
|  | Chao1 | 313.66 (149.50) | 233.76 (188.38) | -1.739 | 0.082 |
|  | ACE | 319.49 (152.13) | 233.31 (192.13) | -1.614 | 0.107 |
|  | Shannon | 2.72 (2.03) | 1.60 (2.08) | -1.781 | 0.075 |
|  | Simpson | 0.68 (0.48) | 0.38 (0.49) | -1.781 | 0.075 |
| D5 | Observed_species | 227 (140) | 178 (209) | -0.528 | 0.598 |
|  | Chao1 | 288.40 (209.31) | 271.58 (294.42) | -0.389 | 0.697 |
|  | ACE | 319.34 (240.40) | 297.94 (320.53) | -0.167 | 0.868 |
|  | Shannon | 2.63 (1.68) | 1.36 (1.83) | -1.667 | 0.096 |
|  | Simpson | 0.64 (0.40) | 0.32 (0.48) | -1.778 | 0.075 |

**Supplementary Table 16.** Anosim of the group with antibiotics and the group without antibiotics on days 1, 3, and 5.

| **Days** | **R-value** | **P-value** |
| --- | --- | --- |
| D1 | 0.0379 | 0.226 |
| D3 | -0.0007 | 0.416 |
| D5 | 0.0613 | 0.129 |

**Supplementary Table 17.** Gut microbiota analysis of the group with antibiotics and the group without antibiotics on days 1, 3, and 5 at levels of phylum, family, and genus.

| **Taxonomy** | **Days** | **Microbiota** | **Group without antibiotics** | **Group with antibiotics** | ***P*** |
| --- | --- | --- | --- | --- | --- |
| Phylum | D5 | *Gemmatimonadota* | 0.00 | 4.04×10^-5^ | 0.001 |
| Family | D1 | *Micrococcaceae* | 1.30×10^-3^ | 2.22×10^-2^ | 0.049 |
|  | D3 | *Carnobacteriaceae* | 0.00 | 4.52×10^-3^ | 0.004 |
|  | D5 | *Enterococcaceae* | 4.77×10^-1^ | 7.45×10^-1^ | 0.021 |
|  |  | *Enterobacteriaceae* | 2.09×10^-1^ | 2.59×10^-3^ | 0.003 |
|  |  | *Burkholderiaceae* | 1.54×10^-3^ | 1.28×10^-2^ | 0.030 |
| Genus | D1 | *Lactobacillus* | 1.42×10^-4^ | 1.39×10^-3^ | 0.025 |
|  |  | *Halomonas* | 7.28×10^-5^ | 1.60×10^-3^ | 0.041 |
|  | D3 | *Carnobacterium* | 0.00 | 4.37×10^-3^ | 0.004 |
|  |  | *Aminobacter* | 0.00 | 1.31×10^-3^ | 0.001 |
|  |  | *Romboutsia* | 0.00 | 1.36×10^-3^ | 0.006 |
|  |  | *Georgenia* | 0.00 | 6.20×10^-4^ | 0.001 |
|  |  | *Aliidiomarina* | 0.00 | 5.03×10^-4^ | 0.002 |
|  |  | *Cetobacterium* | 0.00 | 3.83×10^-4^ | 0.004 |
|  |  | *Loigolactobacillus* | 0.00 | 3.63×10^-4^ | 0.001 |
|  | D5 | *Alloprevotella* | 0.00 | 8.38×10^-5^ | 0.006 |

**Supplementary Table 18.** Comparison of ASQ-3 scores between the group with antibiotics and the group without antibiotics.

| **Application of antibiotics** | **Communication** | **Gross motor** | **Fine motor** | **Problem solving** | **Personal-social** |
| --- | --- | --- | --- | --- | --- |
| Group without antibiotics | 55 (21) | 55 (16) | 57.5 (10) | 55 (10) | 52.5 (11) |
| Group with antibiotics | 60 (14) | 60 (10) | 55 (10) | 60 (10) | 55 (10) |
| Statistic value | -0.449 | -0.451 | -0.319 | -0.465 | -0.017 |
| *P* | 0.653 | 0.652 | 0.750 | 0.642 | 0.986 |
